# Distinct and targetable role of calcium-sensing receptor in leukaemia

**DOI:** 10.1038/s41467-023-41770-0

**Published:** 2023-10-06

**Authors:** Raquel S. Pereira, Rahul Kumar, Alessia Cais, Lara Paulini, Alisa Kahler, Jimena Bravo, Valentina R. Minciacchi, Theresa Krack, Eric Kowarz, Costanza Zanetti, Parimala Sonika Godavarthy, Fabian Hoeller, Pablo Llavona, Tabea Stark, Georg Tascher, Daniel Nowak, Eshwar Meduri, Brian J. P. Huntly, Christian Münch, Francesco Pampaloni, Rolf Marschalek, Daniela S. Krause

**Affiliations:** 1 Georg-Speyer-Haus, Institute for Tumor Biology and Experimental Therapy, Frankfurt am Main, Germany; 2 Pediatric Neurooncology, Hopp Children’s Cancer Center Heidelberg (KiTZ) and German Cancer Research Center (DKFZ), Heidelberg, Germany; 3 Institute of Pharmaceutical Biology, Goethe University, Frankfurt am Main, Germany; 4 University Medical Center, Johannes Gutenberg University Mainz, Mainz, Germany; 5 Institute of Molecular Biology gGmbH (IMB), Mainz, Germany; 6 Institute of Biochemistry II, Faculty of Medicine, Goethe University, Frankfurt am Main, Germany; 7 Department of Hematology and Oncology, Medical Faculty Mannheim, Heidelberg University, Mannheim, Germany; 8 Department of Haematology, University of Cambridge, Cambridge, United Kingdom; 9 Buchmann Institute for Molecular Life Sciences (BMLS, CEF-MC), Goethe University, Frankfurt am Main, Germany; 10 Institute of General Pharmacology and Toxicology, Goethe-University, Frankfurt am Main, Germany; 11 German Cancer Research Center (DKFZ), Heidelberg, Germany; 12 German Cancer Consortium (DKTK), Heidelberg, Germany; 13 Frankfurt Cancer Institute, Frankfurt, Germany

**Keywords:** Cancer microenvironment, Cancer stem cells

## Abstract

Haematopoietic stem cells (HSC) reside in the bone marrow microenvironment (BMM), where they respond to extracellular calcium [eCa^2+^] via the G-protein coupled calcium-sensing receptor (CaSR). Here we show that a calcium gradient exists in this BMM, and that [eCa^2+^] and response to [eCa^2+^] differ between leukaemias. CaSR influences the location of MLL-AF9^+^ acute myeloid leukaemia (AML) cells within this niche and differentially impacts MLL-AF9^+^ AML versus BCR-ABL1^+^ leukaemias. Deficiency of CaSR reduces AML leukaemic stem cells (LSC) 6.5-fold. CaSR interacts with filamin A, a crosslinker of actin filaments, affects stemness-associated factors and modulates pERK, *β*-catenin and c-MYC signaling and intracellular levels of [Ca^2+^] in MLL-AF9^+^ AML cells. Combination treatment of cytarabine plus CaSR-inhibition in various models may be superior to cytarabine alone. Our studies suggest CaSR to be a differential and targetable factor in leukaemia progression influencing self-renewal of AML LSC via [eCa^2+^] cues from the BMM.

## Abstract (web summary)

Several parameters in the bone marrow (BM) niche regulate leukaemic stem cell status and disease progression. Here, the authors show that calcium-sensing receptor affects the location of acute myeloid leukaemia (AML) cells in the BM niche which influences self-renewal of leukaemic stem cells and AML development

## Introduction

Fetal haematopoiesis occurs in the bone marrow (BM), once haematopoietic stem cells (HSC) have migrated there from the fetal liver shortly before birth^1^. In the BM and its surrounding microenvironment (BMM) HSC are regulated by cellular and acellular components^2^. While most attention, so far, has focused on the role of mesenchymal stem cells, macrophages and other cell types for maintaining HSC homoeostasis, HSC are also attracted to the endosteum of the bone during development via the local calcium ions^3^ and are preferentially localised in BM niches with higher calcium concentrations^4^.

The bone, which stores 99% of a mammal’s ionised calcium, primarily in the form of calcium phosphate^5^, releases calcium ions during bone remodelling^6^. While the role of intracellular calcium signalling, for instance as cofactor for signalling processes^7^, or, extracellularly, as a cofactor of various enzymatic reactions^8^, have been well studied, the role of extracellular calcium (eCa^2+^) on cellular physiology has received far less attention. However, the culture of HSC in low calcium concentrations, for instance, seems to improve their maintenance^9^.

HSC sense the extracellular calcium ion concentration via the ubiquitously expressed seven-transmembrane-spanning G-protein-coupled calcium-sensing receptor (CaSR), guiding HSC adhesion to extracellular matrix (ECM) proteins, lodgement and engraftment in the BM niche^3,10^. Depending on the cellular context, CaSR binds to multiple ligands and signals via broadly divergent signalling pathways^11^. In disease settings, CaSR has been associated with hyperparathyroidism, but also cardiovascular disease^12^ and cancer^11^, where, tumour-dependently, it may act as a tumour suppressor or oncogene^11,13^. However, its role in signalling in leukaemia and leukaemia stem cells (LSC), the malignant counterparts to HSC, is largely unknown.

In parallel to HSC, the BMM also has a distinct and supportive role in the maintenance and progression of leukaemias via the creation of a leukaemia-specific self-enforcing niche^14–16^ and the support of LSCs^2^. This influences LSC quiescence and differentiation, both of which are closely linked to successful treatment^17^, therapy resistance^18^ and probability of relapse^2^.

Given these findings on calcium and the recently discovered role of the local concentration of another chemical factor in the BMM, adenosine triphosphate (ATP), on LSC in acute myeloid leukaemia (AML)^19^, we hypothesised that calcium ions released from remodelling bone, subject to a fine balance between osteoblasts and osteoclasts, and/or CaSR, may contribute to leukaemia development, progression and response to therapy.

Here, we show that calcium ions in the BMM form a gradient, sensed by CaSR and influencing AML cell localisation. As shown in multiple in vitro and in vivo models, CaSR and its downstream signalling play a crucial and differential role in various leukaemia types, contributing to disease progression and self-renewal capabilities of LSC in AML. Pharmacological modification of CaSR in combination with standard chemotherapy is an effective therapeutic strategy, suggesting that an improvement of existing treatments in AML, a still intractable disease, may be possible via the modification of chemical factors in the BMM.

## Results

### Calcium ions as features of the normal and leukaemic BMM

**Fig. 1:**
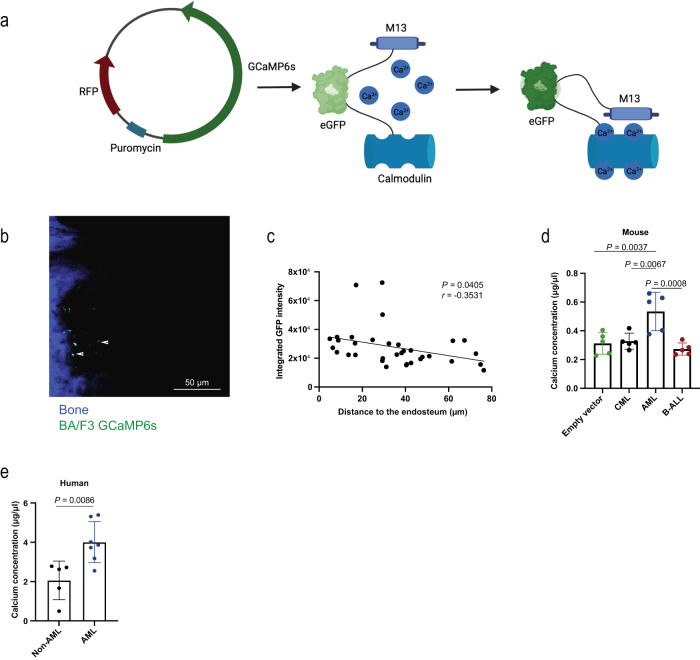
Calcium ions as features of the normal and leukaemic BMM. **a** Schematic diagram (Created with BioRender.com) of the calcium indicator GCaMP6s, cloned into the sleeping beauty transposon plasmid pSBbi-Pur expressing RFP (left). The mechanism of GFP fluorescence after the binding of calcium ions to calmodulin and the conformational change of calmodulin and M13 peptide are shown on the right. **b** Representative two-photon intravital microscopy image of the calvarium of an unirradiated Rag-2^−/−^ IL2R γ^−/−^ CD47^−/−^ mouse, which had been transplanted with 5 × 10^5^ BA/F3 cells expressing GCaMP6s 2 h prior to imaging. The image is representative of 3 independent experiments, and the scale bar represents 50 μm. Bone fluoresces blue due to second harmonic generation, the transplanted BA/F3-GCaMP6s cells fluoresce green. **c** Correlation of the integrated GFP intensity of BA/F3-GCaMP6s cells with the distance to the endosteum (μm), as measured by intravital microscopy. In stacks of 7−17 images of an individual BA/F3-GCaMP6s cell, the GFP intensity of the middle stack was used as representative value. Pearson’s correlation coefficient (*r*) and the *P* value are shown *(P* = 0.0405, *n* = 34). The *n* represents 34 individual cells which were analysed. The experiments were repeated three times. **d** Quantification of the calcium concentration (μg/μl) present in the bone marrow (BM) of irradiated BALB/c mice transplanted with empty vector control-transduced BM or moribund mice with chronic myeloid leukaemia (CML), acute myeloid leukaemia (AML) or B-cell acute lymphoblastic leukaemia (B-ALL) shortly before death. All diseases were induced with the retroviral transduction/transplantation model (*n* = 5 individual mice per group, one-way ANOVA, Tukey test, mean ± SD). **e** Concentration of calcium (μg/μl) present in the heparinised BM plasma of patients (AML (*n* = 7); non-AML: CML (*n* = 1), myelodysplastic syndrome (MDS) (*n* = 1), ALL (*n* = 2), diffuse large B-cell lymphoma (DLBCL) (*n* = 1), *P* = 0.0086, two-*t*ailed *t* test, mean ± SD). *n* refers to individual patients. Source data are provided as a Source Data file.

As a result of bone remodelling, calcium ions are released into the central BM cavity^6^. Consistently, the calcium concentration in the BMM of Col1-caPPR mice, characterised by osteoblastic cell-specific constitutive activation of the receptor for parathyroid hormone (PTH) and PTH-related peptide (PTHrp) and increased bone remodelling^14,20^, was significantly higher than in control mice (Supplementary Fig. 1a). We hypothesised that bone turnover creates a calcium gradient with higher calcium concentrations found close to the endosteum and lower concentrations in the central BM cavity. To test this, we used a genetically encoded fluorescent sensor as calcium indicator, GCaMP6s, which increases its green fluorescent protein (GFP) intensity due to conformational changes upon binding of calcium ions^21^ (Fig. 1a). Indeed, exposure of the murine pro-B cell line BA/F3 or the leukaemic cell lines THP1 (MLL-AF9^+^; AML) or K562 (BCR-ABL1^+^; chronic myeloid leukaemia (CML)), stably transfected with this construct, to increasing calcium concentrations enhanced GFP fluorescence (Supplementary Fig. 1b–g, Supplementary Table 1). In THP1 cells the increase in GFP fluorescence was also statistically significant at a calcium ion concentration between 0.5–1.5 mM, representing the physiological calcium concentration at the endosteum^4^ (Supplementary Fig. 1c). Transplantation of GCaMP6s^+^ BA/F3 cells into mice and visualisation of the transplanted cells in the calvarium by intravital microscopy (IVM)^22^ revealed a significantly higher calcium concentration close to the endosteum, which decreased with increasing distance from bone (*P* = 0.0405, Fig. 1b, c). Contrastingly, the calcium ion-independent red or green fluorescence of the GCaMP6s or MSCV IRES GFP constructs in BA/F3 cells, respectively, did not change with increasing distance from bone (Supplementary Fig. 1h, i), suggesting that the decreasing GFP fluorescence with increasing distance from bone was not merely due to signal attenuation with increased distance to bone.

Further, the calcium concentration was highest in mice (*P* = 0.0037, Fig. 1d) and humans (*P* = 0.0086, Fig. 1e) with AML compared to other haematological malignancies or mice transplanted with empty vector-transduced bone marrow. However, we found no alterations of the calcium concentration in the medium after in vitro culture of THP1 or K562 cells (Supplementary Fig. S2a, b). Levels of PTH and PTHrp, which may influence the calcium concentration, in the bone marrow supernatant of mice with murine AML or CML, generated via the retroviral transduction/transplantation model^14^, were similar (Supplementary Fig. 2c, d). Bone formation, as measured by procollagen type I N-propeptide (PINP) in the bone marrow supernatant, but not bone degradation, was significantly higher in murine AML compared to CML (Supplementary Fig. 2e, f).

Taken together, these results suggest that a calcium gradient may exist in the BMM, possibly distinctly affecting the localisation of haematopoietic cells. Additionally, the calcium concentration in the BMM differs between haematological malignancies in mice and humans and is highest in AML.

### Calcium and CaSR impact leukaemia cell function

**Fig. 2:**
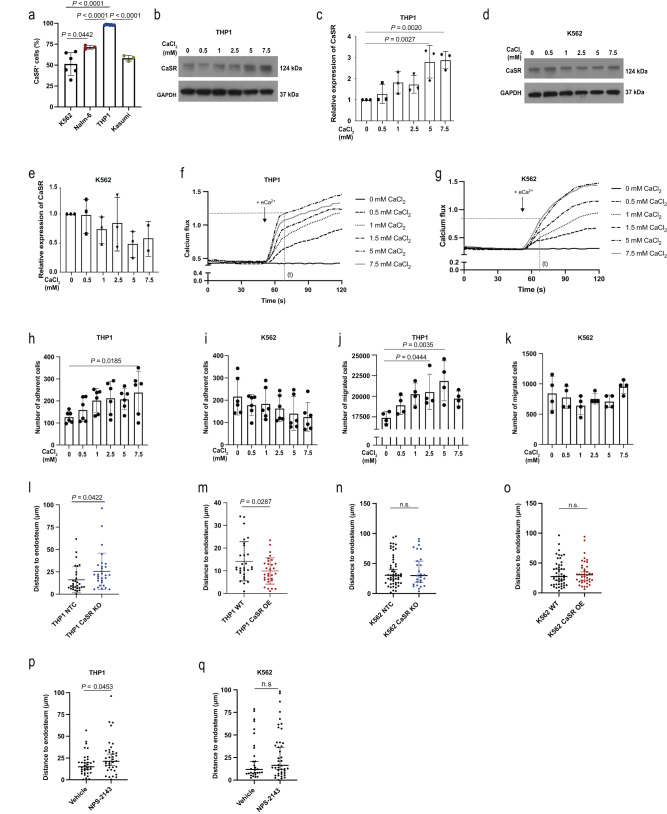
Calcium and CaSR impact leukaemia cell function. **a** Percentage of CaSR^+^ cells of all cells in different human leukaemia cell lines (one-way ANOVA, Dunnett test, mean ± SD) (K562: BCR-ABL1^+^ (CML, *n* = 9), NALM-6: B-ALL (*n* = 3), THP1: MLL-AF9 (AML, *n* = 8), Kasumi: AML1-ETO (AML, *n* = 3)). *n* refers to biological replicates. **b**, **c** Representative immunoblot (**b**) and its quantification (**c**) (mean ± SD) for CaSR and GAPDH expression in lysates of THP1 cells treated with 0–7.5 mM of CaCl_2_ for 4 h. The blot is representative of three independent experiments. **d**, **e** Representative immunoblot (of three independent experiments) (**d**) and its quantification (**e**) (mean ± SD) for CaSR and GAPDH expression in K562 cells treated with CaCl_2_. **f**, **g** Calcium flux analysis of THP1 (**f**) and K562 (**g**). **h**, **i** Number of THP1 (**h**) or K562 (**i**) cells adhering to fibronectin (FN). Prior to adhesion, cells had been treated with calcium (*n* = 6, one-way ANOVA, Dunnett test, mean ± SD). **j**, **k** Number of THP1 (**j**) or K562 (**k**) cells which migrated towards C-X-C motif chemokine 12 (CXCL12). Cells had been pre-treated with calcium (*n* = 4, one-way ANOVA, Dunnett test, mean ± SD). From **h**–**k**, *n* refers to biological replicates. **l**–**o** Distance (in µm) of intravenously transplanted CMTMR-labelled THP1 (**l**, **m**) or K562 (**n**, **o**) cells to the endosteum in the calvarium of NOD SCID interleukin-2 receptor γ (IL2Rγ)^−/−^ (NSG) mice. NTC (non-target control) (black) versus CaSR knockout (KO) (blue) cells (**l**, **n**) and THP1 WT (black) versus CaSR-overexpressing (OE) THP1 or K562 (red) (m,o) cells had been transplanted and imaged by intravital microscopy (two-tailed *t* test, mean ± SD) (*n* = 30 in (**l**), *n* = 29 (THP1 WT), *n* = 30 (THP1 CaSR OE) in **m**, *n* = 27 (K562 NTC), *n* = 53 (K562 CaSR KO) in **n**, *n* = 40 (K562 WT), *n* = 50 (K562 CaSR OE) in **o**. **p**, **q** Distance of transplanted THP1 (**p**) or K562 (**q**) cells to the endosteum. Cells had been pre-treated with NPS-2143. Experiments were performed in three different mice per cohort (*n* = 35 (vehicle), *n* = 38 (NPS-2143) in **p**, *n* = 30 (vehicle), *n* = 42 (NPS-2143) in **q**). From **l**–**q** *n* refers to individual cells. Data are presented as mean values ± SD. Source data are provided as a Source Data file.

Hypothesising that differences in the extracellular calcium concentration may influence pathophysiological features of normal haematopoietic and leukaemia cells, possibly via CaSR, we, firstly, confirmed^3^ that CaSR levels were higher on normal murine haematopoietic stem cells compared to more differentiated haematopoietic cells (Supplementary Fig. 3a–c). In BM, the highest expression of CaSR was found on CD11b^+^ myeloid cells, while in peripheral blood (PB) and spleen, highest expression of CaSR was found on B220^+^ B cells (Supplementary Fig. 3d–f). In murine AML, CML and B-cell acute lymphoblastic leukaemia (B-ALL), CaSR expression was highest on the respective putative leukaemic stem cells, i.e. lineage (Lin)^−^ or granulocyte-monocyte progenitor (GMP) cells in MLL-AF9^+^ AML^23^, Lin^−^ c-Kit^+^ Sca-1^+^ (LKS) CD150^+^ CD48^−^ (SLAM) cells in CML^24^ and BP-1^+^ pre-B cells in B-ALL^22^ (Supplementary Fig. 3g–i). On human leukaemia cells, the percentage of CaSR^+^ cells was highest in MLL-AF9^+^ THP1 (AML) cells compared to AML cells expressing a different oncoprotein (Kasumi), NALM-6 (B-ALL) or K562 (CML) cells (*P* < 0.0001, Fig. 2a, Supplementary Table 2). Exposure of THP1 and BA/F3 (*P* = 0.002, Fig. 2b, c, Supplementary Fig. 4a, b), but not K562 (Fig. 2d, e, Supplementary Fig. 4c) cells to increasing calcium concentrations led to higher expression of CaSR. These calcium concentrations and exposure times (4 h) were chosen, as they cover the physiological ranges of calcium close to the endosteum (0.5–1.5 mM)^4^ and allow transcriptional/translational changes, respectively.

Testing calcium influx into different leukaemia cells, we demonstrated that intracellular calcium levels in THP1 cells 70 s after exposure to 5 or 7.5 mM extracellular calcium were significantly higher compared to K562 cells (Fig. 2f, g, Supplementary Fig. 4d–f). Functionally, adhesion to the extracellular matrix protein fibronectin (*P* = 0.0185, Fig. 2h, i) and migration towards the chemoattractant C-X-C motif chemokine 12 (CXCL12) (*P* = 0.0035, Fig. 2j, k) were increased when THP1, but not K562 cells had previously been exposed to higher calcium concentrations. Consistently, the percentage of THP1, but not K562 cells positive for C-X-C chemokine receptor type 4 (CXCR4), the receptor for CXCL12, increased in rising calcium concentrations (Supplementary Fig. 4g, h). Cell cycle status (Supplementary Fig. 4i, j) and apoptosis (Supplementary Fig. 4k, l) in response to increasing calcium concentrations did not differ between both cell lines. However, THP1 cells were found significantly closer to the endosteum than K562 cells (Supplementary Fig. 4m). Testing whether CaSR deficiency (*CASR* knockout (KO)) or overexpression (OE) may influence localisation in the BMM, we demonstrated by IVM that, indeed, *CASR* KO THP1 cells were located further away (*P* = 0.0422, Fig. 2l, Supplementary Fig. 5a–c), while *CASR* OE cells were located closer (*P* = 0.0287, Fig. 2m, Supplementary Fig. 5d–f) to the endosteum. Consistently, there was a significantly decreased migration of *CASR* KO THP1 cells and a trend towards increased migration of *CASR* OE THP1 cells to the stroma cell line HS-5 (Supplementary Fig. 5g), which expresses high levels of CXCL12 (Supplementary Fig. 5h). *CASR* KO K562 (Fig. 2n, Supplementary Fig. 5i–k) and *CASR* OE K562 (Fig. 2o, Supplementary Fig. 5l–n) cells were found at the same distance to the endosteum as controls. Furthermore, treatment of THP1, but not K562 cells, with the CaSR antagonist and calcilytic NPS-2143^25^ prior to transplantation and IVM led to significantly increased distance to the endosteum (*P* = 0.0453, Fig. 2p, q). Treatment of WT THP1 or *CASR* OE THP1 cells (Supplementary Fig. S5o–p), as well as WT K562 (Supplementary Fig. 5q, r) cells with NPS-2143 significantly decreased CXCR4 expression.

These data suggest that MLL-AF9^+^ versus BCR-ABL1^+^ leukaemia cells differ with regards to CaSR expression and sensitivity to calcium for cellular functions. Furthermore, CaSR or its inhibition by NPS-2143 impacts the localisation of THP1 cells in relation to the endosteum, whereby CXCR4 may play a contributory role.

### CaSR alters leukaemia progression

To understand the role of CaSR in normal and malignant haematopoiesis, we generated Mx1-Cre^−^^***/+***^ CaSR ^flox/flox^ (*Casr* KO) mice, in which *Casr* deletion is induced in haematopoietic cells after treatment with poly I:C (Supplementary Fig. 6a–c). Haematological analysis of PB, spleen and BM showed increased white blood cells in PB of non-leukaemic *Casr* KO compared to WT mice, while in BM and spleen myeloid cells were reduced. B cells were increased in BM (Supplementary Fig. 6d–k). The overall cellularity in the BM did not differ between WT and *Casr* KO mice, but numbers of CD11b^+^ cells in the BM were reduced (Supplementary Fig. 6l, m). No significant differences were observed for Lin^−^ c-Kit^+^ Sca-1^+^ (LKS) or LKS CD150^+^ CD48^−^ (LKS SLAM) cells in WT versus *Casr* KO mice (Supplementary Figs. 7 and 3a).

**Fig. 3:**
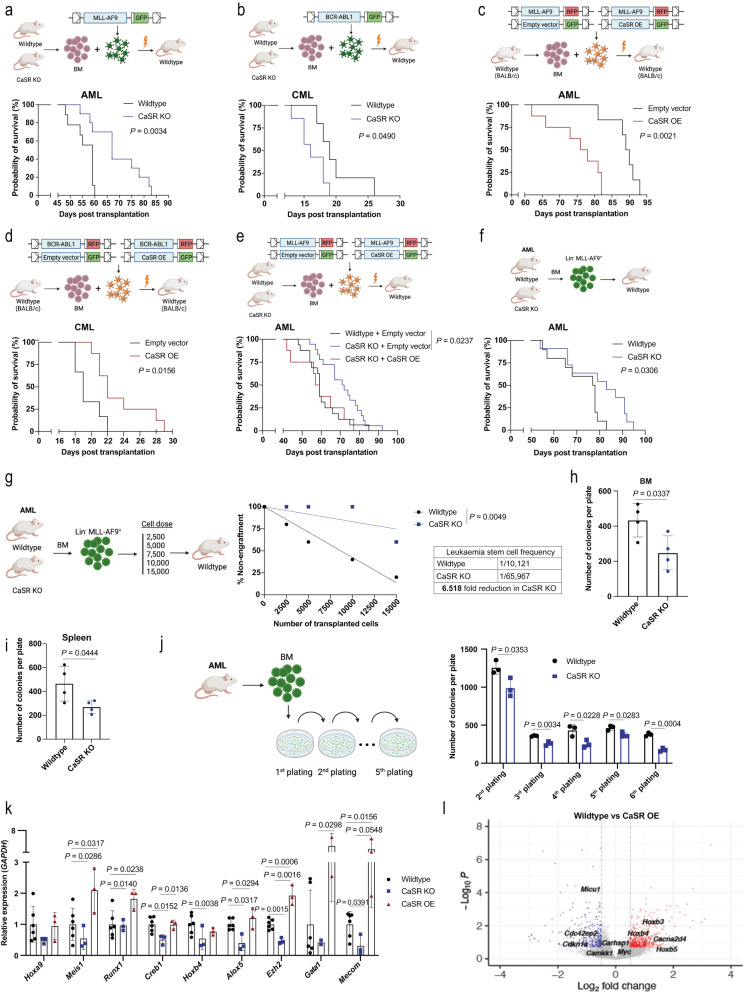
CaSR alters leukaemia progression and regulates self-renewal of AML LSC. **a**, **b** Kaplan–Meier style survival curves of mice transplanted with MLL-AF9^+^ (**a**; *n* = 9 (wildtype), *n* = 10 (CaSR KO), Log-rank test)) or BCR-ABL1^+^ (**b**; (*n* = 5 (wildtype), *n* = 7 (CaSR KO), Log-rank test)) wildtype (black) or CaSR KO (blue) BM. **c**, **d** Kaplan–Meier style survival curves of BALB/c mice transplanted with wildtype MLL-AF9^+^ (**c**) or BCR-ABL1 + -BM (**d**), co-transduced with empty vector- (black) or CaSR-overexpressing retrovirus (red) (*n* = 6 (empty vector), *n* = 8 (CaSR OE), Log-rank test). **e** Kaplan–Meier style survival curve of wildtype mice transplanted with wildtype or CaSR KO MLL-AF9^+^ BM. The wildtype donor BM was empty vector^+^ (black), and the CaSR KO BM was empty vector^+^ (blue) or overexpressed (OE) CaSR (red) (*n* = 16 (wildtype+empty vector), *n* = 16 (CaSR KO+empty vector), *n* = 8 (CaSR KO+CaSR OE), Log-rank test). **f** Kaplan-Meier-style survival curve of wildtype secondary mice transplanted with sorted MLL-AF9^+^ (GFP^+^) Lin^−^ BM cells from primary recipients of MLL-AF9-transduced wildtype or CaSR KO BM (*n* = 10 (wildtype), *n* = 11 (CaSR KO), Log-rank test). From **a**–**f** *n* refers to individual mice. **g** Limiting dilution transplantation analysis for secondary wildtype recipient mice transplanted with sorted wildtype or CaSR KO AML cells (*n* = 4–5 mice per recipient group, Poisson statistics). **h**, **i** Colonies from the BM (**h**) or spleen (**i**) of wildtype mice transplanted with wildtype (black) or CaSR KO (blue) MLL-AF9-transduced BM (*n* = 4, two-tailed *t* test, mean ± SD). **j** Colonies from the serial replating of BM-derived cells from wildtype mice transplanted with MLL-AF9-transduced wildtype (black) or CaSR KO (blue) BM (*n* = 3, two-way ANOVA, Sidak’s test, mean ± SD). **k** Relative expression of stemness- and self-renewal-related genes in unsorted BM cells from mice transplanted with MLL-AF9-transduced wildtype, (black) CaSR KO (blue) and CaSR OE (red) leukaemia-initiating cells (*n* = 3 (CaSR KO, CaSR OE), *n* = 6 (wildtype), two-way ANOVA, Sidak’s test, mean ± SD). From **h**–**k** *n* refers to biological replicates. **l** Volcano plot summarising all differentially expressed genes in sorted MLL-AF9^+^ (GFP^+^) Lin^−^ cells in the BM of wildtype recipient mice transplanted with MLL-AF9-transduced wildtype BM co-transduced with empty vector- or CaSR OE-expressing retrovirus. Upregulated versus downregulated genes are represented in red and blue, respectively. Data are presented as mean values ± SD. Source data are provided as a Source Data file. The schematics in **a**–**g** and **j** were created with BioRender.com.

To identify the contribution of CaSR to leukaemia progression, we transplanted 5-fluorouracil (5-FU)-pre-treated (for AML and CML) or non-5-FU-pre-treated (for B-ALL) wildtype (Mx1-Cre-ER^***−/−***^ CaSR ^flox/flox^; WT) or *Casr* KO (Mx1-Cre-ER^−^^***/****+*^ CaSR ^flox/flox^) donor BM into WT mice (Mx1-Cre-ER^−/−^ CaSR ^flox/flox^) using the retroviral transduction/transplantation models of MLL-AF9-driven AML, BCR-ABL1-induced CML or BCR-ABL1^+^ B-ALL^14^. In AML, transplantation of MLL-AF9^+^ CaSR KO BM cells and administration of poly I:C on day 3 after transplantation (Supplementary Fig. 8a), when homing is completed^14,24^, led to a significant reduction in the number of leukocytes (Supplementary Fig. 8b) and the percentage of MLL-AF9^+^ (GFP^+^) Gr1^+^ myeloid cells, representative of tumour burden (Supplementary Fig. 8c) in PB of WT recipient mice. A reduction of MLL-AF9^+^ (GFP^+^) Gr1^+^ cells in BM (Supplementary Fig. 8d) and significant prolongation of survival (*P* = 0.0034, Fig. 3a) were also observed in recipients of *Casr* KO leukaemia-initiating cells (LIC). Spleen weights were significantly reduced (Supplementary Fig. 8e) in mice receiving CaSR-deficient LIC, and a significant, negative correlation between CaSR expression on PB leukocytes of mice with AML with day of death was observed (Supplementary Fig. 8f). Ruling out decreased engraftment of CaSR-deficient LIC^3^, we demonstrated no significant differences in the proportion of LKS (Supplementary Fig. 8g, j) or granulocyte-monocyte progenitor (GMP) cells (Supplementary Fig. 8k, l), which harbour the LSC fraction in this model^23^, in the BM or spleen of mice transplanted with MLL-AF9^+^ wildtype or CaSR KO LIC 12 days after transplantation. In addition, ablation of CaSR 12 days after transplantation, when the disease was established (Supplementary Fig. 8m) also led to a significant reduction of leukocytes (Supplementary Fig. 8n, o), the percentage of MLL-AF9^+^ Gr1^+^ cells in PB (Supplementary Fig. 8p) and significant survival prolongation of survival (Supplementary Fig. 8q, r).

In contrast, in CML CaSR-deficiency on LIC resulted in a trend towards increased leukocytes (Supplementary Fig. 9a), no differences in the percentage of BCR-ABL1^+^ CD11b^+^ myeloid cells in PB (Supplementary Fig. 9b), but the significant shortening of survival of recipient mice (*P* = 0.049, Fig. 3b). Consistently, we found a trend towards a positive correlation between CaSR expression on PB leukocytes of mice with CML and day of death (Supplementary Fig. 9c). Similar to CML, reduced expression of CaSR on LIC in B-ALL^22^ resulted in a significant increase in leukocytes (Supplementary Fig. 9d), the percentage of BCR-ABL1^+^ BP1^+^ pre-B cells (Supplementary Fig. 9e) and significantly shortened survival (Supplementary Fig. 9f).

Complementarily, overexpression (OE) of CaSR by co-transduction of LIC with CaSR- or empty vector-expressing retrovirus, led to an increased leukocyte count (Supplementary Fig. 10a, b) and percentage of MLL-AF9^+^ Gr1^+^ cells (Supplementary Fig. 10c), as well as shortened survival of WT recipients with AML (*P* = 0.0021, Fig. 3c). In sharp contrast, CaSR overexpression in CML LIC (Supplementary Fig. 10d) led to a trend towards a decrease of the leukocyte count (Supplementary Fig. 10e) and a reduced percentage of BCR-ABL1^+^ CD11b^+^ leukocytes in PB (Supplementary Fig. 10f), as well as significant survival prolongation (*P* = 0.0156, Fig. 3d). With a focus on AML due to the greater need for efficient therapies, we confirmed the role of CaSR for AML development by efficiently rescuing the disease in recipient mice by CaSR overexpression in CaSR KO MLL-AF9^+^ BM cells (*P* = 0.0237, Fig. 3e, Supplementary Fig. 10g, h).

Immunophenotyping (Supplementary Fig. 11a) of WT, CaSR KO or CaSR OE MLL-AF9^+^ Gr1^+^ myeloid cells^26^ revealed varying expression levels of Gr1, whereby the Gr1 low population morphologically correlated with a higher blast/neutrophil ratio compared to the Gr1 high population, consistent with an increased percentage of blasts and higher AML severity^27^ (Supplementary Fig. 11b). Consistently, MLL-AF9^+^ CaSR KO AML cells, exhibited a reduced percentage of Gr1 low cells (Supplementary Fig. 11c), while CaSR OE leukaemia cells were characterised by a higher percentage of this blast-enriched Gr1 low population (Supplementary Fig. 11d).

CaSR-deficient MLL-AF9^+^ cells in AML were characterised by a significant increase of cells in the G1 and a reduction of cells in the S phase of the cell cycle (Supplementary Fig. 11e), as well as increased apoptosis compared to WT counterparts (Supplementary Fig. 11f). Other features of CaSR KO MLL-AF9^+^ cells were decreased DNA damage (Supplementary Fig. 11g) and decreased levels of reactive oxygen species (ROS) (Supplementary Fig. 11h), when compared to WT, but no changes were observed in MLL-AF9^+^ Lin^−^ cells. No differences in these parameters were observed in the spleen (Supplementary Fig. 11i–k).

Testing whether the CaSR role was specific to MLL-AF9^+^ AML, we observed that transplantation of 5-fluorouracil-pre-treated CaSR KO BM cells, transduced with a retrovirus expressing the oncoprotein MN1 (Supplementary Fig. 12a), into WT recipients resulted in a lower tumour load (Supplementary Fig. 12b, c), a reduced percentage of MN1^+^ Gr1^+^ cells in PB (Supplementary Fig. 12d) and, similar to MLL-AF9^+^ AML, a significant survival prolongation compared to recipients of WT LIC (Supplementary Fig. 12e).

In summary, these findings indicate that CaSR plays a highly oncogene- and/or disease-specific, differential role in CML, B-ALL and AML affecting pathophysiological features in leukaemia cells. In AML, these effects are independent of homing or engraftment of LIC.

### CaSR regulates self-renewal of AML LSC

To test the impact of CaSR on AML LSC stemness and self-renewal properties, secondary transplantation was performed. Transplantation of CaSR-deficient sorted Lin^−^ MLL-AF9^+^ (GFP^+^) LIC from primary mice with established AML led to a reduction in leukocytes (Supplementary Fig. 13a, b), the percentage of MLL-AF9^+^ Gr1^+^ cells in PB of secondary recipient mice (Supplementary Fig. 13c) and to prolongation of survival compared to controls receiving WT LIC (*P* = 0.0306, Fig. 3f). Consistently, the frequency of LSC was reduced 6.5-fold by CaSR-deficiency, as determined by a limiting dilution assay^14^ (*P* = 0.0049, Fig. 3g and Supplementary Fig. 13d, e). Plating of CaSR KO BM (*P* = 0.0337, Fig. 3h) or spleen (*P* = 0.0444, Fig. 3i) cells from mice with established MLL-AF9^+^ AML in methylcellulose gave rise to significantly less colonies compared to controls, whereby similar results were observed after replating (Fig. 3j). Accordingly, CaSR-deficient myeloid progenitor cells, including GMP cells (Supplementary Fig. 13f), but not LKS cells (Supplementary Fig. 13g, h), were significantly reduced in primary recipients. Additionally, there was a trend towards reduced disease clonality measured as the number of proviral integration sites^14,28^ in mice transplanted with CaSR KO compared to wildtype AML LIC (Supplementary Fig. 13i, j), indicating less or less competent LIC.

A targeted gene expression profile indicated lower expression of genes involved in maintaining the stemness of *CaSR* KO AML LSC, such as *Meis1*, whereas the opposite was found in CaSR OE LSC compared to controls (Fig. 3k, Supplementary Table 3). Consistently, a genome-wide transcriptome analysis revealed overall 497 up- and 299 downregulated differentially expressed genes (DEG) between WT versus CaSR OE MLL-AF9^+^ Lin^−^ cells, in particular showing an upregulation of several homeobox (HOX) genes in CaSR-overexpressing LIC (Fig. 3l), known to increase self-renewal potential and promote AML development^29,30^. Non-redundant enrichment analysis of upregulated and downregulated DEG revealed significant enrichment of terms related to cell adhesion, migration and cell cycle in CaSR OE versus WT AML LIC (Supplementary Fig. 13k, l). In summary, these data suggest that CaSR affects the function of AML stem and progenitor cells, with CaSR overexpression leading to upregulation of stemness- and self-renewal-related genes in AML LSC.

### CaSR activates MAPK and Wnt-β-catenin

For further insight into the role of CaSR for the pathophysiology of human AML, we knocked out *CASR* in THP1 cells (MLL-AF9^+^) (Supplementary Fig. 5a–c, Supplementary Table 4). This led to reduced proliferation (Supplementary Fig. 14a), a significant increase in cells in the G1 phase and a trend towards a decrease of cells in the S phase of the cell cycle (Supplementary Fig. 14b), increased apoptosis (Supplementary Fig. 14c), possibly due to a downregulation of the anti-apoptotic gene BCL2 Like 1 (*BCL2L1*) (Bcl-XL) (Supplementary Fig. 14d) and decreased DNA damage (Supplementary Fig. 14e, f), similar to our results with murine MLL-AF9^+^ cells. Furthermore, KO of *CASR* in THP1 cells resulted in an increased ability to phagocytose bacterial bioparticles, possibly indicative of increased differentiation^31^, compared to control (Supplementary Fig. 14g). In contrast, THP1 cells overexpressing CaSR (Supplementary Fig. 5d–f) showed opposite results (Supplementary Fig. 15a–g, Supplementary Table 4) and, additionally, a lower percentage of CD11b^+^ myeloid cells (Supplementary Fig. 15h) and higher ROS production (Supplementary Fig. 15i).

**Fig. 4:**
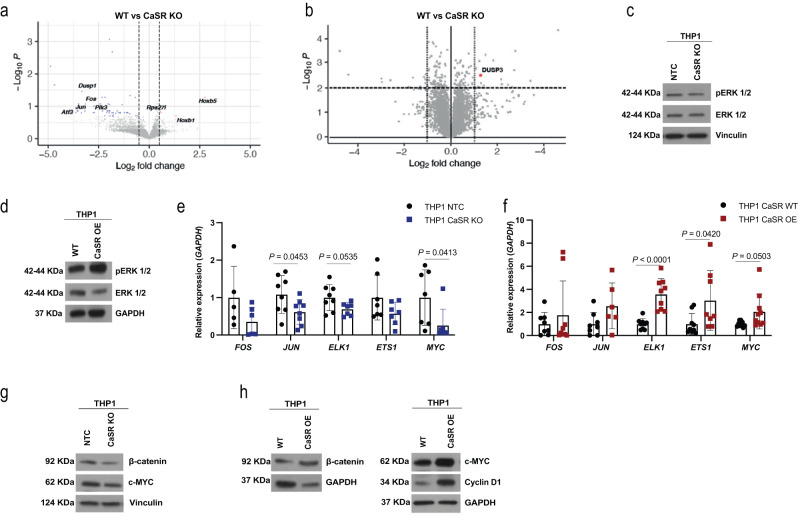
CaSR activates MAPK and Wnt-β-catenin. **a**, **b** Differentially expressed genes (**a**) and proteins (**b**) in sorted Lin^−^ MLL-AF9^+^ cells from the BM of WT (Mx1-Cre^−/−^ CaSR ^flox/flox^) mice transplanted with WT or CaSR KO (Mx1-Cre^±^ CaSR ^flox/flox^) MLL-AF9^+^ leukaemia-initiating cells. Each protein is represented by a dot, which is mapped according to its statistical significance (*P* value) versus magnitude of change (fold change). **c**, **d** Expression of ERK 1/2 and phospho ERK 1/2 (pERK1/2 (Thr 202/Tyr 204)) in lysates of THP1 non-target control (NTC) versus THP1 CaSR KO (**c**) and THP1 WT versus THP1 CaSR OE cells (**d**). The images are representative of three (**c**) or 6 (**d**) independent experiments. **e**, **f** Relative expression of pERK1/2 target genes in THP1 NTC versus THP1 CaSR KO cells (**e**) (*n* = 8, two-way ANOVA, Sidak’s test, mean ± SD) and THP1 WT versus THP1 CaSR OE cells (*n* = 9, two-way ANOVA, Sidak’s test, mean ± SD) (**f**). *n* refers to biological replicates. **g** Expression of β-catenin and c-MYC in lysates of THP1 WT and THP1 CaSR KO cells. The image is representative of eight independent experiments. **h** Expression of β-catenin (left), c-MYC and cyclin D1 (right) and glyceraldehyde 3-phosphate dehydrogenase (GAPDH) or vinculin, respectively, in lysates of THP1 WT versus THP1 CaSR OE cells. The image is representative of nine independent experiments. Source data are provided as a Source Data file.

To understand signalling pathways downstream of CaSR in AML LSC, we performed an RNA sequencing analysis of WT vs CaSR KO MLL-AF9^+^ Lin^−^ cells, which revealed 4 up- and 48 downregulated DEG and downregulation of downstream target genes in the MAPK/ERK pathway in CaSR KO compared to WT cells (Fig. 4a). Similarly, a proteomic analysis on Lin^−^ MLL-AF9^+^ cells uncovered significantly increased expression of dual specificity phosphatase 3 (DUSP3), a negative regulator of MAPK/ERK signalling^32^, in CaSR KO compared to WT cells (Fig. 4b). Consistently, pERK was decreased in *CaSR* KO (Fig. 4c, Supplementary Fig. 16a) and increased in CaSR OE (Fig. 4d, Supplementary Fig. 16b, Supplementary Table 5) THP1 cells. Supportive evidence of the involvement of this pathway was provided by decreased expression of transcription factors downstream of pERK1/2 in CaSR KO cells (Fig. 4e) and increased expression in CaSR OE (Fig. 4f) THP1 cells compared to the respective controls. Incubation of WT or CaSR OE THP1 cells in increasing concentrations of calcium did not significantly increase expression of pERK or ERK, but levels of the transcription factors *FOS* and *ETS1* versus *FOS* and *JUN* downstream of pERK increased in increasing concentrations of calcium in WT THP1 and CaSR OE THP1 cells, respectively (Supplementary Fig. 16c, d). In addition to higher CaSR expression in THP1 versus K562 cells (Fig. 2a), we observed higher pERK1/2 levels in THP1 cells, suggesting activation of this pathway in MLL-AF9^+^ versus BCR-ABL1^+^ cells already at baseline (Supplementary Fig. 16e, f). Consistent with published reports on CaSR-associated Wnt-β-catenin signalling^33^, we showed decreased β-catenin levels in *CASR* KO (Fig. 4g, Supplementary Fig. 17a) and opposite results in *CASR* OE (Fig. 4h, Supplementary Fig. 17b) THP1 cells, suggesting increased self-renewal and proliferation^34^ in the latter. Downstream targets in the β-catenin pathway, c-Myc and cyclin D1, were also affected (Fig. 4g, h, Supplementary Fig. 17c–e).

**Fig. 5:**
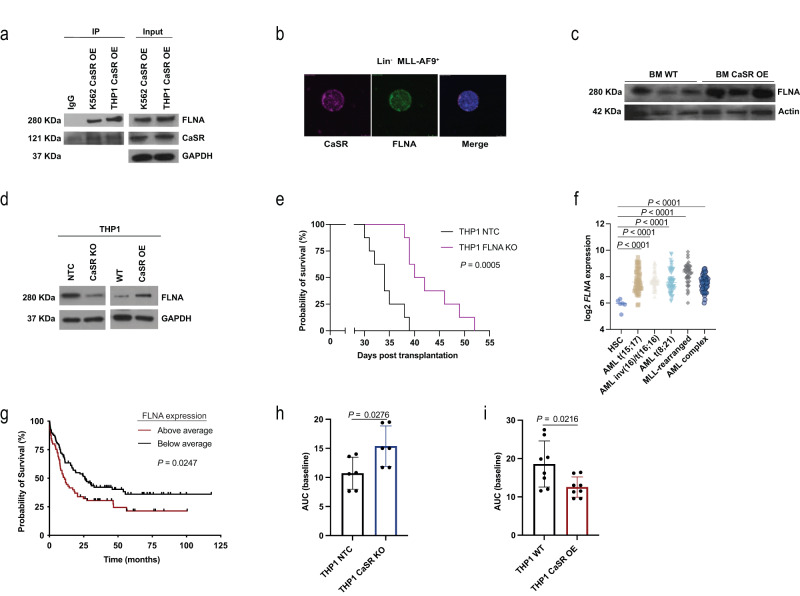
FLNA, downstream of CaSR, may play a role in AML progression. **a** Immunoblot for CaSR and FLNA in lysates of K562 and THP1 cells overexpressing CaSR, in which an anti-FLNA antibody was used to co-immunoprecipitate CaSR. The image is representative of 2 independent experiments. **b** Immunofluorescence staining for CaSR (purple) and FLNA (green) in sorted MLL-AF9^+^ (GFP^+^) Lin^−^ cells from WT mice with AML. The scale bar represents 5 μm. **c** Expression of FLNA in lysates of unsorted, total BM cells from individual WT mice transplanted with WT BM transduced with MLL-AF9 and co-transduced with empty vector- or CaSR-overexpressing retrovirus. Each column represents a single mouse. The image is representative of three biological replicates. **d** Expression of FLNA in lysates of non-target control (NTC), CaSR KO, WT and CaSR OE THP1 cells. The image is representative of 4 (NTC vs CaSR KO) and 7 (WT vs CaSR OE) independent experiments. **e** Kaplan–Meier style survival of NOD SCID interleukin (IL)-2 receptor γ KO (NSG) recipient mice transplanted with THP1 NTC (black) or THP1 FLNA KO (purple) cells (*n* = 8 (individual mice), Log-rank test). **f** Log2 expression of *FLNA* in sorted HSC from healthy individuals (*n* = 6) or unsorted BM cells from patients with AML t(15;17) (*n* = 54), AML inv(16)/t(16;16) (*n* = 47), AML t(8;21) (*n* = 60), MLL-rearranged AML (t(11q23)/MLL) (*n* = 43) or AML with complex karyotype (*n* = 46) (one-way ANOVA, Tukey test). **g** Kaplan–Meier style survival curve of patients with AML with above average (*n* = 96) or below average (*n* = 66) expression of *FLNA* (Log-rank test). From **f**, **g** *n* refers to individual patients. **h**, **i** Calcium flux represented by quantification of the area under the curve (AUC) at baseline in THP1 non-target control (NTC) (black) versus CaSR knockout (KO) (blue) cells (**h**) (*n* = 6, two-tailed *t* test) and THP1 WT (black) versus THP1 CaSR-overexpressing (OE) cells (red) **i** (*n* = 8, two-tailed *t* test). *n* represents biological replicates. Data are presented as mean values ± SD. Source data are provided as a Source Data file.

Despite not being significantly altered in our transcriptomic or proteomic analyses in Lin^−^ cells, filamin A (FLNA), a crosslinker of actin filaments and CaSR-interacting protein^35, 36^, whose deletion abrogates CaSR-associated MAPK signalling^37^, co-immunoprecipitated (Fig. 5a) and colocalized (Fig. 5b, Supplementary Fig. 17f, Supplementary Table 6) with CaSR in THP1 cells or MLL-AF9^+^ Lin^−^ cells, respectively. FLNA was increased in primary murine CaSR OE BM cells (Fig. 5c, Supplementary Fig. 17g) and up- versus downregulated in CaSR OE versus CaSR KO THP1 cells, respectively (Fig. 5d, Supplementary Fig. 17h–k). Knockout of *FLNA* from THP1 cells (Supplementary Fig. 18a–c) reduced their proliferative capacity (Supplementary Fig. 18d) and reduced cell cycling (Supplementary Fig. 18e) compared to control, but had no effect on apoptosis or DNA damage (Supplementary Fig. 18f, g). Strikingly, transplantation of THP1 *FLNA* KO cells into NOD SCID interleukin-2 receptor γ knockout (NSG) mice resulted in a reduction in human leukaemia cell frequency in PB (Supplementary Fig. 18h, i) and significant survival prolongation compared to mice receiving control cells (*P* = 0.0005, Fig. 5e). Mice transplanted with control THP1 cells died of hepatosplenomegaly due to likely infiltration of different organs by malignant cells, while mice receiving THP1 *FLNA* KO cells developed lymphadenopathy and, ultimately, paralysis, which may be due to the observed reduced migratory capacity of *FLNA* KO THP1 cells (Supplementary Fig. 18j). Depletion of *FLNA* in THP1 cells also resulted in reduction of MAPK/ERK activation and downstream target genes (Supplementary Fig. 18k, l).

Mining the Bloodspot database^38^ to test whether our murine findings may be transferrable to the human setting, we found significantly higher *CASR* (Supplementary Fig. 18m) and *FLNA* (*P* < 0.0001, Fig. 5f) expression in (MLL-rearranged) AML cells compared to normal HSC. Data from the TCGA database demonstrated significant prolongation of survival in patients, whose AML cells expressed below average levels of *FLNA* (*P* = 0.0247, Fig. 5g), but this was not significant for *CASR* expression (Supplementary Fig. 18n). Using human AML data from these databases we found a weak, but trending correlation between *CASR* and *FLNA* (Supplementary Fig. 18o), as well as between *FLNA* and *ELK1* and *FOS* (Supplementary Fig. 18p, q).

Calcium flux assays^39^ to test the intracellular calcium dynamics in CaSR-deficient or -overexpressing THP1 cells revealed an increase of intracellular calcium in CaSR-deficient (*P* = 0.0276, Fig. 5h) and a reduction of intracellular calcium in CaSR-overexpressing (*P* = 0.0216, Fig. 5i) THP1 cells at baseline. In THP1 OE cells, intracellular calcium levels also decreased in response to extracellular calcium and after ionomycin treatment to increase intracellular calcium levels (Supplementary Fig. 19a–c). In contrast, no such changes were observed in CaSR KO or CaSR OE K562 cells (Supplementary Fig. 19d, e). The changes in intracellular calcium levels in THP1 cells may be explained by CaSR-associated downregulation of the expression of several voltage-gated calcium channels (VGCCs) (Supplementary Fig. 19f). In sum, these results suggest that CaSR modulation may impact murine and human AML via MAPK and Wnt-β-catenin signalling. Our data implicate FLNA downstream of CaSR as possible regulator of MLL-AF9^+^ AML and suggest possible CaSR-mediated effects on intracellular calcium levels, which may contribute to our observations in MLL-AF9^+^ AML.

### Possible targeting of CaSR in AML

**Fig. 6:**
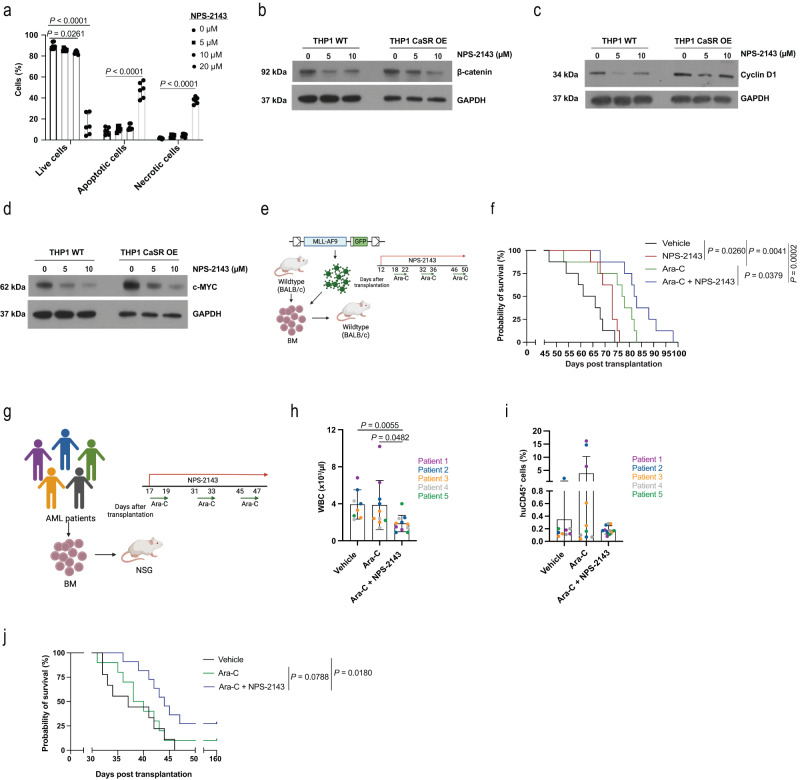
Possible targeting of CaSR in AML. **a** Percentage of WT THP1 cells of single cells, which are live, apoptotic or necrotic after treatment with the CaSR inhibitor NPS-2143 (*n* = 3 (biological replicates), two-way ANOVA, Dunnett’s test, mean ± SD). **b**–**d** Expression of β-catenin (**b**), cyclin D1 (**c**), and c-MYC (**d**) in WT or CaSR OE THP1 cells after treatment with NPS-2143. The image is representative of 7 independent experiments. **e**, **f** Schematic representation (**e**, (Created with BioRender.com)) and Kaplan–Meier style survival curve (**f**) of recipient WT BALB/c mice with MLL-AF9-induced AML treated with vehicle (black), NPS-2143 (red; 2 mg/kg, administered from day 12 to day 50 after transplantation as a daily regimen), ara-C (green; 50 mg/kg, administered on five consecutive days per cycle for three cycles every two weeks, starting on day 18 after transplantation) and the combination of both NPS-2143 and ara-C (blue) (as above) (*n* = 8, Log-rank test). *n* refers to individual mice. **g** Treatment scheme (Created with BioRender.com) for NOD SCID interleukin-2 receptor γ knockout (NSG) mice transplanted with BM cells from five individual patients with AML. Each sample was transplanted into 4–7 mice, whereby some mice were treated with vehicle, ara-C (50 mg/kg, administered for three consecutive days per cycle, for three cycles every two weeks) or the combination of ara-C (as above) and NPS-2143 (2 mg/kg, administered as a daily regimen). **h**, **i** White blood cell count (WBC) (**h**) and percentage of human CD45^+^ leukocytes (**i**) in the peripheral blood of NSG mice transplanted with human AML cells as in **g** and treated with vehicle (*n* = 9), ara-C (*n* = 10) or the combination of ara-C and NPS-2143 (*n* = 11), 30 days post transplantation. Recipients of the same human AML sample are indicated by the same colour (Kruskal–Willis, Dunn’s test, mean ± SD). **j** Kaplan–Meier style survival curve of NSG mice transplanted with human AML cells as in **g**–**i** and treated with vehicle (black), ara-C (green) or the combination of ara-C and NPS-2143 (blue) (*n* = 9 (vehicle), *n* = 10 (ara-C), *n* = 11 (NPS-2143), Log-rank test). From **h**–**j**, *n* refers to individual mice. Data are presented as mean values ± SD. Source data are provided as a Source Data file.

Probing the therapeutic relevance of our findings, we discovered that treatment of THP1 cells with NPS-2143^25^ efficiently induced apoptosis in THP1 cells (*P* < 0.0001, Fig. 6a). NPS-2143 treatment also significantly reduced protein levels of β-catenin (Fig. 6b), cyclin D1 (Fig. 6c) and c-Myc (Fig. 6d) in WT (Supplementary Fig. 20a) and CaSR OE THP1 cells (Supplementary Fig. 20b).

In vivo, in the syngeneic model of AML treatment of mice transplanted with wildtype MLL-AF9^+^ LIC with the combination of NPS-2143 and cytarabine (ara-C) (Fig. 6e), considered standard of care, once the disease was established (Supplementary Fig. 20c), resulted in a significant reduction of leukocytes (Supplementary Fig. 20d) and a trend towards a reduction of MLL-AF9^+^ Gr1^+^ myeloid cells (Supplementary Fig. 20e) in PB compared to mice treated with ara-C alone. Combination treatment also significantly prolonged survival compared to treatment with ara-C alone (*P* = 0.0379, Fig. 6f). In confirmation of these data, transplantation of human AML cells with varying genetic backgrounds (Supplementary Fig. 20f, Supplementary Table 7) into NSG mice treated with the combination of ara-C with NPS-2143 (Fig. 6g) significantly reduced leukocytes (Fig. 6h) and led to a non-significantly lower percentage of human CD45^+^ (Fig. 6i) or possibly a trend towards lower CD33^+^ cells (Supplementary Fig. 20g) in PB, when compared to mice treated with ara-C alone. A trend towards survival prolongation was found in those mice with AML which were treated by the combination therapy compared to ara-C alone (Fig. 6j, Supplementary Fig. 19h–l). Further, we observed a moderate, but significant correlation of CaSR expression with the percentage of human hCD45^+^ AML cells in the PB of the xenografted mice (Supplementary Fig. 20m), but CaSR expression on human AML cells did not correlate with response to treatment with NPS-2143 and ara-C by transplanted NSG mice (Supplementary Fig. 20n).

Lastly, in view of our findings on the tumour suppressor role of CaSR in CML (Fig. 3b, d), we tested the effect of the CaSR agonist and calcimimetic cinacalcet^40^ on CML development (Supplementary Fig. 21a). Combination of the tyrosine kinase inhibitor imatinib with cinacalcet led to a non-significant reduction of leukocytes (Supplementary Fig. 21b), BCR-ABL1^+^ CD11b^+^ myeloid cells (Supplementary Fig. 21c) and a significant survival prolongation compared to vehicle, but not imatinib-treated mice (Supplementary Fig. 21d). In conclusion, these data suggest that targeting of CaSR with NPS-2143 in AML in combination with standard chemotherapy may be superior to chemotherapy alone, though the numbers of tested human AML samples are small.

## Discussion

Our study expands on the previously discovered role of CaSR for HSC lodgement in the endosteal niche^3^, as well as on recent findings of HSC residing in areas with increased [eCa^2+^]^4^. We show a highly differential and unexpected role for CaSR in the progression of MLL-AF9^+^ (and MN1^+^) AML versus BCR-ABL1^+^ CML and B-ALL. CaSR expression levels impact the location of MLL-AF9^+^ AML cells in the BMM, as well as LSC function, possibly partly via alteration of calcium flux. We identified signalling pathways downstream of CaSR involving pERK, *β*-catenin and c-MYC, as well as filamin A for AML progression and LSC renewal, while providing a strategy for targeting CaSR or possibly FLNA in conjunction with standard chemotherapy. FLNA had previously only been implicated in AML as fusion partner in oncogenic chromosomal translocations^41^.

Bone remodelling results in the release of calcium ions (and other factors) from the bone matrix^6^, and proliferative HSC are predominantly found in actively remodelling BM cavities. In contrast, areas of bone with low resorption are largely devoid of expanding HSC^42^, underlining the theory that extracellular calcium from the BMM influences HSC and LSC physiology, as also put forth in our study. Consistently, in vitro culture of HSC in low calcium maintained properties of stemness^9^, suggesting that the decreased intracellular calcium levels we observed in CaSR-overexpressing THP1 cells may additionally promote characteristics of stemness in AML LSC. Contrastingly, in our study a higher extracellular calcium concentration increased CaSR expression, increased expression of pERK and some of its downstream transcription factors, supporting LSC features and suggesting that these discrepancies may be due to differences between HSC and LSC.

Leukaemias themselves remodel the BMM^15,43^, thereby possibly contributing to alterations of the endosteal calcium concentration and perpetuating the circuit identified by us. Indeed, bone formation seemed to be highest in murine AML compared to other leukaemias, in line with the notion that highly distinct interactions exist between the BMM and different types of leukaemia^14^. However, whether differences in BMM-remodelling by various leukaemias leads to altered levels of [eCa^2+^], impacting leukaemia progression, remains the object of future studies.

In vivo microscopy had shown that the organisation of haematopoietic stem and progenitor cells in the BMM of mice was nonrandom^44^, and LSC in imatinib-resistant CML were found closer to osteoblasts than non-resistant CML LSC^31^. While calcium was not studied in these reports, our findings may support the concept of non-coincidental location of haematopoietic cells in the BMM, which depends on BMM- or HSC/LSC-associated factors. Our data suggest that CaSR signalling is an important determinant of AML LSC stemness and leukaemia progression, but in how far niche location and [eCa^2+^] in the BMM may interplay or even synergise, will need to be determined. However, our xenotransplantation data do suggest that higher expression of CaSR correlates with increased engraftment of human AML cells, but not response to treatment with NPS-2143 and ara-C, whereby disease induction in immunocompromised mice is considered the hallmark of LSC^45^.

Our data reveal a correlation between CXCR4 expression on THP1 cells and the calcium concentration. However, we cannot exclude that integrins, with which CaSR forms complexes^46,47^, or other calcium-binding molecules like cadherins^48^ may be contributory to the observed phenotypes.

Furthermore, our results on the highly context-specific role of CaSR for leukaemia development is supported by published reports on the tumour suppressive versus oncogenic function of CaSR^11,13^. CaSR-associated differences between MLL-AF9^+^ and BCR-ABL1^+^ leukaemias in our study may be due to the observed higher levels of CaSR-pERK in MLL-AF9^+^ cells at baseline.

In an effort to improve current leukaemia therapies our observations may suggest that CaSR antagonism by calcilytics, which are being investigated for their anabolic effects in osteoporosis, hypocalcaemia or hypercalciuria^49^, may be beneficial as complementary treatment in AML. In contrast, calcimimetics, being used for treatment of secondary hyperparathyroidism^49^, may aid the eradication of LSC in CML or B-ALL. Also given our weak, but trending correlation between expression of *FLNA* and *CASR* or *FLNA* and certain transcription factors in human AML cells, therapeutic inhibition of FLNA raises hopes for innovative, adjunct therapies in AML. These may act via direct effects on FLNA downstream of CaSR and/or possibly via FLNA’s interaction with CXCR4^50^, which may prevent migration to chemotherapeutic niches.

## Methods

All research complied with relevant ethical regulations and approved by the local government (Regierungspräsidium Darmstadt, Germany) for murine studies and the ethics committees of the respective universities for human studies.

All authours complied with the guidelines on inclusion and ethics in global research.

### Mice

Mice conditionally lacking CaSR were generated by crossing Mx1-Cre mice with mice expressing mutant CaSR containing loxP sites flanking exon 5^51^. CaSR ^flox/flox^ mice were a kind gift from Prof. Martin Pollack and bred in the animal facility of the Georg-Speyer-Haus, Institute for Tumour Biology and Experimental Therapy.

To induce deletion of CaSR, mice (either primary mice used for testing the haematological phenotype or recipient mice transplanted with Mx1- *Cre-ER*^***−/−***^ CaSR ^flox/flox^ or Mx1- *Cre-ER*^***−/+***^ CaSR ^flox/flox^ BM), were intraperitoneally treated with 10 mg/kg of poly (I:C) (PHR1787, Sigma, Darmstadt, Germany). Poly (I:C) was administered 72 h after transplantation for four consecutive days. In some experiments poly (I:C) was administered every other day. For the transplantation experiment with late induction of the CaSR KO, poly (I:C) was given on four consecutive days starting on day 12 after transplantation, when short-term engraftment is thought to be completed. The efficiency of CaSR gene deletion was determined by flow cytometry analysis of leukocytes via an anti-CaSR antibody (NOVUS biologicals, Wiesbaden, Germany).

The calcium concentration was measured in the BM of Col1-caPPR mice (FVB background)^14,20^, which express constitutively active receptor for parathyroid hormone (PTH) and PTH-related peptide (PPR) specifically on osteoblasts (under the control of collagen type I α1 promotor) and are characterised by increased bone remodelling^14^. For xenotransplantation experiments, NOD SCID IL2Rγ KO (NSG) mice were obtained from The Jackson Laboratory (Bar Harbor, Maine, USA), housed and bred in specific pathogen-free (SPF) conditions. Balb/c mice were purchased from Charles River (Sulzfeld, Germany). Animals of different sexes were randomly assigned to experimental groups. All animal studies were performed in compliance with the guiding principles of the ‘Guide for the Care and Use of Laboratory Animals’^52^ and approved by the local government (Regierungspräsidium Darmstadt, Germany, protocol numbers F123/1047 and F123/2017). The maximally allowed tumour burden of 80% (oncogene^+^ Gr1^+^ cells) was not exceeded. All used mice were between the ages of 8–14 weeks. The mice were housed in individually ventilated cages at an ambient temperature between 20–26 degrees Celsius. A 14 hour light/10 h dark cycle was used.

### Genotyping of Mx1-**Cre**^**±**^ CaSR ^flox/flox^ mice

The genotyping was performed as described^51^. The DNA was extracted from ear biopsies by using MyTaq™ Extract-PCR Kit (Bioline, Luckenwalde, Germany) following the manufacturer’s instructions. The genomic DNA was subjected to PCR amplification to identify the mutations.

The presence of the CaSR flox allele was tested by performing a PCR amplification (2 min at 94 °C; 30 sec at 94 °C followed by 30 sec at 60 °C for 34 cycles and 1 min at 68 °C) using Casr-F and Casr-E3-R primers (see the table below). Zygosity was tested by digestion of the PCR product with the SalI enzyme (NEB Biolabs) for 3 h at 37 °C, which was then run on a 1% agarose gel. Homozygous mice generated 2 bands (400 bp and 250 bp), and heterozygous mice generated 3 bands (650 bp, 400 bp and 250 bp). The *Cre* transgene was detected by PCR using Cre FW and Cre RV primers (see the table below).

**Table:** 

Primer name	Primer sequence (3′→5′)
CaSR-F	5′-GACTTGCTATGTAGCCCAGAACTG-3′
CaSR-E3-R	5′-AAGGGATGTGCTCGGAGCA-3′
Cre Fw	5′-AAATTGCCAGGATCAGGGTTAAAG-3′
Cre Rv	5′-AGAGTCATCCTTAGCGCCGTAAAT-3′

### Bone marrow transduction/transplantation

#### Primary transplantation

The transplantation experiments were performed as previously described^14^. In brief, to induce chronic myeloid leukaemia (CML)-like myeloproliferative neoplasia (MPN) or acute myeloid leukaemia (AML), donor BM cells from 5-fluorouacil (5-FU)-pre-treated Mx1-Cre^−/−^ CaSR ^flox/flox^ or Mx1-Cre^−^^/+^ CaSR ^flox/flox^ mice (donors) (200 mg/kg; 4 days prior to harvest) were pre-stimulated overnight in medium containing stem cell factor (SCF) (50 ng/ml), IL-6 (10 ng/ml) and IL-3 (6 ng/ml) and transduced on two consecutive days with retrovirus. Subsequently, transduced cells were intravenously transplanted into sublethally irradiated (900 cGy for Mx1-Cre^−/−^ CaSR ^flox/flox^; 750 cGy for BALB/c in the overexpression experiments) WT recipient mice.

CaSR overexpression (OE) on LIC in CML and AML was achieved by co-transduction of donor cells from WT BALB/c mice with MSCV IRES RFP BCR-ABL1- or MSCV IRES RFP MLL-AF9-expressing retrovirus, respectively, and MSCV IRES GFP empty vector- or MSCV IRES GFP CaSR OE-expressing retrovirus. 2.5 × 10^5^ or 5 × 10^5^ cells to induce CML or AML, respectively, were transplanted in the overexpression experiments.

In a rescue experiment, BM cells from WT (Mx1-Cre^***−/−***^ CaSR ^flox/flox^) or inducible CaSR KO (Mx1-Cre^−^^***/+***^ CaSR ^flox/flox^) mice were co-transduced with MSCV IRES RFP MLL-AF9-expressing retrovirus and MSCV IRES GFP empty vector or MSCV IRES GFP CaSR OE-expressing retrovirus.

MN1-driven AML was induced via transduction of 5-FU-pre-treated BM cells from WT or inducible CaSR KO with pSF91-MN1 IRES eGFP-expressing retrovirus.

To induce AML or CML using the Mx1-Cre^***−/−***^ CaSR ^flox/flox^ or Mx1-Cre^−^^***/+***^ CaSR ^flox/flox^ mice 7.5 × 10^5^ or 4 × 10^5^ cells, respectively, were transplanted.

B-cell acute lymphoblastic leukaemia (B-ALL) was induced by retroviral transduction of non-5-FU pre-treated WT or CaSR KO BM cells with MSCV IRES GFP BCR-ABL1-expressing retrovirus and transplanted (1 × 10^6^ cells/mouse) into sublethally irradiated (900 cGy) WT mice.

#### Secondary and limiting dilution transplantation

For the secondary transplantation, BM from primary mice (Mx1-Cre^***−/−***^ CaSR ^flox/flox^ or Mx1-Cre^−/+^ CaSR ^flox/flox^) with established AML (sacrificed on day 40 after transplantation) was harvested. MLL-AF9^+^ (GFP^+^) Lin^−^ BM cells from primary mice were sorted on a BD FACS Aria Fusion Cell Sorter (Franklin Lakes, NJ, USA). The Lin^−^ cocktail included antibodies to CD11b, Ter119, CD5, B220 and F4/80. 1.5 × 10^4^ sorted MLL-AF9^+^ (GFP^+^) Lin^−^ cells were then combined with 2 × 10^6^ supportive BM cells from normal WT mice (Mx1-Cre^***−/−***^ CaSR^flox/flox^) and intravenously injected into sublethally irradiated (900 cGy) WT recipient mice (Mx1-Cre^***−/−***^ CaSR ^flox/flox^).

For the limiting dilution transplantation, 2.5 × 10^3^, 5 × 10^3^, 1 × 10^4^, or 1.5 × 10^4^ sorted MLL-AF9^+^ (GFP^+^) Lin^−^ BM cells from primary mice with AML were transplanted together with 2 × 10^6^ supportive BM cells (Mx1-Cre^***−/−***^ CaSR ^flox/flox^) into sublethally irradiated (900 cGy) WT (Mx1-Cre^***−/−***^ CaSR ^flox/flox^) mice. The frequency of stem cells was determined by Poisson statistics using the web-based ELDA (Extreme Limiting Dilution Analysis) statistical software^53^. Transplanted mice with a WBC < 8000/µl and MLL-AF9^+^ Gr1^+^ cells <20% in PB on day 24 after transplantation were considered as ‘non-engrafted’.

#### Xenotransplantation

For xenotransplantation of patient samples, bulk BM cells from five different AML patients (age range 57–76, both sexes) were intravenously transplanted into sublethally irradiated (125 cGy) NSG mice. Mice were randomly assigned to different treatment cohorts: vehicle, cytarabine (ara-C, PHR1787, Sigma, Darmstadt, Germany) or the combination of ara-C and NPS-2143 (Santa Cruz Biotechnology, sc-361280, Heidelberg, Germany).

For xenotransplantation of THP1 cells, 2 × 10^6^ cells were transplanted into non-irradiated recipient mice.

#### In vivo drug treatment

In the drug treatment experiments in the syngeneic mouse model, BALB/c mice with established AML were treated with intraperitoneal (i.p.) injection of vehicle (saline solution), 50 mg/kg ara-C (Sigma, Darmstadt, Germany), 2 mg/kg of NPS-2143 (Santa Cruz Biotechnology, Heidelberg, Germany) or a combination of ara-C with NPS-2143 (doses as above). Mice were randomly assigned to the different groups prior to treatment. NPS-2143 and vehicle were administered from day 12 to day 50 after transplantation as a daily regimen. Ara-C was administered on five consecutive days per cycle for three cycles every 2 weeks, starting on day 18 after transplantation.

In the xenotransplantation experiments, NSG mice were i.p. treated with vehicle (saline solution), ara-C (50 mg/kg) or the combination of ara-C and NPS-2143 (2 mg/kg). Treatment was performed from day 17, once human CD45^+^ cells were detected in PB, until day 47 after transplantation. NPS-2143 and vehicle were administered as a daily regimen and ara-C was given on three consecutive days per cycle, for three cycles every two weeks.

In the CML treatment experiment, BALB/c mice with established CML were randomly assigned to four cohorts: vehicle (water containing 0.01% DMSO), imatinib p.o. (100 mg/kg) (Enzo Life Sciences, ALX-270-492, Farmingdale, NY), cinacalcet p.o. (30 mg/kg, dissolved in 0.01% of DMSO in water) (Santa Cruz Biotechnology, sc-207438, Heidelberg, Germany) or a combination of imatinib and cinacalcet. Mice were treated by oral gavage on a daily basis from day 10 to day 26 after transplantation.

### Analysis of diseased mice and tumour burden

The leukaemia burden in the peripheral blood (PB) of diseased animals was assessed by analysis of peripheral blood using a Scil Vet animal blood counter (Scil Animal Care Company, Viernheim, Germany) and by flow cytometry analysis (BD Fortessa, Heidelberg, Germany) (Supplementary Table 2). The tumour burden was represented by the percentage of oncogene (GFP or RFP) positive leukocytes stained for CD11b (clone M1/70) antibody in the case of CML-like MPN, BP-1 (clone 6C3) for B-ALL and Gr1 (clone 1A8) for AML. Human CD45 was used to assess the tumour burden in xenotransplanted NSG mice. In the xenotransplantation experiments using patient samples, CD33 was additionally used. CaSR expression was tested in the different transplantation experiments by co-staining of malignant leukocytes with an anti-CaSR antibody (clone 5C10). All antibodies are listed in Supplementary Table 2.

### Calcium measurement

Quantification of the calcium concentration in the supernatant of human bone marrow aspirates, collected in heparin, was performed using a calorimetric calcium assay kit (ab102505, Abcam, Cambridge, UK) following the manufacturer’s instruction. The human samples were taken at diagnosis and the murine samples, when the mice were moribund and sacrificed.

In murine samples, bone marrow plasma was obtained by flushing a femur in 300 µl of PBS and collecting the supernatant by centrifugation (250 × *g* for 10 min). The same kit was used for the human samples.

K562 and THP1 cell culture media were collected for calcium release measurement using the same calorimetric calcium assay kit as used for the bone marrow supernatant. 5 × 10^3^ cells were seeded and culture media was collected over 7 days.

### ELISA

Levels of mouse PTH, PTHrP, PINP and CTX-I in the BM plasma of leukaemia mice were measured using MyBioSource ELISA kits (San Diego, CA, USA): Mouse Parathyroid Hormone (PTH) ELISA Kit (MBS704631), Enzyme-linked Immunosorbent Assay Kit for Parathyroid Hormone Related Protein (PTHrP) (MBS2086999), Enzyme-linked Immunosorbent Assay Kit for Procollagen I N-Terminal Propeptide (PINP) (MBS2021542) and Mouse crosslinked C-telopeptide of type I collagen (CTX-I) ELISA Kit (MBS703094), following the manufacturer’s instructions.

ELISA assays were performed using BM plasma obtained by flushing a femur of mice in 300 µl of PBS and collecting the supernatant by centrifugation (250 × *g* for 10 min).

### Leukaemia stem cells and myeloid progenitor analysis

Stem cells and myeloid progenitors present in BM, spleen and PB of normal mice or mice with AML were analysed by flow cytometry (BD Fortessa, Heidelberg, Germany). Cells were stained for the lineage markers CD11b, Ter119, CD5, B220 and F4/80, as well as c-Kit, Sca1, CD34 and FcγR (Supplementary Table 2). Lin^−^ c-Kit^+^ Sca-1^+^ (LKS) and LKS CD48^−^ CD150^+^ (LKS SLAM) cells identified haematopoietic stem cells, Lin^−^ IL7R^−^ c-Kit^+^ Sca-1^−^ CD16/32^+^ CD34^+^ identified granulocyte-monocyte progenitors (GMP), Lin^−^ IL7R^−^ c-Kit^+^ Sca-1^−^ CD16/32^−^ CD34^−^ identified megakaryocyte erythroid progenitors (MEP) and Lin^−^ IL7R^−^ c-Kit^+^ Sca-1^−^ CD16/32^−^ CD34^+^ identified common myeloid progenitors (CMP).

### Colony-formation assay

The colony-forming ability of MLL-AF9-transformed murine BM cells was examined using methylcellulose (MethoCult GF M3434, StemCell Technologies) according to the manufacturer’s instructions. 1 × 10^4^ total BM or spleen cells from individual AML mice (sacrificed on day 40 after transplantation) were plated in triplicate in methylcellulose medium (MethoCult GF M3434, StemCell Technologies). Plates were kept at 37 °C, and the number of colonies was determined 7 and 10 days after plating.

In serial replating experiments, 1 × 10^3^ sorted MLL-AF9^+^ GFP^+^ Lin^−^ cells from the BM of individual mice with AML (sacrificed on day 40 after transplantation) were plated in methylcellulose (MethoCult GF M3434, StemCell Technologies). After 7 days the cells were collected from the methylcellulose, counted and replated in fresh methylcellulose. The same number of cells were replated in the consecutive platings, which was repeated six more times.

### Giemsa staining

1 × 10^5^ sorted MLL-AF9^+^ Gr1^low^ and MLL-AF9^+^ Gr1^high^ BM cells were collected from mice with AML (sacrificed on day 40 after disease induction) and cytospun (50 × *g* for 3 min) onto slides. Cells were then fixed with methanol and stained with Giemsa stain according to standard protocols. The frequency of the leukaemic blasts versus differentiated cells was determined by a blinded pathologist (D.S.K.) based on the typical morphologies.

### Cell lines

THP1 and K562 cell lines were cultured in RPMI 1640 medium (Thermo Fisher Scientific, Darmstadt), supplemented with 10% fetal bovine serum (FBS), 2% L-glutamine and 1% penicillin/streptomycin. The human cell lines 293 T and HS-5 and the mouse cell line NIH/3T3 were cultured in DMEM medium supplemented with 10% FBS, 2% L-glutamine and 1% penicillin/streptomycin. The medium for 293 T and HS-5 cells was further supplemented with 1% non-essential amino acids. The murine pro-B cell line BA/F3 was grown in RPMI containing 10% FBS, 1% penicillin/streptomycin and 1% L-glutamine supplemented with 5% (v/v) WEHI media as a source of interleukin-3 (IL-3). All cell lines were maintained in a 37 °C, 5% CO_2_ incubator. All cell lines were obtained from DSMZ (German Collection of Microorganisms and Cell Cultures GmbH) or ATCC (American Type Culture Collection).

The cell lines used in this study were THP1 (ACC16), K562 (ACC10), Nalm-6 (ACC128), Kasumi (ACC220), 293 T (ACC635), NIH/3T3 (ACC59), BA/F3 (ACC300), HS-5 (CRL-3611).

### Production of retrovirus

Generation of retroviruses (MSCV IRES GFP (empty vector), MSCV IRES GFP BCR-ABL1, MSCV IRES RFP BCR-ABL1, MSCV IRES GFP MLL-AF9, MSCV IRES RFP MLL-AF9, pSF91-MN1 IRES eGFP and MSCV IRES GFP CASR was performed using the calcium phosphate transfection method^14^. Briefly, human HEK 293 T cells were co-transfected with the respective retroviral plasmid (10 µg/ 3 × 10^6^ cells) and the packaging plasmid EcoPak (5 µg/ 3 × 10^6^ cells)^14^. 48 h after transfection, the conditioned medium containing the viral particles was harvested and frozen. The viral titre was tested by transduction of NIH/3T3 cells and flow cytometric analysis.

### Intravital microscopy (IVM)

IVM experiments were performed as previously described^22,31^. To determine the in vivo localisation of human THP1 and K562 cells, the cells were labelled with 1 μm CMTMR dye (C2927, Invitrogen, Germany) and transplanted intravenously (5 × 10^5^ cells) into unirradiated NOD SCID interleukin-2 receptor γ (IL-2 R γ) knockout (KO) (NSG) mice. To test the effect of the CaSR-antagonist NPS-2143 on the cells’ localisation, cells were pre-treated with either vehicle (DMSO) or 5 µM NPS-2143 (Sigma, Darmstadt, Germany) for 4 h at 37 °C. In order to visualise the vessels, dextran-FITC (1 mg/mouse) (46945, Sigma, Taufkirchen, Germany) was intravenously injected into the mice, and imaging was performed 2 h after injection.

For indirect measurement of the concentration of calcium ions in the bone marrow (BM), unirradiated Rag-2^−/−^ IL2R γ^−/−^ CD47^−/−^ mice (to circumvent rejection of transplanted cells due to strain differences and to optimise the experiments for human cells) were intravenously injected with 5 × 10^5^ BA/F3 cells expressing the GCaMP6s construct cloned into the pSBbi-Pur sleeping beauty (SB) plasmid (Plasmid #60523, Addgene, Watertown, MA, USA). The cells were then visualised in the calvaria of live, anaesthetised mice by 2-photon microscopy. In stacks of 7−17 images of an individual BA/F3-GCaMP6s cell, the GFP intensity of the middle stack was measured as representative value with Image J software (version 1.53t) and correlated with the cell’s distance to the endosteum. For all IVM experiments, mice were anaesthetised with ketamine/xylazine, followed by removal of the scalp and visualisation of the calvarium under a custom-built confocal two-photon hybrid IVM (LaVision Biotech, Bielefeld) using a 40x objective.

### Generation of CASR overexpression (OE) and GCamP6s constructs

The human *CASR* gene, a 3234 bp fragment, was cloned into the retroviral MSCV IRES GFP vector (Addgene, 20672) via XhoI and ApaI restriction sites. *CASR* cDNA was kindly provided by Dr. Bryan K. Ward (University of Western Australia Centre for Medical Research). hCaSR.XhoI.F and hCaSR.R were used for gene amplification, and hCaSR.seq primers (1 to 5) were used for sequencing (Supplementary Table 1).

In order to overexpress CaSR in human cells, *CASR* was cloned into a SB construct via SfiI restriction sites. The SB constructs containing the reporter genes were kindly provided by Prof. Dr. Rolf Marschalek^54^. An internal SfiI site in the CaSR sequence was deleted by addition of a silent point mutation with a 2-step PCR approach (overlap extension PCR). Two initial PCRs with the primer combinations hCaSR.Sfi.F/hCaSR.int.R and hCaSR.Sfi.R/hCaSR.int.F were performed, whereby the internal primers covered the internal Sfi-site and introduced the silent point mutation to disrupt it. Subsequently, the two PCR reactions were pooled and used as template for a third PCR with the outer primers hCaSR.Sfi.F/hCaSR.Sfi.R. The amplimer was then gel-purified and cloned into a PCR-cloning vector (TopoTA-PCR2.1 Invitrogen) for sequencing. Primers hCaSR.SF1 to hCaSR.SF5 were used for sequencing. Error-free clones were digested with SfiI restriction enzyme and ligated into the SB vectors. Primer sequences are shown in Supplementary Table 1.

The cloning of the GCaMP6s sensor gene into a SB construct was performed via SfiI restriction sites. The gene sequence was amplified from pGP CMV GCaMP6s (Addgene, 40753) using the primer combination GCaMP6s.Sfi.F/GCaMP6s.Sfi.R. The amplimers were gel-purified and cloned into a PCR-cloning vector (TopoTA-PCR2.1 Invitrogen) for sequencing, using GCaMP6s.SF1 and GCaMP6s.SF2 primers (Supplementary Table 1). Subsequently, the GCaMP6s construct was cloned into the pSBbi-Pur plasmid (pSBbi-Pur, Plasmid #60523; Addgene, Watertown, MA, USA) constitutively expressing RFP.

THP1, K562 and BA/F3 cells overexpressing CaSR were generated by stable transfection with the SB constructs mentioned above. Plasmid transfections were performed using Metafectene Pro transfection reagent (Biontex Laboratories GmbH, Germany) according to the manufacturer’s instructions. Forty-eight hours after transfection, transformed cells were selected by puromycin (GIBCO, A11138) treatment (1 μg/ml).

### Generation of CRISPR/Cas9 KO constructs

The sgRNAs were designed to target the sequence of *FLNA* and *CASR*. All targets were chosen using the benchling design tool (https://benchling.com/crispr). The complementary DNA oligonucleotides containing selected CRISPR/Cas9 target sequences were generated by the annealing of two primers (Supplementary Table 4) and subsequently cloned into pLentiCRISPR.v2-GFP or pLentiCRISPR.v2-RFP (Addgene #52961) using BsmBI sites. sgRNA targeting CaSR were cloned into a backbone vector containing GFP as a reporter gene, and sgRNA targeting FLNA were cloned into a backbone vector-expressing RFP. A non-targeting (NTC) sgRNA was used as control and cloned into both vectors. The integration was confirmed by sequencing analysis using the LKO.1 primer shown in Supplementary Table 4.

The generation of lentiviruses containing the different sgRNA was performed following the protocol described^55^. The conditioned medium containing the lentiviruses was harvested 48 h after transfection and immediately used to transduce cells. The CRISPR/Cas9 KO cells were generated in two steps. First, THP1 cells were transduced with lentivirus containing Cas9 (lentiCas9-Blast, Addgene, #52962). After transduction, cells, which had integrated Cas9 in their genome, were selected using blasticidin (Invivogen, San Diego, CA, USA) (15 μg/ml). Subsequently, selected cells were transduced with lentivirus containing the sgRNA. The efficiency of transduction was checked by flow cytometry (BD Fortessa, Heidelberg, Germany), and cell populations were isolated by sorting of GFP^+^ or RFP^+^ cells (BD FACSAria Fusion Cell Sorter, Franklin Lakes, NJ, USA). The KO efficiency was tested by qPCR and Western blot analysis.

### Quantitative polymerase chain reaction (qPCR)

RNA isolation was performed by lysing cells using TRIzol (Life Technologies/Thermo Fisher Scientific, Darmstadt, Germany). Chloroform was added (20% (v/v) of Trizol) to allow RNA extraction. The RNA was purified using the RNAeasy Mini Kit from Qiagen (Cat. No 74104, Qiagen, Düsseldorf, Germany), according to the manufacturer’s instructions.

Complementary DNA (cDNA) synthesis was performed by reverse transcription (RT) of 250 ng–1 µg of isolated RNA using the ProtoScript First Strand cDNA Synthesis Kit (Cat. No. SE6300S, New England BioLabs, Frankfurt am Main, Germany), following the manufacturer’s instructions. The quantitative PCR (qPCR) was performed in triplicates by using Power SyBR Green (A25742, Thermo Fisher Scientific, Darmstadt) containing 10 µM forward and reverse primers using the StepOnePlus Real-Time PCR System (Applied Biosystems, Foster City, California) and the StepOne Software v2.3. Primers are listed in Supplementary Table 3. The relative gene expression was assessed using the delta-delta Ct (threshold cycle)-value measurement using glyceraldehyde 3- phosphate dehydrogenase (GAPDH) as housekeeping gene.

### Long distance inverse (LDI) PCR

Long distance inverse (LDI)-PCR was performed to determine disease clonality^28^. Genomic DNA was isolated from spleen of moribund mice using a DNeasy Blood & Tissue Kit (Cat. No 69506, Qiagen, Hilden, Germany), and 0.5 μg was digested using NcoI-high fidelity (HF) (New England BioLabs, Frankfurt am Main, Germany) and then religated. LDI-PCR was then performed using GFP-specific primers.

### Immunoblotting

Cultured cells and primary BM cells were lysed using RIPA buffer (50 mM Tris HCl pH 7.4, 150 mM NaCl, 1% Triton X-100, 1% Na DOC, 0.1% SDS, 1 mM EDTA), freshly supplemented with protease and phosphatase inhibitor cocktails (Sigma-Aldrich, Darmstadt, Germany). The lysates were incubated on ice for 1 h, ultra-centrifuged for 30 min at 250 × *g*, and the supernatant containing the proteins was collected. The protein concentrations were assessed using Protein Assay Dye Reagent Concentrate (Bradford, Bio-Rad, Hercules, CA, USA) and Pre-Diluted Protein Assay Standards: Bovine Serum Albumin (BSA) Set **(**Sigma, A2153, Darmstadt, Germany). Equal amounts of protein were mixed with Roti®-Load 1 dye and denatured and run on NuPAGE^TM^ 4−12% bis-Tris gels (Thermo Fisher Scientific, Darmstadt, Germany). Proteins were blotted on methanol-activated PVDF Transfer Membranes (Thermo Fisher Scientific, Darmstadt, Germany) using the wet transfer method in presence of 20% methanol. Subsequently, the membranes were blocked with 5% milk in 0.1% TBS-T for 1 h at room temperature and incubated with primary antibodies overnight at 4 °C. After incubation with secondary horseradish-peroxidase (HRP)-conjugated antibodies for 2 h, membranes were washed with 0.1% TBS-T and developed using X-ray films (Fujifilm, Dusseldorf, Germany). Band intensities were quantified using FIJI Image J software (version 1.53t). A list of all antibodies can be found in Supplementary Table 5.

### Protein coimmunoprecipitation (Co-IP)

For the coimmunoprecipitation experiments, K562 and THP1 cells were lysed in lysis buffer (50 mM Tris HCl pH 7.4, 150 mM NaCl, 10% glycerol, 0.5% NP-40), and protein was extracted. Equal amounts of protein per sample (500 μg) were incubated with 2 μg of antibody against FLNA (sc-17749, Santa Cruz, Heidelberg, Germany) for 4 h at 4 °C, followed by overnight incubation with magnetic beads (Dynabeads Protein G, Sigma-Aldrich, Munich, Germany) at 4 °C. The protein-bead complex was washed several times with ice cold RIPA buffer and eluted in 5× Laemmli buffer, heated to 95 °C for 5 min. The eluted proteins were separated by SDS page, followed by immunoblotting.

### Immunofluorescence (IF)

Immunofluorescence studies on primitive AML cells were performed by using sorted MLL-AF9^+^ GFP^+^ Lin^−^ cells (BD FACSAria Fusion Cell Sorter, Franklin Lakes, NJ, USA), isolated from the BM of mice with AML, sacrificed on day 40 after transplantation. 7 × 10^4^ primary AML cells or 2 × 10^5^ THP1 cells were cytospun onto glass slides (50 × *g* for 3 min), fixed with 4% paraformaldehyde (PFA) (Morphisto, Frankfurt, Germany) for 10 min and permeabilized with 0.2% Triton X-100 (Sigma, Darmstadt, Germany) for 5 min. The cells were then blocked with 2% BSA in PBS for 30 min at room temperature. To test protein co-localisation, cells were stained overnight at 4 °C with FLNA and CaSR antibodies (Supplementary Table 6) in a humidified chamber. Thereafter, cells were stained with fluorophore-labelled secondary antibodies (Supplementary Table 6) for 2 h. The nuclei were counterstained with 5 μg/ml 4′,6-diamidin-2-phenylindol (DAPI), and cells were mounted using fluoroshield mounting medium with DAPI (ab104139, Abcam, Cambridge, UK). Images were acquired using a confocal laser scanning microscope (CSLM) (Leica SP5, Wetzlar, Germany), and analysed with Image J (version 1.53t) software. In the case of γ H2A.X staining, foci were manually counted from the maximum projection view.

### In vitro treatment with CaCl_2_ or a CaSR antagonist

In order to test the effect of extracellular calcium on THP1 and K562 cells, calcium chloride was added to the cells in increasing concentrations (0.5, 1, 2.5, 5, 7.5 mM) prior to the adhesion and migration experiments. The cells were then incubated for 4 h at 37 °C.

To test the effect of the CaSR-antagonist NPS-2143 on THP1 cells, 1 × 10^6^ cells were treated with either vehicle (DMSO) or 5, 10 or 20 µM NPS-2143 (Sigma, Darmstadt, Germany) for 4 h at 37 °C.

### Proliferation assay

Cell proliferation was assessed by staining of the cells with CellTrace^TM^ Far Red Cell Stain (Carboxyfluorescein succinimidyl ester, CFSE; Invitrogen, Darmstadt, Germany). 8 × 10^4^ cells were plated in 1 ml of medium and incubated at 37 °C for 45 min. The stained cells were analysed by flow cytometry on a BD LSR Fortessa (Becton Dickinson, Heidelberg, Germany) using the FACSDiva Software, to create a baseline required for the analysis. After 72 h, the cells were measured by flow cytometry, and the analyses were performed in FlowJo using the ‘FlowJo Proliferation Tool’.

### Cell cycle assay

The cell cycle status of primary murine leukaemia cells was analysed using total BM or spleen cells, collected from mice with AML, sacrificed on day 40 after transplantation. The cell cycle status of different cell lines was analysed by using 5 × 10^5^ cells, which had been serum-starved for 16 h. Cells were fixed and permeabilized with BD Cytofix/Cytoperm^TM^, washed with Perm/Wash^TM^ solution (Cat. no.: 554722, 554723; BD Biosciences, San José, CA) and stained overnight at 4 °C with an antibody to Ki-67 (BioLegend, San Diego, CA, USA). On the following day, DAPI (0.2 μg/ml) (Merck, Darmstadt, Germany) was added to each sample, and after 30 min of incubation the cells were analysed by flow cytometry (BD Fortessa, Heidelberg, Germany).

### Apoptosis assay

The extent of apoptosis of primary BM or spleen cells or of the different cell lines was tested by the staining of 5 × 10^5^ cells with annexin V protein in annexin-binding buffer (V13246, Thermo Fisher Scientific, Schwerte, Germany). After 1 h at 4 °C cells were resuspended in DAPI solution (0.2 μg/ml) (Merck, Darmstadt, Germany) and immediately analysed using a flow cytometer (BD Fortessa, Heidelberg, Germany).

### Measurement of ROS

Intracellular ROS levels of either primary cells or cell lines were detected using the ROS assay kit (Thermo Fisher, Darmstadt, Germany). 5 × 10^5^ cells were incubated with the CellROX Deep Red oxidative stress reagent for 40 min at 37 °C and analysed by flow cytometry (BD Fortessa, Heidelberg, Germany).

### Measurement of DNA damage (γH2AX)

DNA damage was tested in 5 × 10^5^ primary total BM or spleen cells, collected from mice with AML, or in cell lines, which had been serum-starved for 16 h. Fixed and permeabilized cells (BD Cytofix/Cytoperm^TM^, Cat. no.: 554722, BD Biosciences, San José, CA) were stained overnight with an antibody to γ H2AX (Cell Signaling, Frankfurt am Main, Germany), followed by incubation with the secondary antibody Alexa Fluor 647 (Invitrogen, Darmstadt, Germany) for 2 h at 4 °C.

### BioParticle phagocytosis assay

Phagocytosis was measured by incorporation of bioparticles, as previously reported^31^. For this, 2 × 10^5^ cells were incubated with 1 μg of pHrodo Red E. coli BioParticles Conjugate (Thermo Fisher, Darmstadt, Germany) for 90 min at 37 °C, and phagocytosis was assessed by flow cytometry (BD Fortessa, Heidelberg, Germany).

### Adhesion assay

Adhesion of cells was tested as the capacity of cells to adhere to the extracellular matrix (ECM) protein fibronectin (FN) after they had been pre-treated in increasing concentrations of calcium (0–7.5 mM) for 4 h. The wells of a 48-well plate were coated using 2.5 µg/well FN (Thermo Fisher, Darmstadt, Germany) at 4 °C for 16 h in 20 mM TBS (pH 7.6), containing 1 mM CaCl_2_^56^. 2% bovine serum albumin (BSA) was used as control. Unbound fibronectin was removed, the wells were carefully washed with PBS and blocked with 2% BSA in IMDM (Iscove modified Dulbecco medium) for 30 min at room temperature. The wells were again carefully washed, and 7 × 10^4^ cells were added to the BSA- or FN-coated wells and incubated for 6 h at 37 °C. The non-adherent cells were removed by gently washing the wells, and the number of adherent cells in each condition was counted.

### Chemotaxis assay

The cell migration capacity of different cell lines, after they had been pre-treated in increasing concentrations of calcium (0–7.5 mM) for 4 h, was tested in a transwell assay (Sarstedt, Nümbrecht, Germany), in which the lower chamber contained CXCL12 (100 ng/ml)^57^. 1.5 × 10^5^ cells, serum-starved for 16 h, were added to the upper chamber of the transwell assay in full medium and allowed to migrate through a porous membrane (8 μm pore size) for 2 h towards the lower chamber containing CXCL12. The migrated cells were counted using a light microscope.

To test the migration of THP1 and K562 cells towards stromal cells, 1.5 × 10^5^ THP1 cells (serum-starved for 16 h) were used in the upper chamber of a transwell assay (Sarstedt, Nümbrecht, Germany) in RPMI medium supplemented with 10% FBS, 1% P/S and 1% L-glutamine. The cells were allowed to migrate through a membrane (8 μm) for 4 h towards a lower chamber, in which 3 × 10^4^ HS-5 cells had been plated one day prior to the start of the experiment. Migrated cells were quantified by flow cytometry.

### Calcium flux assay

Mobilisation of intracellular calcium was measured by using a flow cytometric assay^39^. Briefly, 1 × 10^6^ cells were resuspended in 1 ml of cell loading medium (CLM, RPMI containing 2% FBS and 25 mM HEPES (pH 7.4)). The cells were stained with eBioscience^TM^ Indo-1 AM (1 µM) (Thermo Fisher, 65-0856-39, Darmstadt, Germany) for 45 min at 37 °C. The cells were acquired by using the time parameter on the BD Fortessa (Heidelberg, Germany) and analysed for indo-violet (405 nm) and indo-blue (485 nm). A baseline calcium flux analysis was performed for 60 sec, followed by addition of CaCl_2_ (0–7.5 mM) and acquisition for 60 sec. Ionomycin (1 µg/ml, I24222, Thermo Fisher, Darmstadt, Germany) was added at the end and cells were acquired for an additional 60 sec. The analyses were performed in FlowJo using the ‘Kinetics’ tool and the area under the curve (AUC) of the ratio 405/485 nm over time, corresponding to the ratio of bound/unbound Indo-1 to calcium.

### RNA sequencing

Illumina sequencing libraries were prepared using the TruSeq Stranded mRNA Library Prep Kit (Illumina) according to the manufacturer’s protocol. Briefly, poly(A) + RNA was purified from a maximum of 500 ng of total RNA using oligo(dT) beads, fragmented to a median insert length of 155 bp and converted to cDNA. The ds cDNA fragments were then end-repaired, adenylated on the 3′ end, adapter ligated and amplified with 15 cycles of PCR. The libraries were quantified using Qubit ds DNA HS Assay kit (Life Technologies-Invitrogen) and validated on an Agilent 4200 TapeStation System (Agilent Technologies, Santa Clara, CA, USA).

Based on Qubit quantification and sizing analysis, multiplexed sequencing libraries were normalised, pooled and sequenced on a NovaSeq 6000 with a final concentration of 300 pM (spiked with 1% PhiX control).

### RNA sequencing analysis

Adapter sequences were trimmed from paired end RNA-seq reads using TrimGalore (https://github.com/FelixKrueger/TrimGalore). Quality-filtered reads were mapped using STAR^58^ against the mouse genome (GRCm39). Uniquely mapped reads were retained and read counts were quantified with HTSeq^59^. Differential expression analysis was carried out with these counts using Bioconductor package DESeq2^60^. Volcano plots were plotted using an R package EnhancedVolcano (https://github.com/kevinblighe/EnhancedVolcano).

RNA sequencing data are available at Gene Expression Omnibus (GEO) under accession number GSE208239.

### Mass spectrometry

Quantitative mass spectrometry was performed on sorted (BD FACSAria Fusion Cell Sorter) MLL-AF9^+^ Lin^−^ cells from the BM of WT or CaSR KO mice with AML, sacrificed on day 40 after transplantation.

#### Sample preparation for mass spectrometry

Cell pellets were dissolved in lysis buffer (2% SDS (sodium dodecyl sulphate), 10 mM TCEP (tris(2-carboxyethyl) phosphine), 40 mM CAA (chloroacetamide), 50 mM Tris pH 8.5), boiled for 10 min at 95 °C, followed by 2 min of sonication and another 5 min of boiling at 95 °C. Proteins were precipitated by methanol-chloroform-precipitation, and protein pellets were resuspended in 8 M urea, 50 mM Tris pH 8.2. The protein concentration was determined using the BCA (bicinchoninic acid) assay (23225, Thermo Fisher Scientific, Darmstadt, Germany). 40 µg of protein from each sample were diluted in 0.8 M urea using digestion buffer (50 mM Tris pH 8.2) and incubated with LysC (Wako Chemicals, Neuss, Germany) at a 1:50 (w/w) ratio and with trypsin (V5113, Promega, Madison, WI, USA) at a 1:100 (w/w) ratio overnight at 37 °C. Digests were acidified using trifluoroacetic acid (TFA) to 0.5 %, and peptides were purified using SepPak tC18 columns (WAT054955, Waters Corporation, Milford, MA, USA). Eluted peptides were dried, resuspended in tandem mass tag (TMT) labelling buffer (0.1 M EPPS pH 8.2, 20% acetonitrile), and the peptide concentration was determined by micro BCA (23235, Thermo Fisher Scientific, Darmstadt, Germany). 10 µg of peptides per sample were mixed with TMT10 reagents (90111, A37724, 90061, Thermo Fisher Scientific, Darmstadt, Germany) in a 1:2 (w/w) ratio (2 µg TMT reagent per 1 µg peptide). Reactions were incubated for one hour at RT and subsequently quenched by addition of hydroxylamine to a final concentration of 0.5% at RT for 15 min. After verification of the labelling efficiency (>99%) and mixing ratios by LC-MS analysis of a test pool comprising 1/20^th^ of each sample, all samples were pooled accordingly, desalted by SepPak, as stated above, and dried again.

#### High pH micro-flow fractionation

Peptides were fractionated using high-pH liquid-chromatography on a micro-flow high-performance liquid chromatographer (HPLC) (Dionex U3000 RSLC, Thermo Fisher Scientific, Darmstadt, Germany). 30 µg of pooled and purified TMT-labelled peptides, resuspended in Solvent A (5 mM ammonium-bicarbonate, 5% ACN (acetonitrile)), were separated on a C18 column (XSelect CSH, 1 mm × 150 mm, 3.5 µm particle size; Waters Corporation, Milford, MA, USA) using a multistep gradient from 3–60% Solvent B (100% ACN) over 65 min at a flow rate of 30 µl/min. Eluting peptides were collected every 43 s from minute 2 for 69 min into a total of 96 fractions, which were cross-concatenated into 16 fractions. Pooled fractions were evaporated to dryness and stored at 20 °C until mass spectrometry analysis, for which they were resuspended in LC-MS grade water containing 2% ACN and 0.1% TFA.

#### LC-MS Analysis

Tryptic peptides were analysed on an Orbitrap Lumos coupled to an easy nLC 1200 (Thermo Fisher Scientific, Darmstadt, Germany) using a 35 cm long, 75 µm ID fused-silica column packed in house with 1.9 µm C18 particles (Reprosil pur, Dr. Maisch, Entringen, Germany), and kept at 50 °C using an integrated column oven (Sonation, Biberach, Germany). A synchronous precursor selection (SPS) multi-notch MS3 method was used to minimise the ratio compression^61^. Assuming equal amounts in each fraction, 750 ng of peptides were eluted by a non-linear gradient from 4 to 32% ACN over 90 min, followed by a step-wise increase to 75% ACN in 6 min which was then held for another 9 min. Full scan MS spectra (350–1400 m/z) were acquired with a resolution of 120,000 at m/z 200, maximum injection time of 100 ms and automatic gain control (AGC) target value of 4 × 10^5^. The most intense precursors with a charge state between 2 and 6 per full scan were selected for fragmentation (Top Speed with a cycle time of 1.5 s) and isolated with a quadrupole isolation window of 0.7 Th. MS2 scans were performed in the ion trap (Turbo) using a maximum injection time of 50 ms, AGC target value of 1.5 × 10^4^ and fragmented using collision-induced dissociation (CID) with a normalised collision energy (NCE) of 35 %. SPS-MS3 scans for quantification were performed on the 10 most intense MS2 fragment ions with an isolation window of 0.7 Th (MS) and 2 m/z (MS2). Ions were fragmented using higher-energy C-trap dissociation HCD with an NCE of 65% and analysed in the Orbitrap with a resolution of 50,000 at m/z 200, scan range of 110–500 m/z, AGC target value of 1.5 × 10^5^ and a maximum injection time of 86 ms. Repeated sequencing of already acquired precursors was limited by setting a dynamic exclusion of 45 s and 7 ppm, and advanced peak determination was deactivated.

#### Processing of mass spectrometry data

Raw data were analysed with Proteome Discoverer 2.4 (Thermo Fisher Scientific, Darmstadt, Germany). Acquired MS2-spectra were searched against the mouse reference proteome (Taxonomy ID 10090) downloaded from UniProt (12-March-2020; One Sequence Per Gene, 21959 sequences) and a collection of common contaminants (244 entries from MaxQuant’s contaminants.fasta) using SequestHT, allowing a precursor mass tolerance of 7 ppm and a fragment mass tolerance of 0.5 Da after recalibration of mass errors using the Spectra RC-node applying default settings. In addition to standard dynamic (oxidation on methionines and acetylation/Met-loss or Met-loss+acetylation of protein N-termini) and static (Carbamidomethylation on cysteins) modifications, TMT-labelling of N-termini and lysines were set as static modifications. False discovery rates were controlled on the peptide-spectrum match (PSM) and protein level using Percolator (<1% FDR). Only PSMs with a signal-to-noise ratio above 10 and a co-isolation below 50% derived from unique peptides were used for protein quantification after normalisation of total intensity.

#### Statistical analysis of mass spectrometry data

Filtering and statistical analysis of the Proteome Discoverer output (Proteins table) were performed in Perseus (v 1.6.15.0). Only proteins quantified in all replicates in both experimental groups (WT and KO) were used for statistical analysis. Significant proteins were defined after a Student’s *t* test applying a *P* value cutoff (<0.01) and fold-change threshold (log2ratio larger than ≥1).

All mass spectrometry proteomics data have been deposited in the ProteomeXchange Consortium^62^ via the PRIDE partner repository^63^ with the dataset identifier PXD035937.

### Human samples

The use of BM samples from male and female patients with AML (Supplementary Table 7) for xenotransplantation experiments was approved by the medical ethics committee II of the Medical Faculty of the University of Heidelberg (Approval number 2013-509 N-MA).

The supernatants of centrifuged, heparinised bone marrow aspirates from patients with different haematological malignancies were provided by the biobank of the University Center for Tumoral Diseases in Frankfurt am Main. These experiments were approved by the Ethics Committee of the University Clinic of the Goethe University Frankfurt (Approval number 274/18 and SHN-5-2020). Informed consent was obtained.

### Analysis of the human datasets

#### Bloodspot

mRNA expression data (log_2_) for *CASR* and *FLNA* in bone marrow cells of AML patients (GSE13159), as well as in normal haematopoietic stem cells (GSE42519), were obtained from the BloodSpot database^38^. The data were obtained from the datasets 210577_at and 214752_x_at for *CASR* and *FLNA*, respectively. ANOVA analysis was used to determine statistical differences among the different groups.

#### TCGA data analysis

The Kaplan-Meier-style survival curves of patients with AML with high or low expression of *FLNA* and *CASR* were generated using the publicly available dataset TCGA, accessed through the cBioPortal portal (https://www.cbioportal.org) and analysed by Log-rank test. The groups were categorised by above and below average or median expression of *FLNA* or *CASR*, respectively.

Results from the in silico analysis, presented as linear correlation, were obtained from the TCGA database by using the online resource cBioPortal for Cancer Genomics. mRNA data of expression of *FLNA*, *CASR*, *ELK1* and *FOS* were imported from the online database, after which statistical analyses were performed.

### Drawing of schematics

All schematics were drawn using BioRender.com (2023).

### Statistical analysis

Statistical analysis was performed using GraphPad software (Prism 9.0). Each experiment was conducted independently at least three times. Shapiro-Wilks normality test was applied to all experiments to test, if the data followed a normal distribution. If the data were normally distributed, a two-tailed t-test was used, when the means of two groups were being compared. To analyse differences between the means of more than two groups, one-way (for one independent variable) or two-way (for more than one independent variable) ANOVA were applied. Depending on the sample size and the group comparison, Dunnett, Tukey or Sidak’s post hoc tests were used.

All presented biological replicates are the average of at least three technical replicates.

When the data did not follow a normal distribution, nonparametric tests were used. Two variables were compared using the unpaired, two-tailed Mann-Whitney test and more than two variables were compared by applying the Kruskal–Wallis test. Differences in survival were assessed by the Kaplan-Meier nonparametric Log-rank test. For the limiting dilution transplantation assay, Poisson statistics were used. Data are represented as mean ±SD (standard deviation), and the significance level was set at *P* < 0.05.

### Reporting summary

Further information on research design is available in the Nature Portfolio Reporting Summary linked to this article.

### Supplementary information

Supplementary Information

Reporting Summary

### Source data

Source Data

## Data Availability

RNA sequencing data are available at Gene Expression Omnibus (GEO) under accession numbers GSE208239. All mass spectrometry proteomics data have been deposited in the ProteomeXchange Consortium^62^ via the PRIDE partner repository^63^ [https://www.ebi.ac.uk/pride/] with the dataset identifier PXD035937 Data on the expression of genes in human AML samples were obtained from the publicly available dataset TCGA, accessed through the cBioPortal portal (https://www.cbioportal.org). Supplementary information is available for this paper. Correspondence and requests for materials should be addressed to the corresponding author. Reprints and permissions information is available at www.nature.com/reprints. Source data are provided with this paper.
