# Supplementary material for: Distinct and targetable role of calcium-sensing receptor in leukaemia
Source: Nat Commun. 2023 Oct 6;14:6242. doi: 10.1038/s41467-023-41770-0 (PMC10558580; doi:10.1038/s41467-023-41770-0)
Supplement: Supplementary file 1 — Supplementary Information [file 41467_2023_41770_MOESM1_ESM.pdf]

Supplementary Information

Distinct and targetable role of calcium sensing receptor in leukaemia

Raquel S. Pereira, Rahul Kumar, Alessia Cais, Lara Paulini, Alisa Kahler, Jimena Bravo, Valentina R. Minciacchi, Theresa Krack, Eric Kowarz, Costanza Zanetti, Parimala Sonika Godavarthy, Fabian Hoeller, Pablo Llavona, Tabea Stark, Georg Tascher, Daniel Nowak, Eshwar Meduri, Brian J. P. Huntly, Christian Münch, Francesco Pampaloni, Rolf Marschalek, Daniela S. Krause

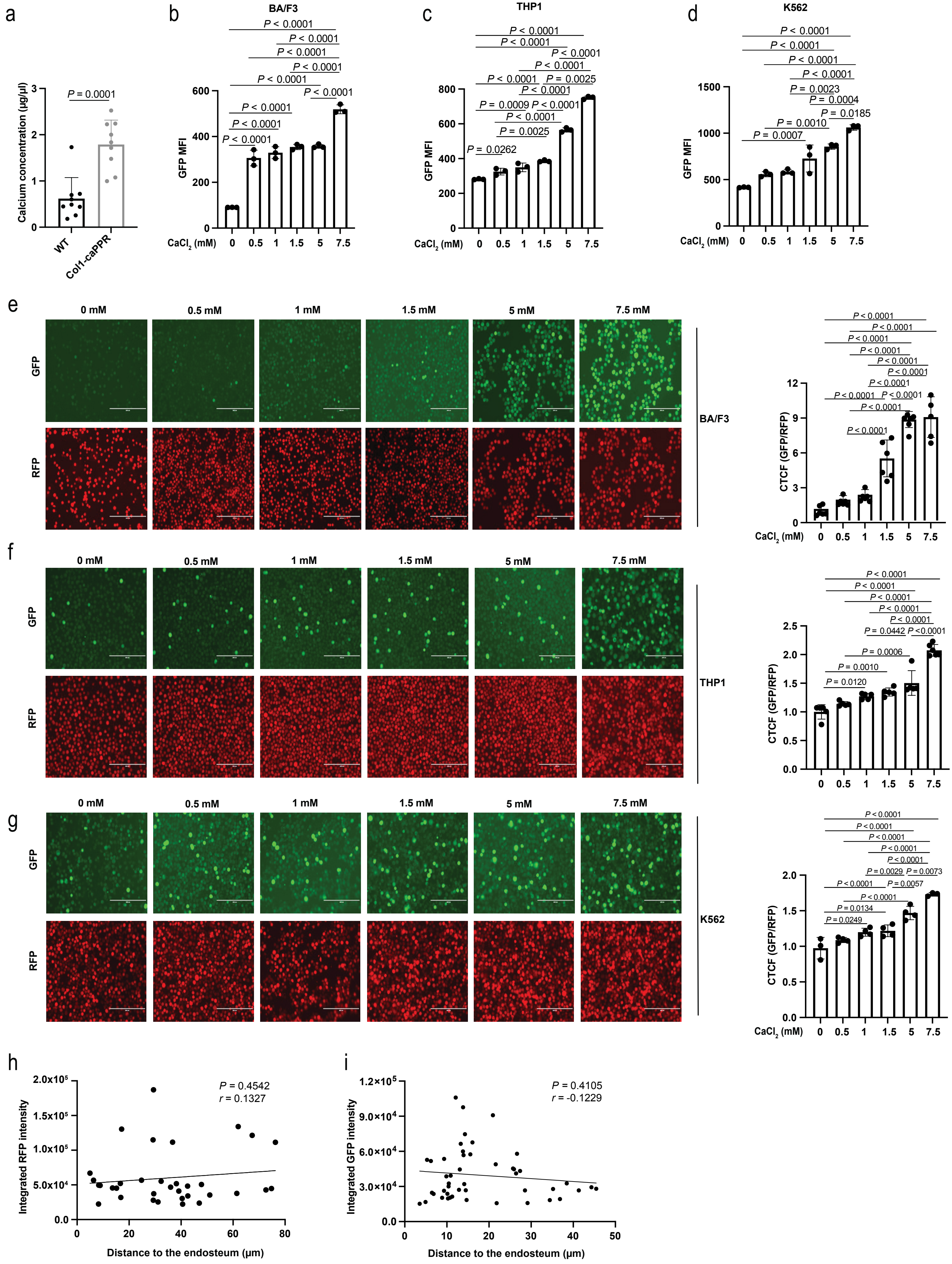

a
Calcium concentration (µg/µl)
P = 0.0001
WT
Col1-caPPR
b
BA/F3
GFP MFI
CaCl2 (mM)
0
0.5
1
1.5
5
7.5
P < 0.0001
c
THP1
P = 0.0025
P = 0.0009
P = 0.0262
d
K562
P = 0.0023
P = 0.0004
P = 0.0185
P = 0.0010
P = 0.0007
e
0 mM
0.5 mM
1 mM
1.5 mM
5 mM
7.5 mM
GFP
RFP
BA/F3
CTCF (GFP/RFP)
f
THP1
P = 0.0442
P = 0.0006
P = 0.0010
P = 0.0120
g
K562
P = 0.0029
P = 0.0073
P = 0.0057
P = 0.0134
P = 0.0249
h
Integrated RFP intensity
Distance to the endosteum (µm)
P = 0.4542
r = 0.1327
i
Integrated GFP intensity
P = 0.4105
r = -0.1229

Supplementary Figure 1. a) Quantification of the calcium concentration (μg/μl) in the BM of wildtype (WT) (FVB) mice and Col1-caPPR mice, which are characterized by an osteoblast-specific constitutive activation of the receptor for parathyroid hormone (PTH) and PTH-related peptide and increased bone remodeling. The BM of one femur per mouse was flushed and collected in 300 μl of PBS, and the calcium concentration present in the noncellular fraction was determined (n=3, two-tailed *t*-test). **b-d**) Median fluorescence intensity (MFI) of green fluorescence protein (GFP) in GCaMP6s$^+$ BA/F3 (**b**), THP1 (**c**) and K562 (**d**) cells after treatment with 0-7.5 mM of $CaCl_2$ for 5 minutes, measured by flow cytometry (n=3, one-way ANOVA, Tukey test). **e-g**) Immunofluorescence images (left) and quantification (right) of GCaMP6s$^+$ BA/F3 (**e**), THP1 (**f**) and K562 (**g**) cells expressing GFP and red fluorescence protein (RFP). Images were taken 5 minutes after the beginning of treatment with 0-7.5 mM $CaCl_2$ (n=5, one-way ANOVA, Tukey test). The scale bar represents 400 μm. **h)** Correlation of the integrated RFP intensity of BA/F3-GCaMP6s cells with the distance to the endosteum (μm), as measured by intravital microscopy. In stacks of 7-17 images of an individual BA/F3-GCaMP6s cell, the RFP intensity of the middle stack was used as representative value. Pearson's correlation coefficient (r) and the P-value are shown. The n represents 34 individual cells which were analyzed. The experiments were repeated three times. **i)** Correlation of the integrated GFP intensity of GFP$^+$ BA/F3 (MSCV IRES GFP BCR-ABL1$^+$ BA/F3) cells with the distance to the endosteum (μm), as measured by intravital microscopy to confirm lack of signal attenuation in calcium independence within the measured distance. In stacks of 3-7 images of an individual GFP$^+$ BA/F3 cell, the GFP intensity of the middle stack was used as representative value. Pearson's correlation coefficient (r) and the P-value are shown. The n represents 47 individual cells which were analyzed.

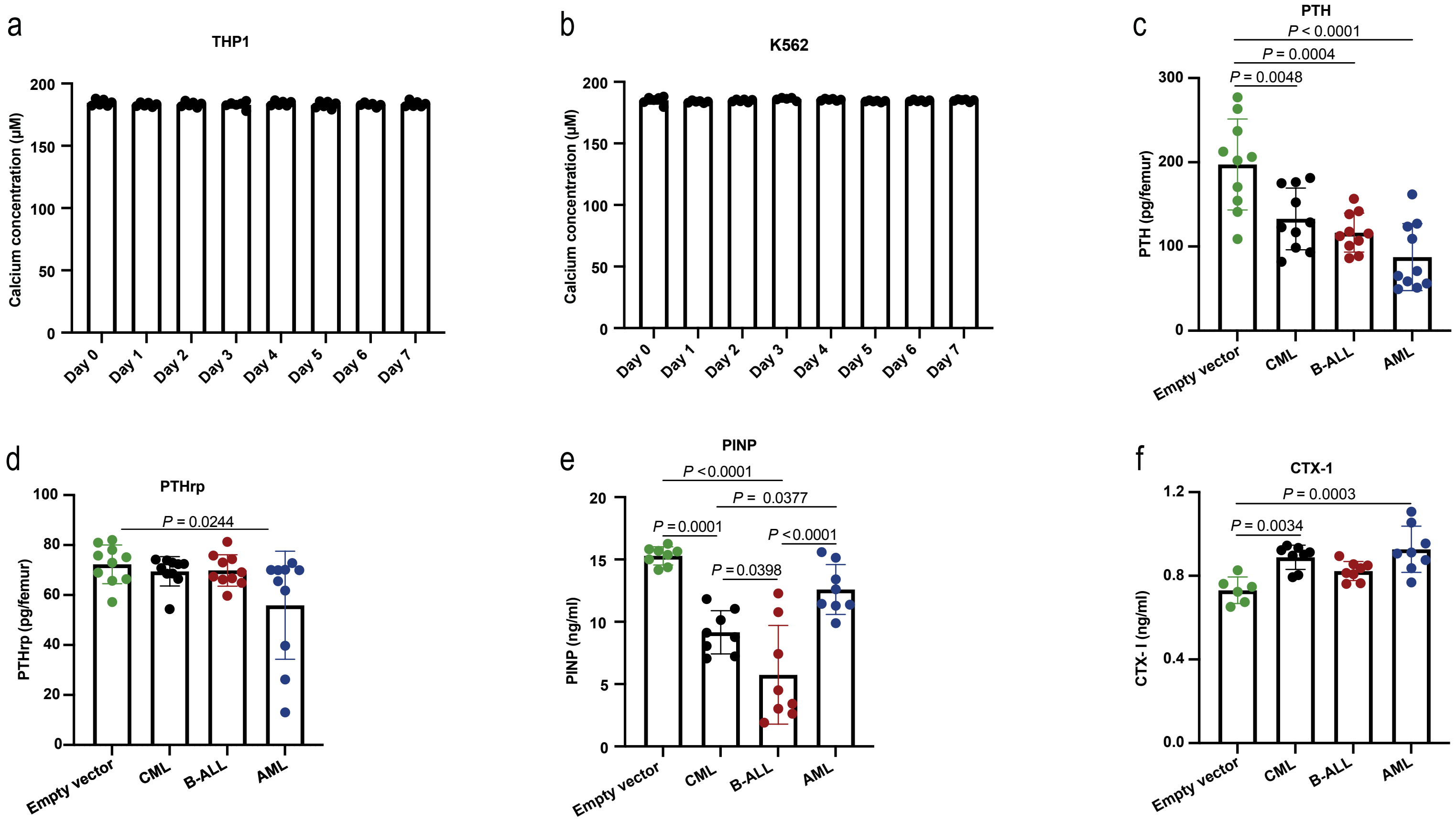

Supplementary Figure 2. a-b) Quantification of the calcium concentration (μM) in the cell culture media of THP1 (**a**) and K562 (**b**) cells (n=6, one-way ANOVA, Tukey test). **c-f**) Levels of parathyroid hormone (PTH, n=10) (**c**), parathyroid hormone-related peptide (PTHrp, n=10) (**d**), procollagen type I N-propeptide (PINP, n=8) (**e**) and C-terminal telopeptide of type I collagen (CTX-1, n=6-8) (**f**) present in the bone marrow (BM) of irradiated wildtype (Mx1-Cre$^{-/-}$ CaSR $^{flox/flox}$) mice transplanted with empty vector control-transduced BM or moribund mice with chronic myeloid leukaemia (CML), acute myeloid leukaemia (AML) or B-cell acute lymphoblastic leukaemia (B-ALL) shortly before death, as measured by enzyme-linked immunosorbent assay (ELISA). All diseases were induced with the retroviral transduction/transplantation model. The BM of one femur per mouse was flushed and collected in 300 µl of PBS, and the noncellular fraction was used for the ELISA (one-way ANOVA, Tukey test).

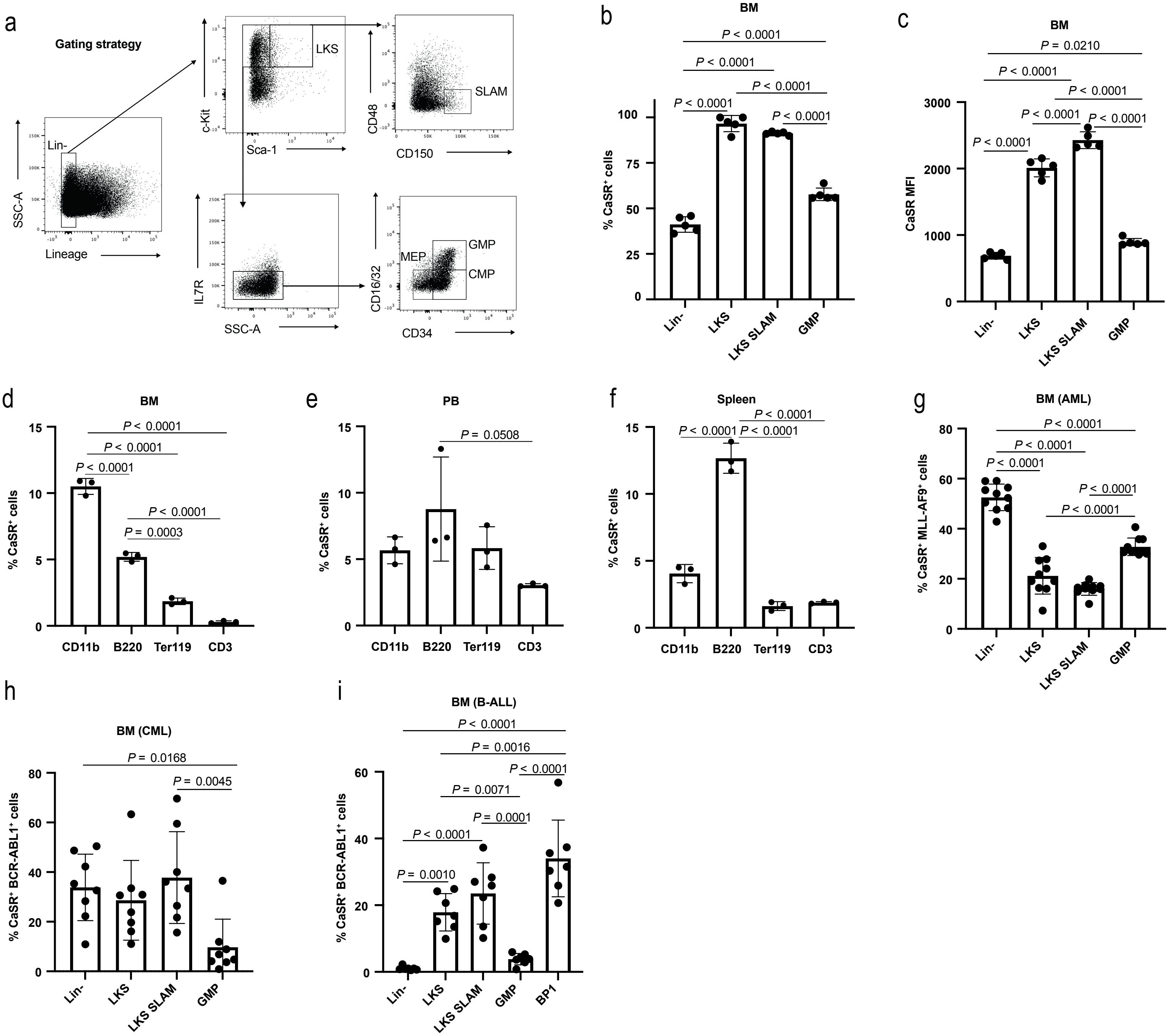

Supplementary Figure 3. a) Representative gating strategy for the analysis of Lin$^-$ c-Kit$^+$ Sca-1$^+$ (LKS), LKS CD48$^-$ CD150$^+$ (LKS SLAM) cells, Lin$^-$ IL7R$^-$ c-Kit$^+$ Sca-1$^-$ CD16/32$^+$ CD34$^+$ granulocyte-monocyte progenitors (GMP), Lin$^-$ IL7R$^-$ c-Kit$^+$ Sca-1$^-$ CD16/32$^-$ CD34$^+$ common myeloid progenitors (CMP) and Lin$^-$ IL7R$^-$ c Kit$^+$ Sca-1$^-$ CD16/32$^-$ CD34$^-$ megakaryocyte-erythroid progenitors (MEP). **b-c**) Percentage of CaSR$^+$ cells (**B**) and mean fluorescence intensity (**C**) of single cells in Lin$^-$, LKS, LKS SLAM and GMP cells of wildtype (Mx1-Cre$^{-/-}$ CaSR$^{flox/flox}$) mice (one-way ANOVA, Tukey test n=5). **d-f**) Percentage of CaSR$^+$ cells of single cells positive for CD11b (myeloid cells), B220 (B cells), Terr119 (erythrocytes), and CD3 (T cells) in the bone marrow (BM) (**d**), peripheral blood (PB) (**e**) and spleen (**f**) of wildtype mice (one-way ANOVA, Tukey test, n=3). **g-i**) Percentage of CaSR$^+$ cells of single cells in Lin$^-$, LKS, LKS SLAM and GMP cells of mice transplanted with wildtype (Mx1-Cre$^{-/-}$ CaSR$^{flox/flox}$) BM transduced with MLL-AF9- (**g**) or BCR-ABL1-expressing retrovirus in the CML (**h**) or B-ALL models (**i**) (one-way ANOVA, Tukey test, n=7-10).

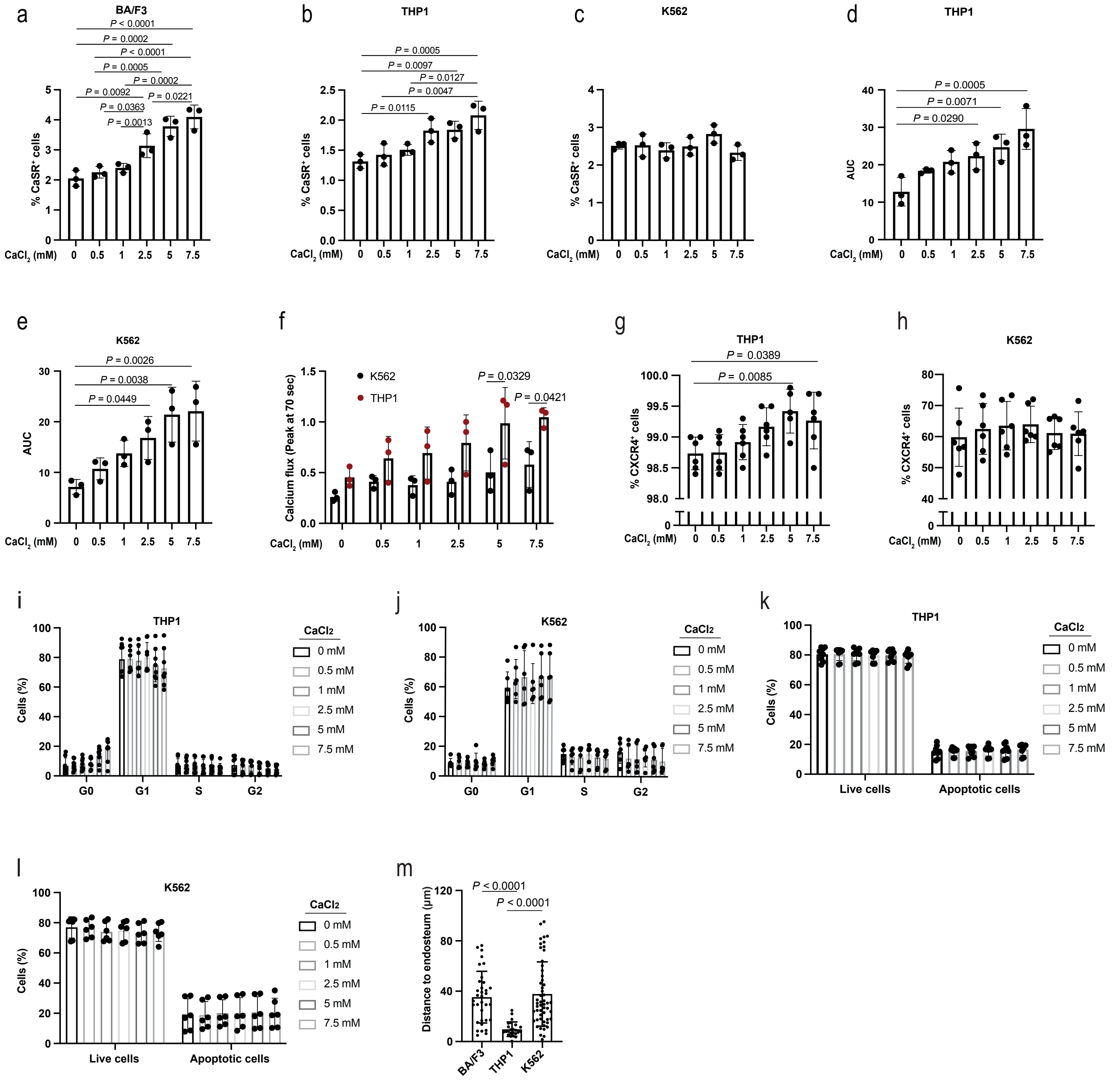

Supplementary Figure 4. a-c) Percentage of CaSR+ of GCaMP6s+ BA/F3 (n=3, one-way ANOVA, Dunnett test) (**a**), THP1 (n=3, one-way ANOVA, Tukey test) (**b**) and K562 (n=3, one-way ANOVA, Tukey test) (**c**) cells after treatment with 0-7.5 mM of $CaCl_2$ for 5 minutes, measured by flow cytometry. **d-e**) Quantification of the area under the curve (AUC) from the calcium flux analysis of THP1 (**d**) and K562 (**e**) cells, as represented in Figures 2f-g, respectively, using indo-1 dye after addition of 0-7.5 mM of $CaCl_2$ (n=3, one-way ANOVA, Dunnett test). **f**) Quantification of the calcium flux peak (ratio bound/unbound) of K562 and THP1 cells 70 seconds after $CaCl_2$ addition (0-7.5 mM) (n=3, two-way ANOVA, Sidak´s test). **g-h**) Percentage of CXCR4+ THP1 (**g**) and K562 (**h**) cells of single cells after treatment with different concentrations of $CaCl_2$ (0-7.5 mM) for 4 h (n=6, one-way ANOVA, Tukey test). **i-j**) Cell cycle status of THP1 (**i**) (n=8) and K562 (**j**) (n=6) cells of single cells treated with different concentrations of $CaCl_2$ (0-7.5 mM) for 4 h. **k-l**) Percentage of live or apoptotic THP1 (**k**) and K562 (**l**) cells of single cells treated with different concentrations of $CaCl_2$ (0-7.5 mM) for 4 h, detected by annexin V and DAPI staining (n=8). **m**) Distance (in µm) of intravenously transplanted CMTMR-labelled BA/F3, THP1 and K562 cells to the endosteum in the calvarium of Rag-$2^{-/-}$ IL2R $\gamma^{-/-}$ $CD47^{-/-}$ (BA/F3) or NOD SCID interleukin-2 receptor γ (IL-2Rγ)$^{-/-}$ (NSG) mice (THP1 and K562). $5x10^5$ cells had been transplanted and were imaged by intravital microscopy 2 h after transplantation (n=24-53, one-way ANOVA, Tukey test). The imaging analysis was performed by ImageJ. The experiments were performed in three different mice per cohort.

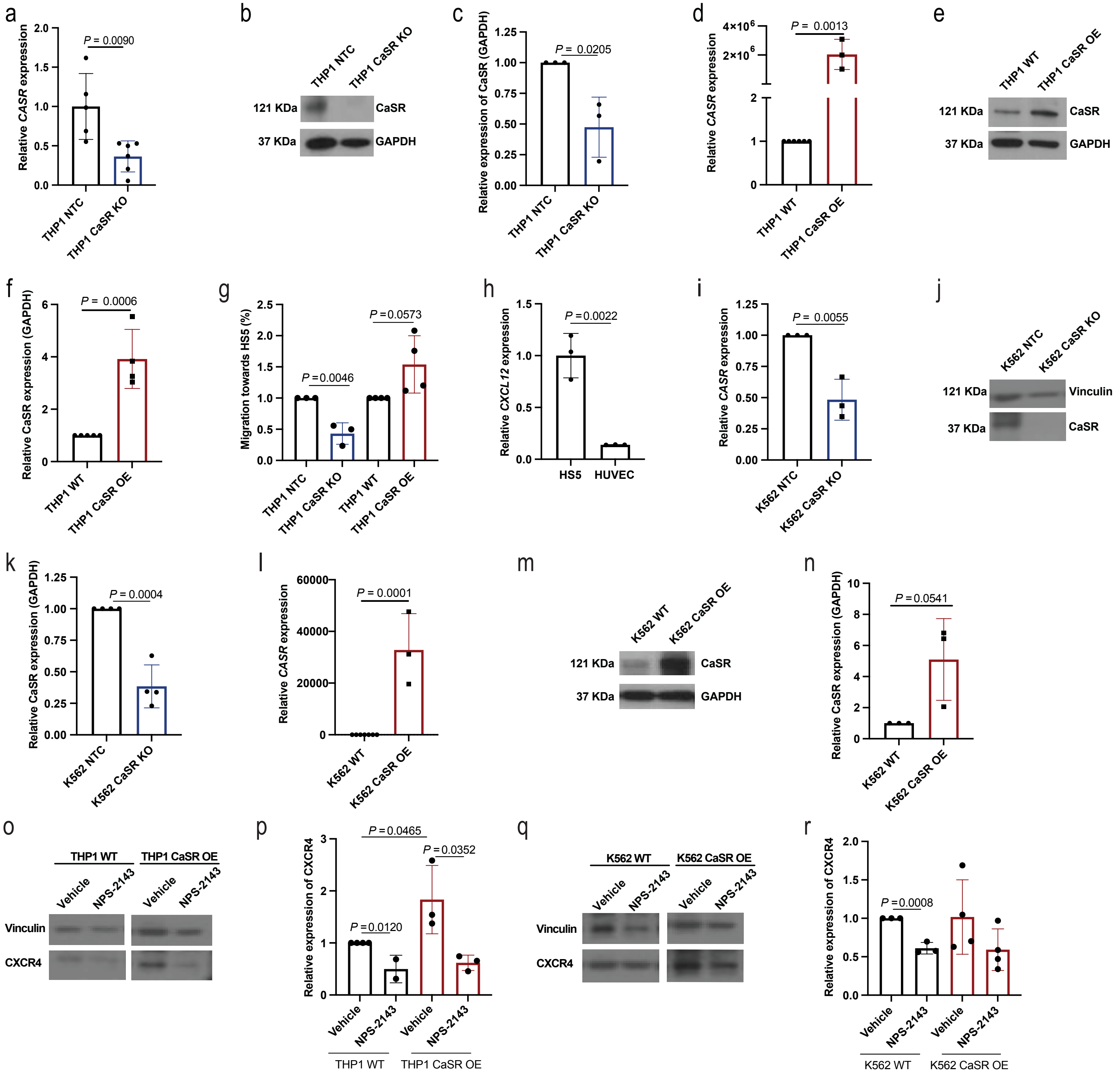

Supplementary Figure 5. a) Relative expression of *CASR* in non-target control (NTC) (black) or CaSR knockout (KO) THP1 cells (blue) by RT-qPCR analysis. Glyceraldehyde 3-phosphate dehydrogenase (*GAPDH*) was used as a housekeeping control (n=5-6, two-tailed *t*-test). **b-c**) Representative immunoblot analysis (**b**) and its quantification (**c**) of CaSR (121 kDa) expression relative to GAPDH (37 kDa) in total cell lysates of NTC or CaSR KO THP1 cells (n=3, two-tailed *t*-test). The blot is representative of three independent experiments. **d**) Relative expression of *CASR* in wildtype (WT) (black) THP1 cells or THP1 cells overexpressing CaSR (THP1 CaSR OE) (red) by RT-qPCR analysis (n=3, two-tailed *t*-test). **e-f**) Representative immunoblot analysis (**e**) and its quantification (**f**) of CaSR (121 kDa) expression relative to GAPDH (37kDa) in total cell lysates of WT THP1 or THP1 CaSR OE cells (n=3, two-tailed *t*-test). **g**) Number of THP1 NTC, CaSR KO, WT and CaSR OE cells which had migrated towards HS5 cells within 2 h. (n=3-4, one-way ANOVA, Tukey test). **h**) Relative expression of *CXCL12*, determined by RT-qPCR in HS5 and HUVEC cells (n=3, two-tailed t-test). Glyceraldehyde 3-phosphate dehydrogenase (*GAPDH*) was used as housekeeping gene. **i**) Relative expression of *CASR* in non-target control (NTC) (black) or CaSR knockout (KO) K562 cells (blue) by RT-qPCR analysis. Glyceraldehyde 3-phosphate dehydrogenase (*GAPDH*) was used as a housekeeping control (n=5-6, two-tailed *t*-test). **j-k**) Representative immunoblot analysis (**j**) and quantification (**k**) of CaSR (121 kDa) expression relative to GAPDH (37 kDa) in total cell lysates of NTC or CaSR KO K562 cells. The blot is representative of three independent experiments. **l**) Relative expression of *CASR* in WT (black) K562 cells or K562 cells overexpressing CaSR (K562 CaSR OE) (red) by RT-qPCR analysis (n=3, two-tailed *t*-test). **m-n**) Representative immunoblot analysis (**m**) and its quantification (**n**) of CaSR (121 kDa) expression relative to GAPDH (37kDa) in total cell lysates of WT THP1 or THP1 CaSR OE cells (n=3, two-tailed *t*-test) **o-p**) Representative immunoblot (**o**, **q**) and its quantification (**p**, **r**) for CXCR4 (40 kDa) and Vinculin (124 kDa) expression in lysates of THP1 (**o-p**) and K562 (**q-r**) cells treated with NPS-2143 for 4 h (n=3-4, one-way ANOVA, Tukey test). The blots are representative of three or four independent experiments.

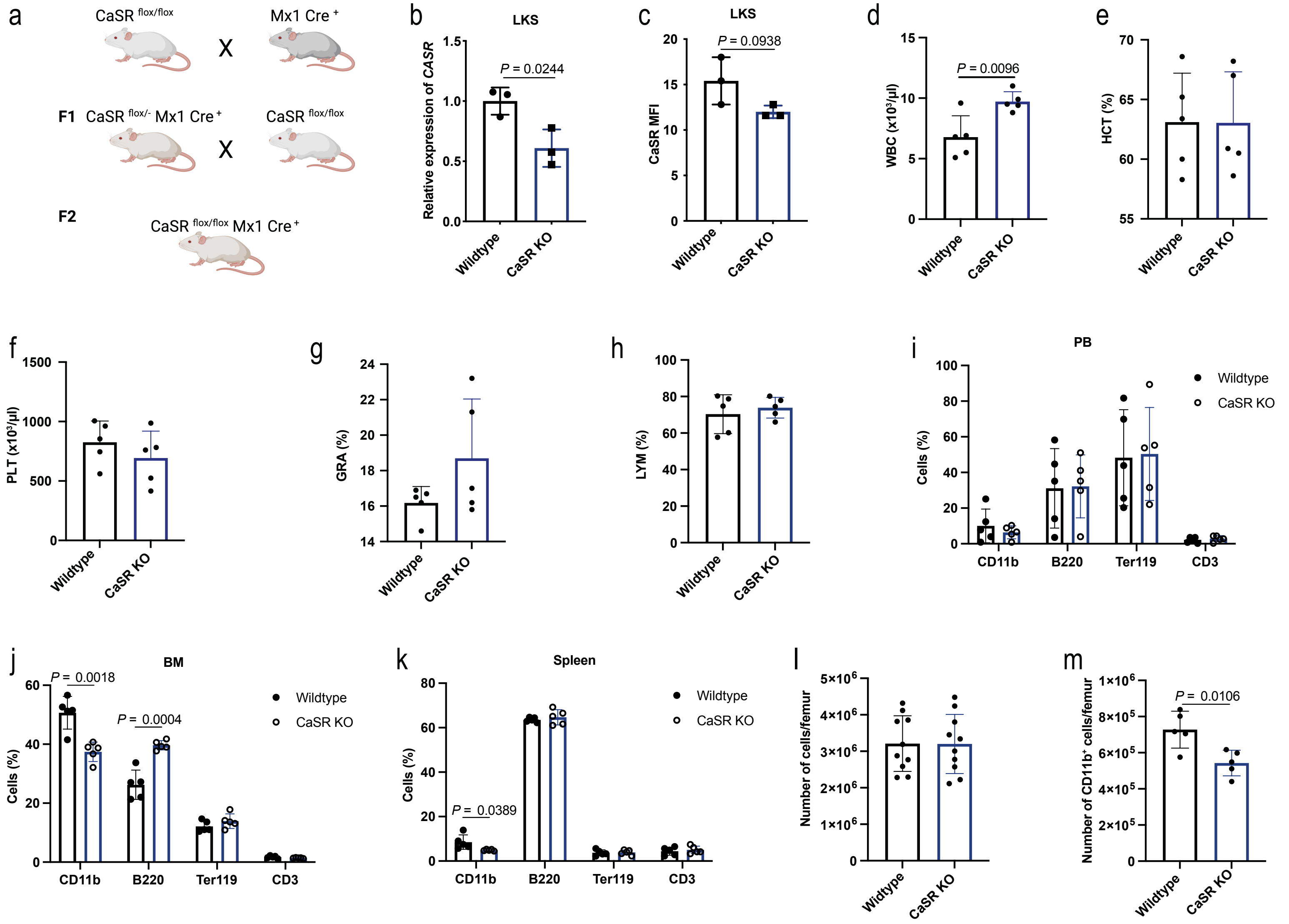

Supplementary Figure 6. a) Mating scheme for the generation of inducible CaSR KO mice. Mice homozygous for floxed *CaSR* alleles were crossed to animals heterozygous for the *Mx1-Cre* transgene to generate Mx1-Cre$^{-/+}$ CaSR$^{flox/-}$ mice. These mice were then crossed with CaSR$^{flox/flox}$ mice to generate litters which are homozygous for flox alleles (Mx1-Cre$^{-/+}$ CaSR$^{flox/flox}$). **b-c**) Relative expression and median fluorescence intensity (MFI), respectively, of *CaSR* determined by RT- qPCR (**b**) and by flow cytometry (**c**) on BM-derived Lin$^-$ c-Kit$^+$ Sca-1$^+$ (LKS) cells of wildtype (Mx1-Cre$^{-/-}$ CaSR$^{flox/flox}$) and CaSR KO (Mx1-Cre$^{-/+}$ CaSR$^{flox/flox}$) mice 10 days after poly I:C treatment (n=3, two-tailed *t*-test). **d-h**) White blood cell (WBC) count per *µ*l (**d**), haematocrit (HCT) (**e**), platelet (PLT) count per *µ*l (**f**), percentage of granulocytes (GRA) (**g**) and percentage of lymphocytes (LYM) (**h**) in peripheral blood (PB) of wildtype (black) or CaSR KO (blue) mice, as measured by complete blood count (CBC) analyser 10 days after poly I:C treatment (n=5, two-tailed *t*-test). **i-k**) Percentage of cells of single cells positive for CD11b (myeloid cells), B220 (B cells), Terr119 (erythrocytes), and CD3 (T cells) in the peripheral blood (**i**), bone marrow (**j**) and spleen (**k**) of wildtype (black) or CaSR KO (blue) mice 10 days after poly I:C treatment (two-way ANOVA, Sidak´s test n=4-5). **l-m**) Number of total BM cells (**l**) and CD11b$^+$ cells (**m**) per femur of wildtype and CaSR KO mice, 10 days after poly I:C treatment (n=10, n=5, two-tailed *t*-test).

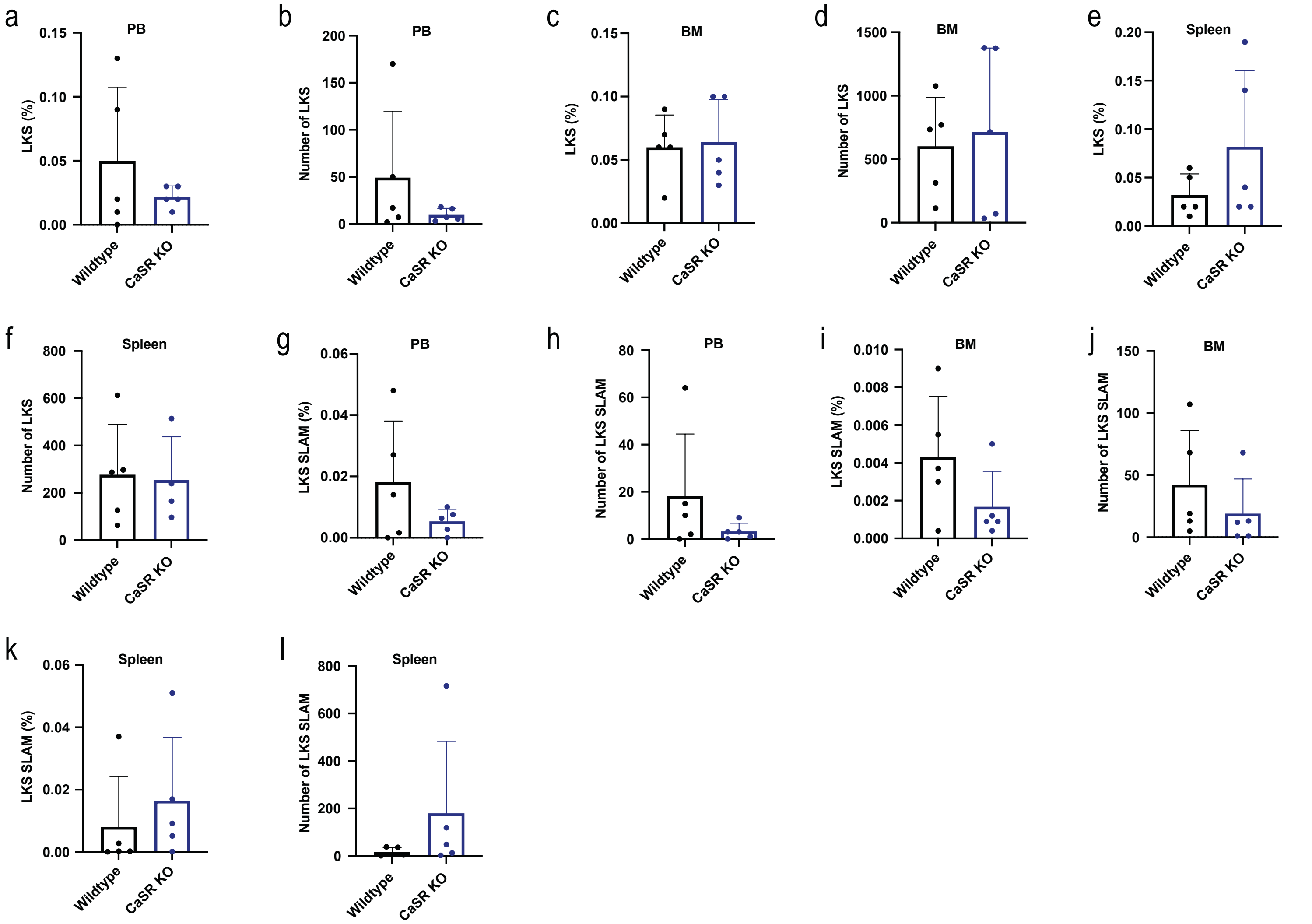

Supplementary Figure 7. a-f) Percentage of single cells and absolute number of Lin^{-} c-Kit^{+} Sca-1^{+} (LKS) cells in peripheral blood (PB) (15 μl) (**a-b**), bone marrow (BM) (one femur) (**c-d**) and spleen (**e-f**) of wildtype (Mx1-Cre$^{-/-}$ CaSR$^{flox/flox}$) (black) or CaSR KO (Mx1-Cre$^{+/-}$ CaSR$^{flox/-flox}$) (blue) mice 10 days after after poly I:C treatment (n=5, two-tailed *t*-test). **g-l**) Percentage of single cells and absolute number of LKS CD48^{-} CD150^{+} (LKS SLAM) cells in peripheral blood (15 μl) (**g-h**), bone marrow (one femur) (**i-j**) and spleen (**k-l**) of wildtype (black) or CaSR KO (blue) mice 10 days after poly I:C treatment (n=5, two-tailed *t*-test).

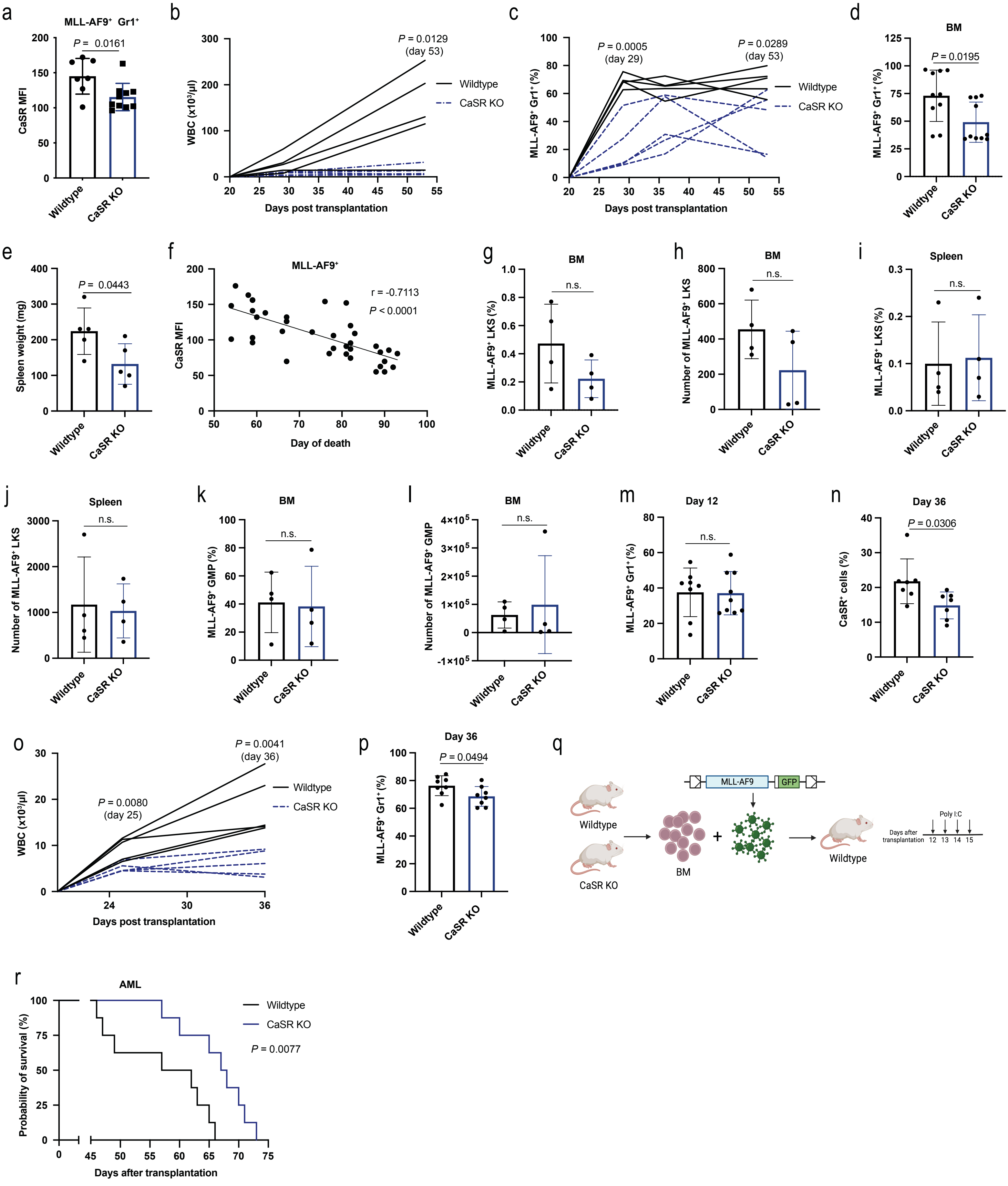

Supplementary Figure 8. a) Mean fluorescence intensity (MFI) of CaSR on MLL-AF9⁺ Gr1⁺ leukocytes in peripheral blood of wildtype (Mx1-Cre$^{-/-}$ CaSR$^{flox/flox}$) mice transplanted with MLL-AF9⁺ wildtype (Mx1-Cre$^{-/-}$ CaSR$^{flox/flox}$) (black) or CaSR KO (Mx1-Cre$^{+/-}$ CaSR$^{flox/flox}$) (blue) leukaemia-initiating cells (LIC) in the AML model (n=9-10, two-tailed *t*-test) 53 days after transplantation. **b-c**) White blood cell counts (WBC) (**b**) and percentage of MLL-AF9⁺ (GFP⁺) Gr1⁺ cells (**c**) in peripheral blood (PB) of recipients of wildtype (black) or CaSR KO (blue) BM cells (n=4, two-tailed *t*-test), transduced with MLL-AF9-expressing retrovirus in the AML model, overtime. Each line

represents an individual mouse. **d**) Percentage of MLL-AF9$^+$ (GFP$^+$) Gr1$^+$ wildtype (black) or CaSR KO (blue) cells of single cells in bone marrow of mice transplanted with MLL-AF9$^+$ (GFP$^+$) wildtype or CaSR KO BM LIC 40 days after transplantation (n=10, two-tailed *t*-test). **e**) Spleen weight of moribund mice which had been transplanted with wildtype (black) or CaSR KO (blue) MLL-AF9-transduced bone marrow (n=5, two-tailed *t*-test). **f**) Correlation between CaSR expression on MLL-AF9$^+$ (GFP$^+$) PB leukocytes (on days 53/54 after transplantation) and day of death of mice transplanted with wildtype or CaSR KO MLL-AF9-transduced bone marrow (n=21). Pearson's correlation (r) and P-values are indicated next to the graph. **g-j**) Percentage of single cells and absolute number of MLL-AF9$^+$ (GFP$^+$) Lin$^-$ c-Kit$^+$ Sca-1$^+$ (LKS) cells in the bone marrow (BM) (one femur) (**g-h**) or spleen (**i-j**) of wildtype recipients of wildtype (black) or CaSR KO (blue) BM cells, transduced with MLL-AF9-expressing retrovirus in the AML model, 12 days after transplantation (n=4, two-tailed *t*-test). Poly I:C had been administered from days 3 to 6 after transplantation. **k-l**) Percentage of single cells (**k**) and absolute number (**l**) of MLL-AF9$^+$ (GFP$^+$) Lin$^-$ IL7R$^-$ c-Kit$^+$ Sca-1$^-$ CD16/32$^+$ CD34$^+$ granulocyte- monocyte progenitors (GMP) in the bone marrow (one femur) of wildtype mice transplanted with wildtype (black) or CaSR KO (blue) BM, transduced with MLL-AF9-expressing retrovirus (n=4, two-tailed *t*-test). **m**) Percentage of MLL-AF9$^+$ (GFP$^+$) Gr1$^+$ wildtype (black) or CaSR KO (blue) cells of single cells in peripheral blood of mice transplanted with MLL-AF9$^+$ (GFP$^+$) wildtype or CaSR KO BM LIC 12 days after transplantation, prior to CaSR depletion by poly I:C treatment (n=8, two-tailed *t*-test). **n**) Percentage of CaSR$^+$ cells of single cells in the peripheral blood of mice transplanted with MLL-AF9$^+$ (GFP$^+$) wildtype (black) or CaSR KO (blue) LIC, determined on day 36 after transplantation. CaSR gene depletion was performed on day 12 after transplantation (n=8, two-tailed *t*-test). **o-p**) White blood cell counts (WBC) (n=4, two-tailed *t*-test) (**o**) and percentage of MLL-AF9$^+$ (GFP$^+$) Gr1$^+$ cells of single cells (n=8, two-tailed *t*-test) (**p**) in peripheral blood of mice transplanted with wildtype (black) or CaSR KO (blue) MLL AF9$^+$ LIC 36 days post transplantation. CaSR gene depletion was performed on day 12 after transplantation. **q-r**) Transplantation scheme (**q**) and Kaplan-Meier-style survival curve (**r**) of wildtype mice transplanted with wildtype (black) or CaSR KO (blue) BM, transduced with MLL-AF9-expressing retrovirus. CaSR deletion with poly I:C was initiated on day 12 after transplantation (n=8, Log-rank test).

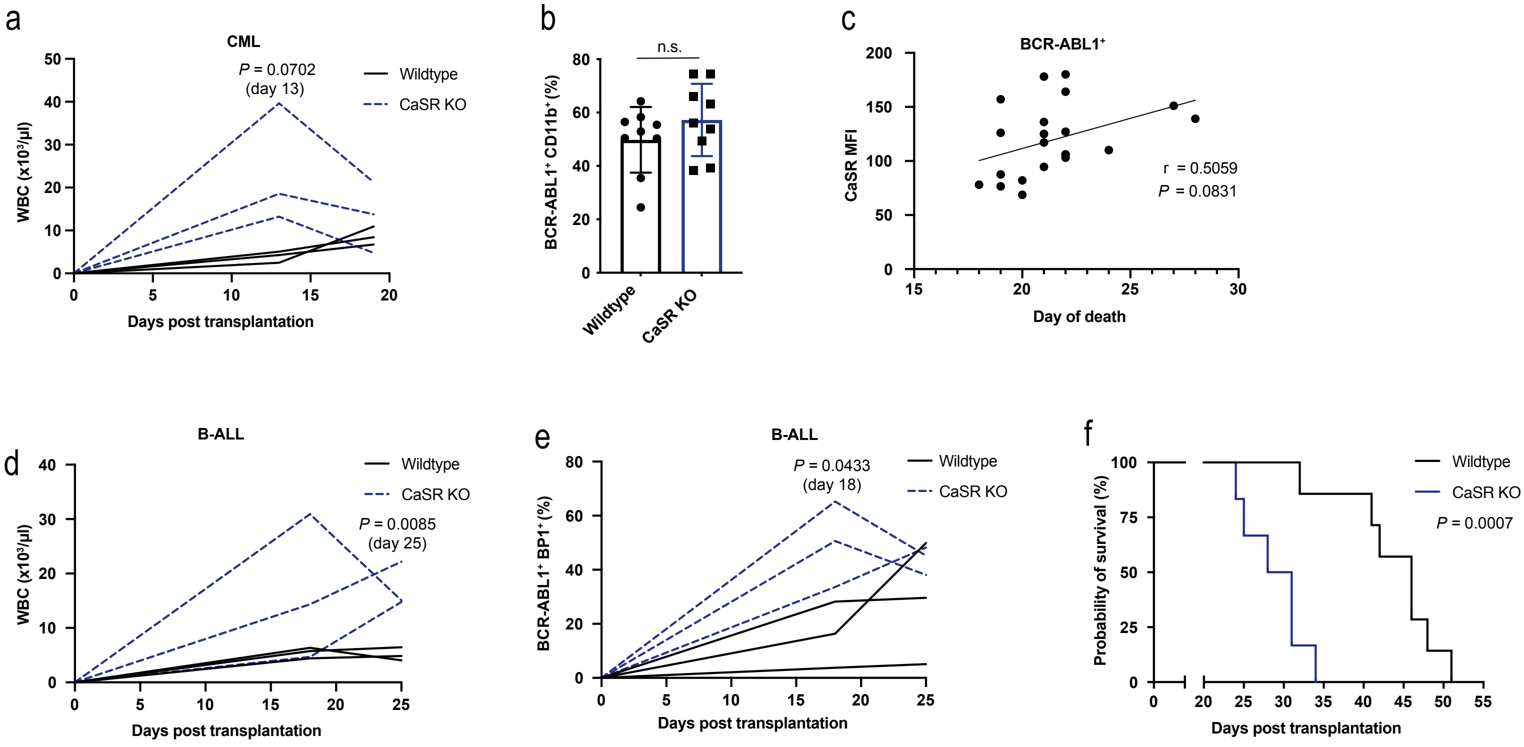

Supplementary Figure 9. a-b) White blood cell count (WBC) (n=3) (**a**) and percentage of BCR-ABL1⁺ (GFP⁺) CD11b⁺ cells of single cells (**b**) in peripheral blood of wildtype (Mx1-Cre$^{-/-}$ CaSR$^{flox/flox}$) mice, transplanted with wildtype (black) or CaSR KO (Mx1-Cre$^{+/-}$ CaSR$^{flox/flox}$) (blue) BM cells, transduced with BCR-ABL1-expressing retrovirus in the CML model 13 days after transplantation (n=9, two-tailed *t*-test). **c**) Correlation between CaSR expression on BCR-ABL1⁺ (GFP⁺) PB leukocytes (on day 15 after transplantation) and day of death of mice transplanted with wildtype or CaSR KO BCR-ABL1-transduced bone marrow in the CML model (n=21). Pearson's correlation (r) and P-values are indicated next to the graph. **d-e**) White blood cell count (WBC) (**d**) and percentage of BCR-ABL1⁺ BP1⁺ pre-B cells of single cells (**e**) in peripheral blood of wildtype recipients of wildtype (black) or CaSR KO (blue) BM, transduced with BCR-ABL1-expressing retrovirus in the B-ALL model (n=3, *t*-test). **f**) Kaplan-Meier-style survival curve of recipient mice transplanted with wildtype (black) or CaSR KO (blue) BM, transduced with BCR-ABL1-expressing retrovirus in the B-ALL model (n=6-7, Log-rank test). All recipient mice were injected with 10 mg/kg poly I:C on days 3-6 after transplantation.

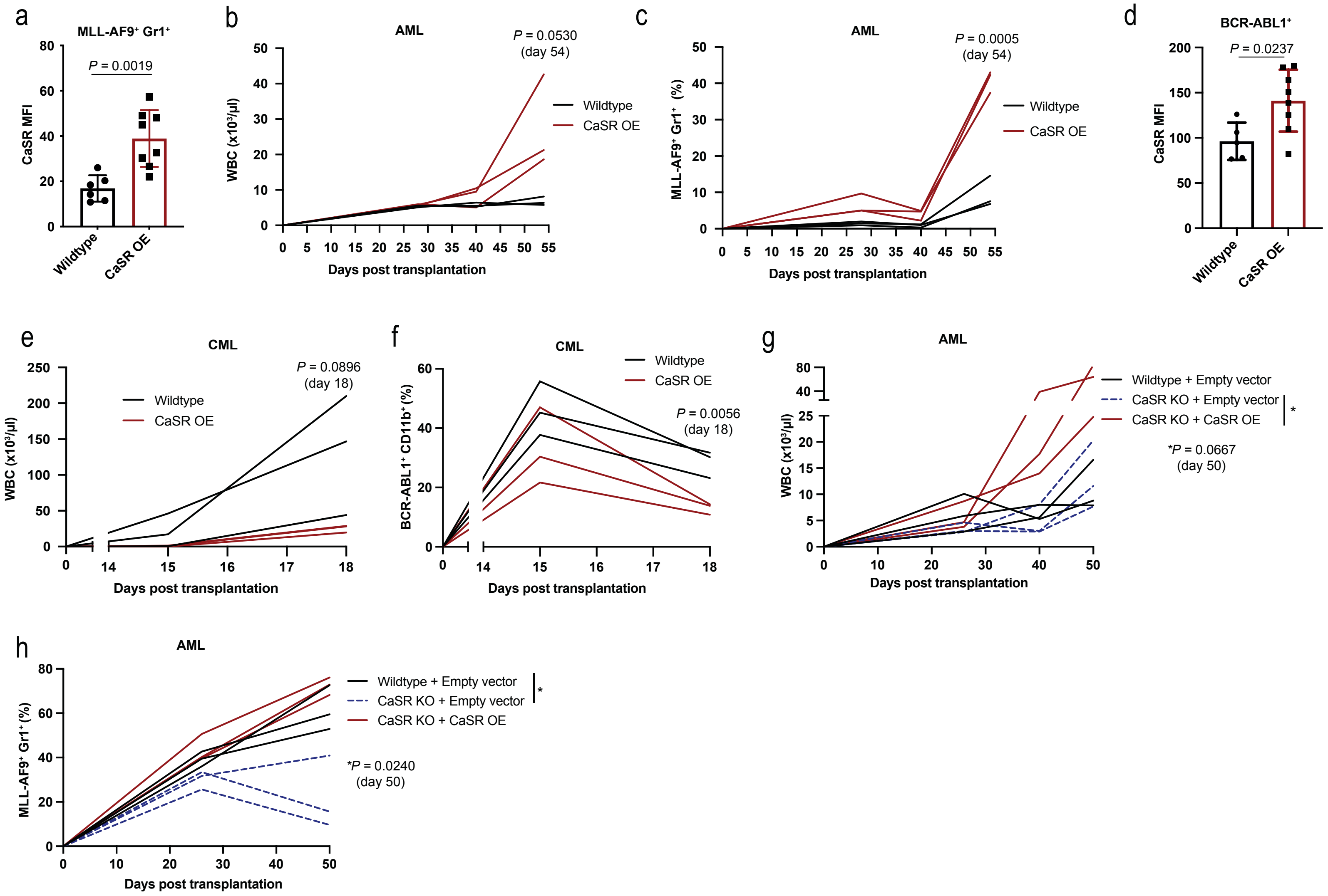

Supplementary Figure 10. a) Mean fluorescence intensity (MFI) of CaSR on MLL-AF9$^+$ Gr1$^+$ leukocytes in peripheral blood of BALB/c wildtype mice transplanted with MLL-AF9$^+$ leukaemia-initiating cells (LIC), co-transduced with empty vector- (black) or CaSR-overexpressing retrovirus (red) in the AML model (n=9-10, two-tailed *t*-test) 54 days post transplantation. **b-c**) White blood cell count (WBC) (**b**) and percentage of MLL-AF9$^+$ (RFP$^+$) Gr1$^+$ cells of single cells (**c**) in peripheral blood of wildtype BALB/c recipients of wildtype (black) or CaSR OE (red) BALB/c BM cells, transduced with MLL-AF9-expressing retrovirus (n=9-10, two-tailed *t*-test). **d**) Mean fluorescence intensity (MFI) of CaSR on BCR-ABL1$^+$ (RFP$^+$) leukocytes in peripheral blood of wildtype BALB/c mice transplanted with BCR-ABL1$^+$ BALB/c LIC, cotransduced with empty vector- (black) or CaSR-overexpressing retrovirus (red) in the CML model 15 days after transplantation (n=6-8, two-tailed *t*-test). **e-f**) White blood cell count (WBC) (**e**) and percentage of BCR-ABL1$^+$ CD11b$^+$ cells of single cells (**f**) in peripheral blood of BALB/c recipients of wildtype (black) or CaSR OE (red) BALB/c BM cells, transduced with BCR-ABL1-expressing retrovirus in the CML model (n=6-8, two-tailed *t*-test). **g-h**) White blood cell count (WBC) (**g**) and percentage of MLL-AF9$^+$ (RFP$^+$) Gr1$^+$ cells of single cells (**h**) in peripheral blood of wildtype (Mx1-Cre$^{-/-}$ CaSR$^{flox/flox}$) recipients of wildtype (black), CaSR KO (Mx1-Cre$^{+/-}$ CaSR$^{flox/flox}$) (blue) or CaSR KO cells, transduced with CaSR OE-expressing retrovirus (red) cotransduced with MLL-AF9-expressing retrovirus (n=8, one-way ANOVA, Tukey test). The wildtype BM (black) and the CaSR KO BM (blue) was cotransduced with empty vector-expressing retrovirus.

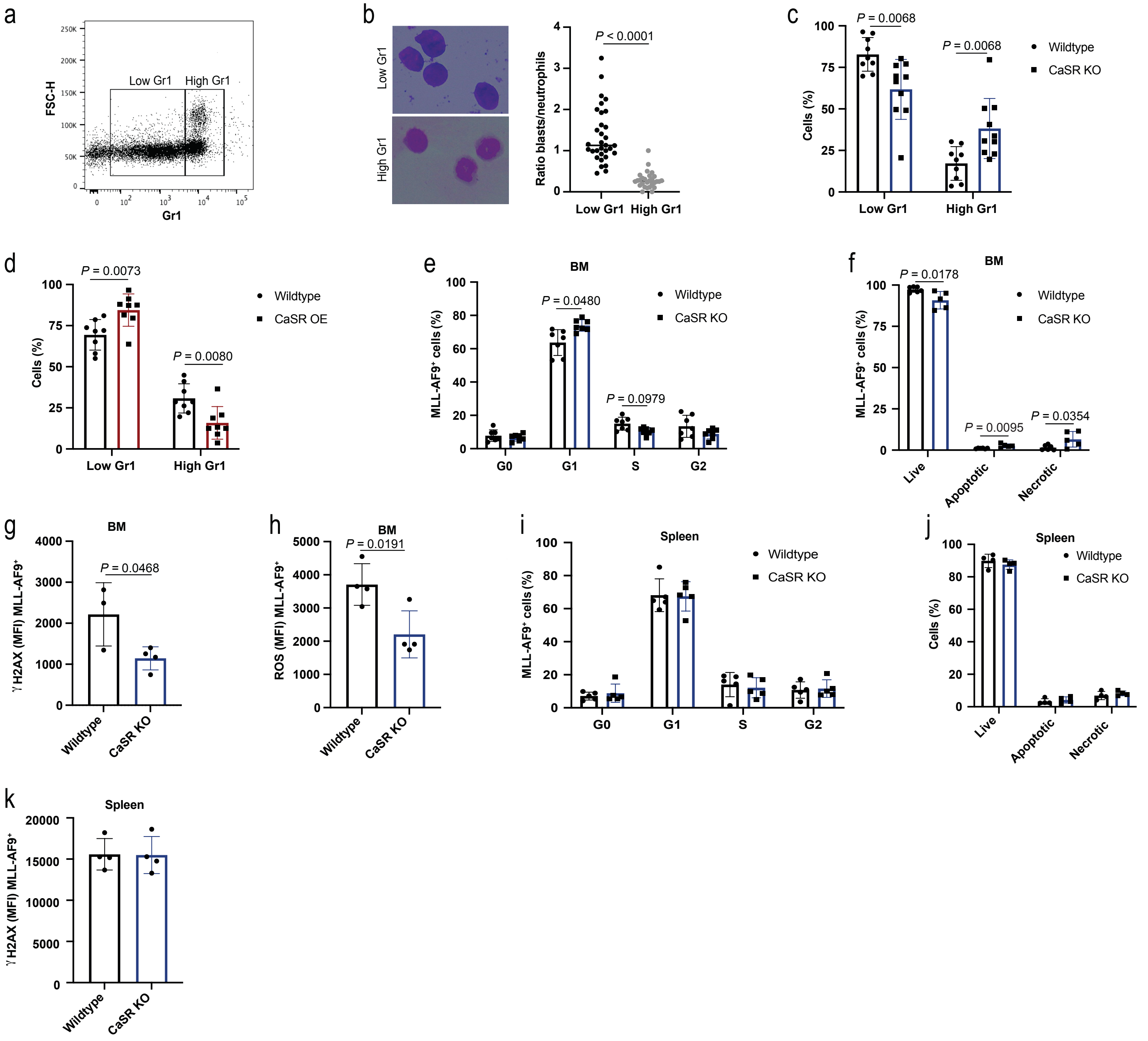

Supplementary Figure 11. a) Representative FACS plot of the percentage of low versus high Gr1 surface expression on MLL-AF9$^+$ (GFP$^+$) cells in the peripheral blood (PB) of mice. **b**) Representative Giemsa staining (left) and quantification (right) of the ratio of blasts/neutrophils in sorted MLL-AF9$^+$ (GFP$^+$) leukocytes with low or high surface expression of Gr1. The MLL-AF9$^+$ (GFP$^+$) leukocytes were isolated from the BM of wildtype (Mx1-Cre$^{-/-}$ CaSR$^{flox/flox}$) recipients of wildtype BM transduced with MLL-AF9-expressing retrovirus in the AML model on day 40 after transplantation, based on high or low expression of Gr1 (n=28-32, two-tailed Mann-Whitney test). **c**) Percentage of MLL-AF9$^+$ cells of single cells with low or high surface expression of Gr1 in the PB of wildtype mice which had been transplanted with wildtype (black) or CaSR KO (Mx1-Cre$^{+/-}$ CaSR$^{flox/flox}$) (blue) BM, transduced with MLL-AF9-expressing retrovirus on day 36 after transplantation (n=9-10, two-way ANOVA, Sidak´s test). **d**) Percentage of MLL-AF9$^+$ cells of single cells with low or high surface expression of Gr1 in the PB of WT BALB/c mice which had been transplanted with wildtype BALB/c MLL-AF9$^+$ bone marrow, co-transduced with empty vector- (black) or CaSR overexpressing (OE)- (red) retrovirus on day 40 after transplantation (n=8, two-way ANOVA, Sidak´s test). **e**) Cell cycle status of MLL-AF9$^+$ (GFP$^+$) cells of single cells in the BM of wildtype recipients of wildtype (black) or CaSR KO (blue) MLL-AF9$^+$ leukaemia-initiating cells (LIC) using Ki67 staining on day 40 after transplantation (n=7, two-way ANOVA, Sidak´s test). **f**) Percentage of MLL-AF9$^+$ (GFP$^+$) live, apoptotic or necrotic MLL-AF9$^+$ (GFP$^+$) cells of single cells in the bone marrow of mice transplanted with wildtype (black) or CaSR KO (blue) MLL-AF9$^+$ LIC, detected by annexin V and DAPI staining, on day 40 after transplantation (n=5-6, two-way ANOVA, Sidak´s test). **g**) Median fluorescence intensity (MFI) of γH2AX in MLL-AF9$^+$ cells of single cells in the BM of wildtype recipients of wildtype (black) or CaSR KO (blue) MLL-AF9$^+$ LIC, 40 days after transplantation (n=3-4, two-tailed *t*-test). **h**) Mean fluorescence intensity (MFI) of reactive oxygen species (ROS) in MLL-AF9$^+$ cells of single cells in the BM of mice transplanted with MLL-AF9$^+$ wildtype (black) or CaSR KO (blue) BM on day 40 after transplantation (n=5,two-tailed *t*-test). **i**) Cell cycle status of MLL-AF9$^+$ (GFP$^+$) cells of single cells in the spleen of wildtype recipients of wildtype (black) or CaSR KO (blue) MLL-AF9$^+$ LIC using Ki67 and DAPI staining on day 40 after transplantation (n=7, two-way ANOVA, Sidak´s test). **j**) Percentage of MLL-AF9$^+$ (GFP$^+$) live, apoptotic or necrotic cells of single cells in the spleen of mice transplanted with wildtype (black) or CaSR KO (blue) MLL-AF9$^+$ LIC, detected by annexin V and DAPI staining, on day 40 after transplantation (n=4, two-way ANOVA, Sidak´s test). **k**) Median fluorescence intensity (MFI) of γH2AX in MLL-AF9$^+$ cells of single cells in the spleen of wildtype recipients of wildtype (black) or CaSR KO (blue) MLL-AF9$^+$ LIC, 40 days after transplantation (n=3-4, two-tailed *t*-test).

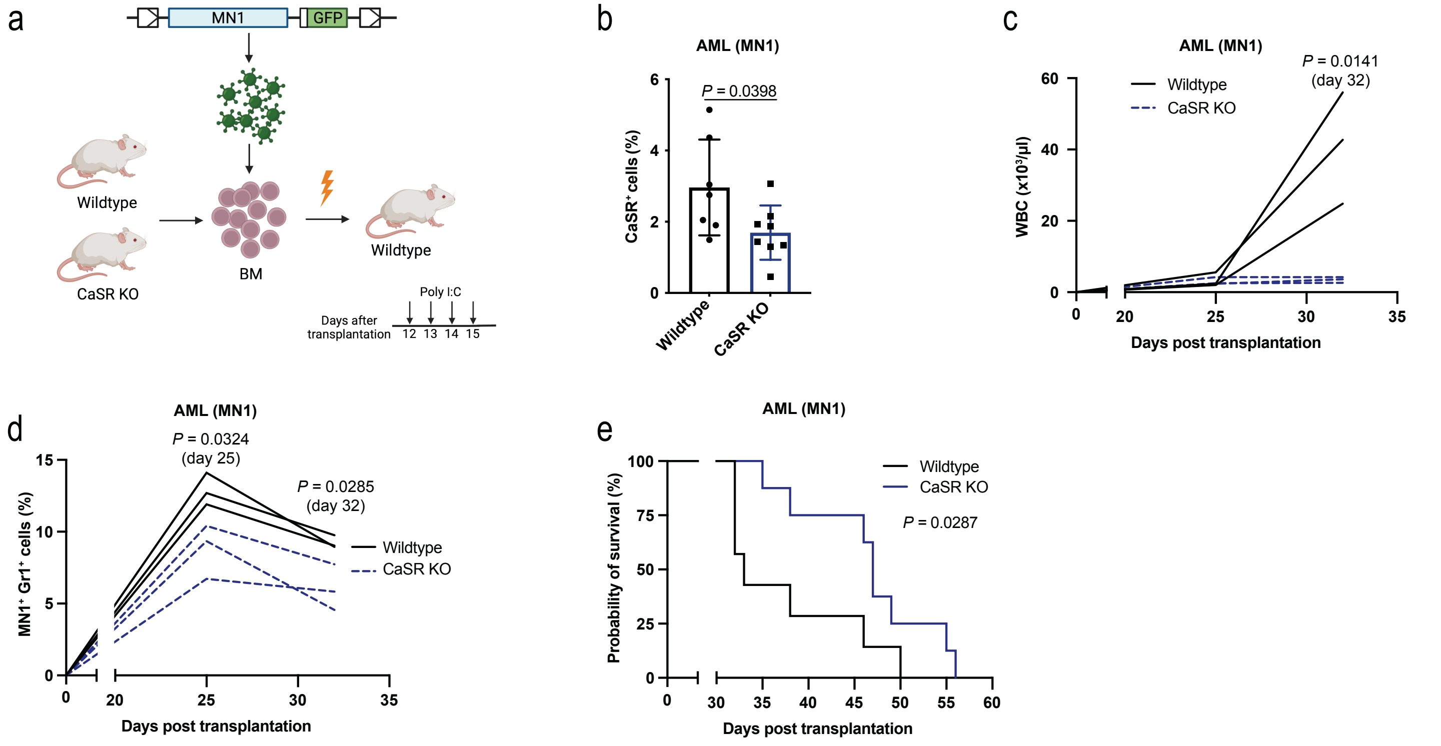

Supplementary Figure 12. a) Schematic representation of the transplantation of mice with wildtype or CaSR KO BM cells, transduced with MN1-expressing retrovirus. **b**) Percentage of $CaSR^+$ cells of single cells in peripheral blood of wildtype (Mx1-$Cre^{-/-}$ $CaSR^{flox/flox}$) mice transplanted with wildtype (black) or CaSR KO (Mx1-$Cre^{+/-}$ $CaSR^{flox/flox}$) (blue) $MN1^+$ LIC, measured on day 25 after transplantation (n=7-8, two-tailed *t*-test). **c-d**) White blood cell counts (WBC) (**c**) and percentage of $MN1^+$ (GFP^+) $Gr1^+$ leukocytes of single cells (**d**) in peripheral blood of wildtype mice transplanted with $MN1^+$ wildtype (black) or CaSR KO (blue) bone marrow cells (n=7-8, two-tailed *t*-test) over time. **e**) Kaplan-Meier-style survival curve of wildtype mice transplanted with $MN1^+$ wildtype (black) or CaSR KO (blue) BM cells (n=7-8, Log-rank test).

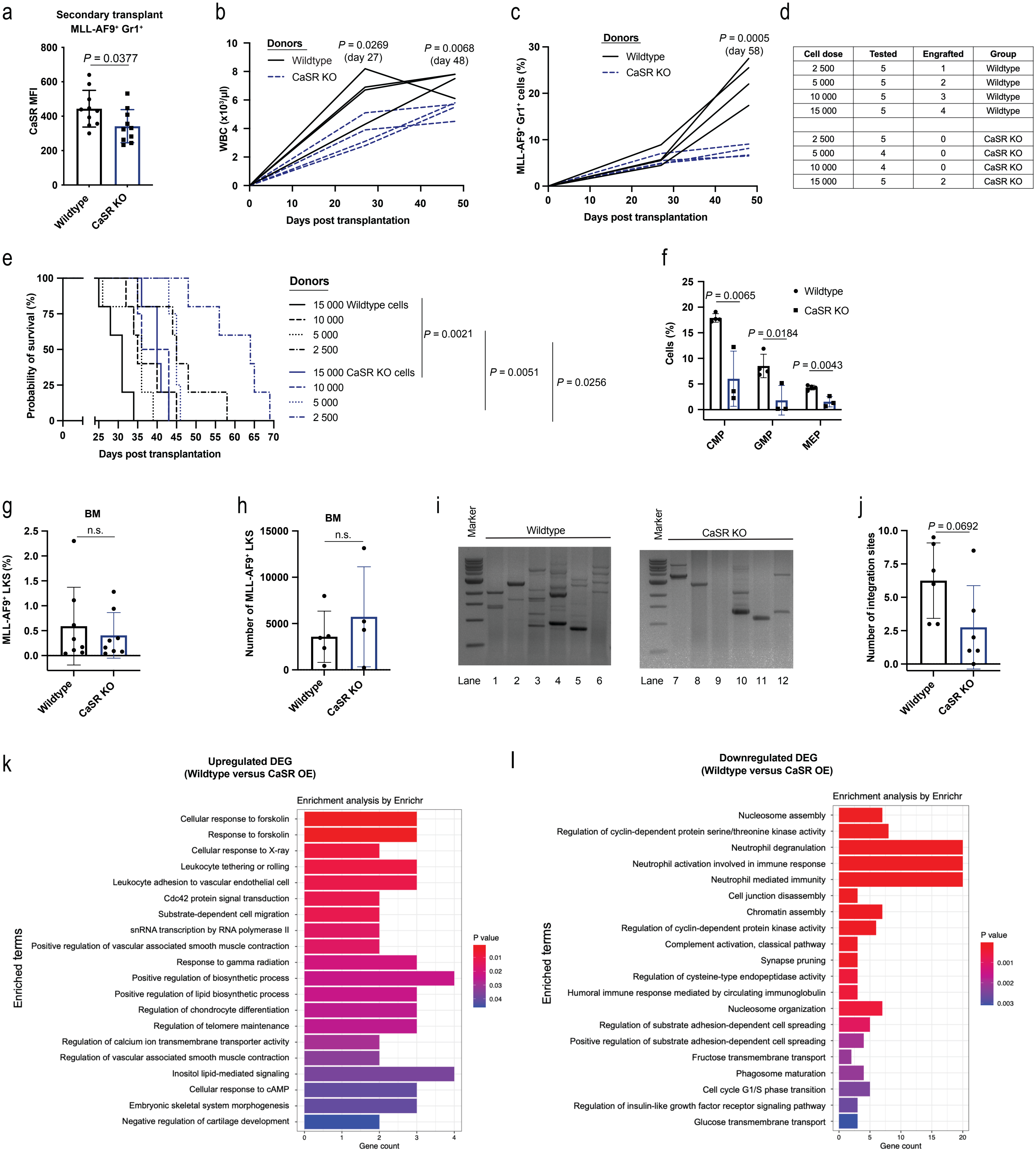

Supplementary Figure 13. a) Mean fluorescence intensity (MFI) of CaSR on MLL-AF9⁺ Gr1⁺ peripheral blood leukocytes of secondary recipient mice, transplanted with sorted wildtype or CaSR KO MLL-AF9⁺ (GFP⁺) Lin⁻ BM cells from primary mice with established MLL-AF9-induced AML on day 27 after secondary transplantation (n=10, two-tailed *t*-test). **b-c**) White blood cell count (WBC) (**b**) and percentage of MLL-AF9⁺ (GFP⁺) Gr1⁺ cells (**c**) in the peripheral blood of wildtype secondary recipient mice, transplanted with sorted wildtype or CaSR KO MLL-AF9⁺ (GFP⁺) Lin⁻ BM cells from mice with established MLL-AF9-induced AML (n=4, two-tailed *t*-test). **d**) Table summarizing the transplanted cell dose, the number of mice per group (tested), the number of mice engrafting, and the type of donor bone marrow (group) in a limiting dilution transplantation assay. Sorted MLL-AF9⁺ (GFP⁺) Lin⁻ BM cells were transplanted into recipient animals

at varying doses (2,500-15,000 cells per mouse) in addition to 2 x 10^6 supporting BM cells. Leukaemia stem cell (LSC) frequency was determined by Extreme Limiting Dilution Analysis (ELDA). Donor cell non-engraftment was defined as the recipient mice having less than 8,000 leukocytes/µl and less than 20 % MLL-AF9$^+$ (GFP$^+$) Gr1$^+$ cells of total leukocytes in PB on day 24 after secondary transplantation. **e**) Kaplan-Meier-style survival curves of wildtype (Mx1-Cre$^{-/-}$ CaSR$^{flox/flox}$) secondary recipient mice transplanted with different doses of wildtype or CaSR KO (Mx1-Cre$^{+/-}$ CaSR$^{flox/flox}$) sorted MLL-AF9$^+$ (GFP$^+$) Lin$^-$ BM cells (2,500-15,000 cells per mouse) plus supporting BM as in (**d**) in a limiting-dilution transplantation assay (n=4-5, Log-rank test). **f**) Percentage of MLL-AF9$^+$ (GFP$^+$) common myeloid progenitors (CMP: Lin$^-$ IL7R$^-$ c Kit$^+$ Sca-1$^-$ CD16/32$^-$ CD34$^-$), granulocyte monocyte progenitors (GMP: Lin$^-$ IL7R$^-$ c-Kit$^+$ Sca-1$^-$ CD16/32$^+$ CD34$^+$) and megakaryocyte erythroid progenitors (MEP: Lin$^-$ IL7R$^-$ c-Kit$^+$ Sca-1$^-$ CD16/32$^-$ CD34$^+$) cells in the BM of wildtype mice transplanted with MLL-AF9$^+$ wildtype or CaSR KO BM cells in the AML model on day 40 after transplantation (n=3-4, two-way ANOVA Sidak´s test). 10 mg/kg of poly I:C was administered on days 3 to 6 after transplantation. **g-h**) Percentage of single cells (**g**) and absolute number (**h**) of Lin$^-$ c-Kit$^+$ Sca-1$^+$ (LKS) cells in the BM (one femur) of wildtype mice transplanted with wildtype (black) or CaSR KO (blue) MLL-AF9$^+$ leukaemia-initiating cells (LIC), determined on day 40 after transplantation (n=4-5, two-tailed *t*-test).10 mg/kg of poly I:C was administered on days 3 to 6 after transplantation. **i-j**) Representative images of agarose gel electrophoresis (**I**) and its quantification (**J**) of the DNA products amplified by long-distance inverse (LDI) PCR, derived from splenic tissue of moribund wildtype mice which had been transplanted with wildtype (lanes 1-6; black) or CaSR KO (lanes 7-12; blue) MLL-AF9-transduced BM in the AML model (n=6, two-tailed *t*-test). **k-l**) Gene ontology (GO) analysis of significantly upregulated (**k**) or downregulated (**l**) genes in sorted MLL-AF9$^+$ (RFP$^+$) Lin$^-$ cells from the BM of wildtype mice transplanted with wildtype MLL-AF9$^+$ LIC, co-transduced with CaSR-overexpressing or empty vector-expressing retrovirus in the AML model. The bar graph summarizes non-redundant terms with P-values provided by the colour coding.

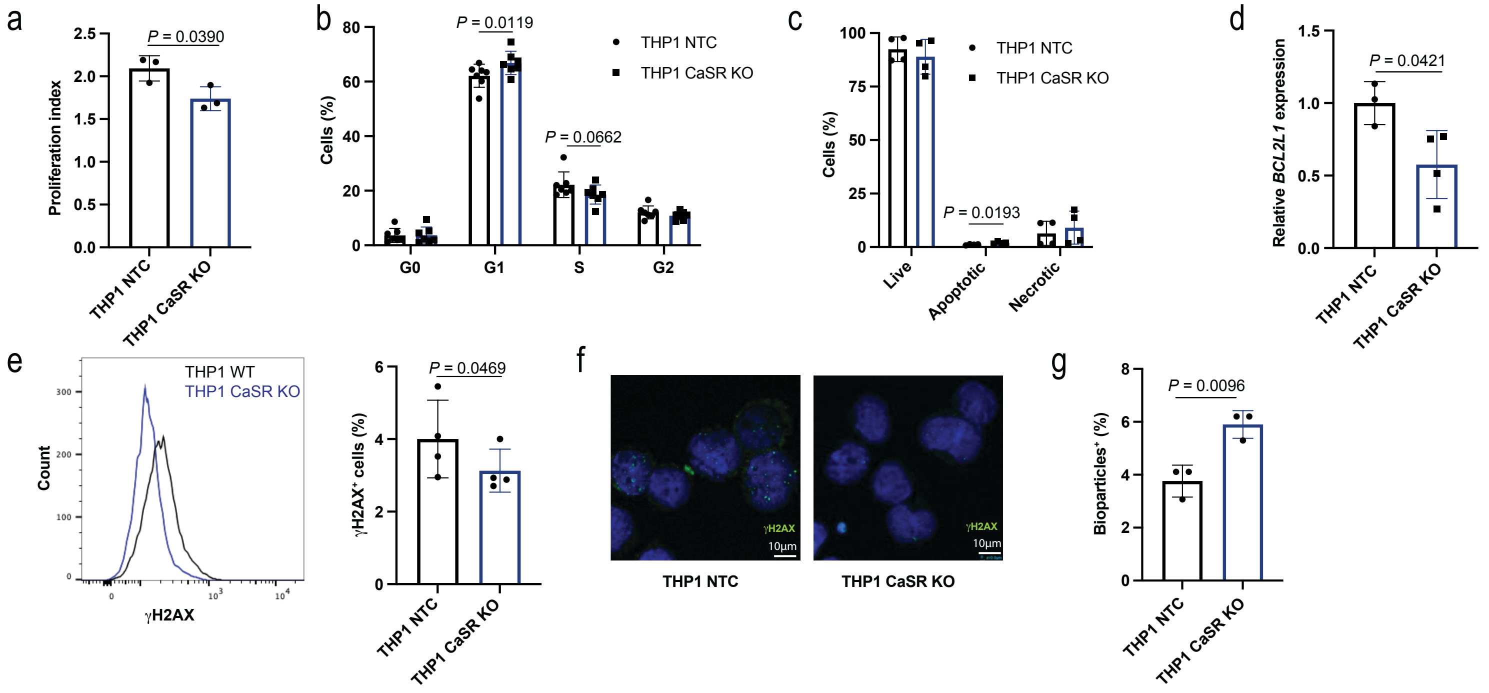

Supplementary Figure 14. a) Proliferation of non-target control (NTC) (black) or CaSR KO (blue) THP1 cells after 72 h of culture, assessed by carboxyfluorescein succinimidyl ester (CFSE) staining (n=3,two-tailed *t*-test). **b**) Cell cycle status of NTC (black) or CaSR KO (blue) THP1 cells by Ki67 and 4′,6-diamidino--2-phenylindole (DAPI) staining (n=7, two-way ANOVA, Sidak´s test). **c**) Percentage of live, apoptotic or necrotic NTC (black) or CaSR KO (blue) THP1 cells of single cells, detected by annexin V and DAPI staining (n=4, two-way ANOVA, Sidak´s test). **d**) Relative expression of *BCL2L1* (BCL-XL) in NTC (black) or CaSR KO (blue) THP1 cells by RT-qPCR analysis (n=4, two-tailed *t*-test). Glyceraldehyde 3-phosphate dehydrogenase (*GAPDH*) was used as a housekeeping control. **e**) Representative histogram and percentage of γH2AX+ NTC (black) or CaSR KO (blue) THP1 cells of single cells, as tested by flow cytometry (n=4, two-tailed *t*-test). **f**) Immunofluorescence (IF) staining for γH2AX (green) in NTC (left) or CaSR KO (right) THP1 cells. DNA double strand breaks (DSBs) were detected by staining with γH2AX and AF-647 (foci shown in green). Nuclear staining by DAPI is shown in blue. The scale bar represents 10 μm. **g**) Percentage of NTC (black) or CaSR KO (blue) THP1 cells of single cells, which have phagocytosed phycoerythrin (PE)-conjugated pHrodo E. coli bioparticles after an exposure of 90 minutes (n=3, two-tailed *t*-test).

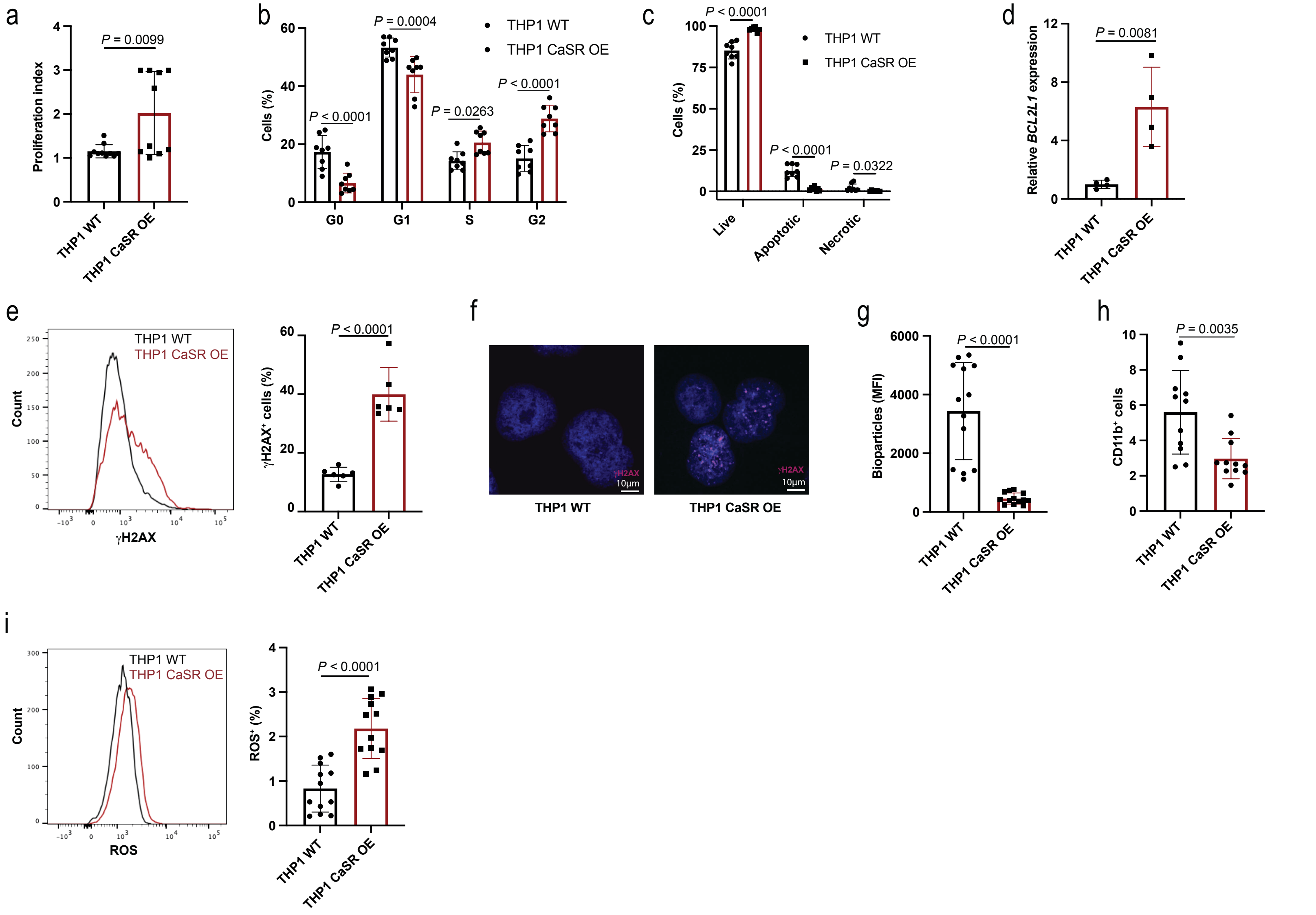

Supplementary Figure 15. **a**) Proliferation of WT (black) or CaSR overexpressing (OE) (red) THP1 cells after 72 h of culture, assessed by carboxyfluorescein succinimidyl ester (CFSE) staining (n=10, two-tailed *t*-test). **b**) Cell cycle status of wildtype (WT) (black) or CaSR OE (red) THP1 cells by Ki67 and 4′,6-diamidino-2-phenylindole (DAPI) staining (n=8, two-way ANOVA, Sidak´s test). **c**) Percentage of live, apoptotic or necrotic WT (black) or CaSR OE (red) THP1 cells detected by annexin V and DAPI staining (n=8, two-way ANOVA, Sidak´s test). **d**) Relative expression of *BCL2L1* (BCL-XL) in WT (black) or CaSR OE (red) THP1 cells by RT-qPCR analysis (n=4, two-tailed *t*-test). Glyceraldehyde 3-phosphate dehydrogenase (*GAPDH*) was used as a housekeeping control. **e**) Representative histogram and percentage of γH2AX$^+$ WT (black) or CaSR OE (red) THP1 cells of single cells, as tested by flow cytometry (n=6, two-tailed *t*-test). **f**) Immunofluorescence (IF) staining for γH2AX (magenta) in WT (left) or CaSR OE (right) THP1 cells. DNA double strand breaks (DSBs) were detected by staining with γH2AX and AF-647 (foci shown in magenta). Nuclear staining by DAPI is shown in blue. The scale bar represents 10 μm. **g**) Percentage of WT (black) or CaSR OE (red) THP1 cells of single cells, which have phagocytosed phycoerythrin (PE)-conjugated pHrodo E. coli bioparticles after an exposure of 90 minutes (n=13, two-tailed *t*-test). **h**) Percentage of CD11b$^+$ cells of single cells on WT (black) or CaSR OE (red) THP1 cells (n=11, two-tailed *t*-test). **i**) Representative histogram and percentage of reactive oxygen species (ROS)$^+$ cells of all WT (black) versus CaSR OE (red) THP1 cells, as determined by flow cytometry (n=12, two-tailed *t*-test).

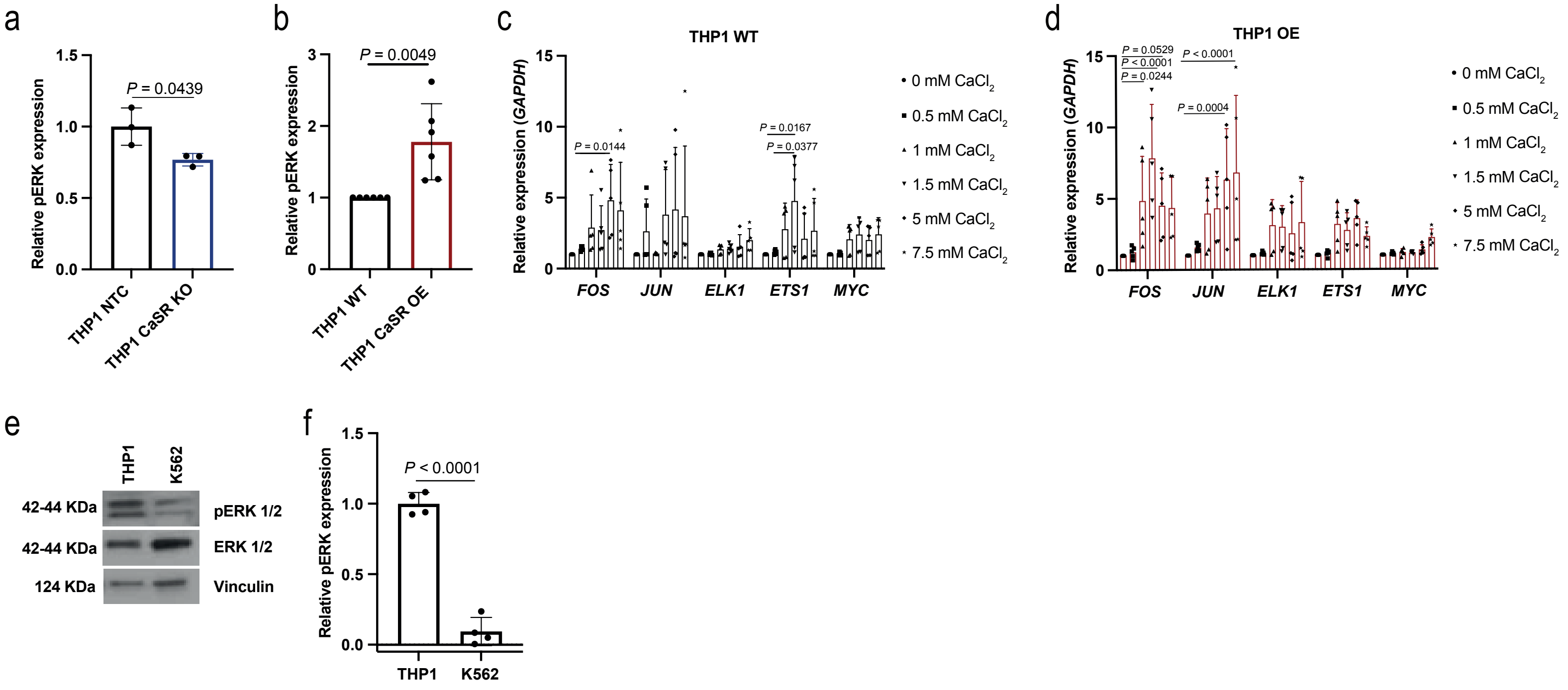

Supplementary Figure 16. a) Quantification of the band intensity of pERK1/2 in lysates of non-target control (NTC) versus CaSR KO THP1 cells, normalized by ERK1/2, in the immunoblot shown in Figure 4c (n=3, two-tailed *t*-test). **b**) Quantification of the band intensity of pERK1/2 in lysates of WT versus CaSR OE THP1 cells, normalized by ERK1/2, in the immunoblot shown in Figure 4D (n=6, two-tailed *t*-test). **c-d** Relative expression of pERK 1/2 target genes, determined by RT-qPCR in THP1 WT (**c**) versus THP1 CaSR OE cells (**d**) after treatment with 0-7.5 mM of CaCl$_2$ for 4 h (n=5, ANOVA, Tukey test). Glyceraldehyde 3-phosphate dehydrogenase (*GAPDH*) was used as housekeeping gene. Fos proto-oncogene=*FOS*, Jun proto-oncogene=*JUN*, ETS transcription factor ELK1=*ELK1*, ETS proto-oncogene 1=*ETS1*, MYC proto-oncogene=*MYC*. **e-f**) Representative immunoblot (**e**) and its quantification (**f**) of pERK1/2 (42, 44 kDa), ERK1/2 (42, 44 kDa) and vinculin (124 kDa) expression in lysates of THP1 or K562 cells (n=4, two-tailed *t*-test). The blot is representative of four independent experiments.

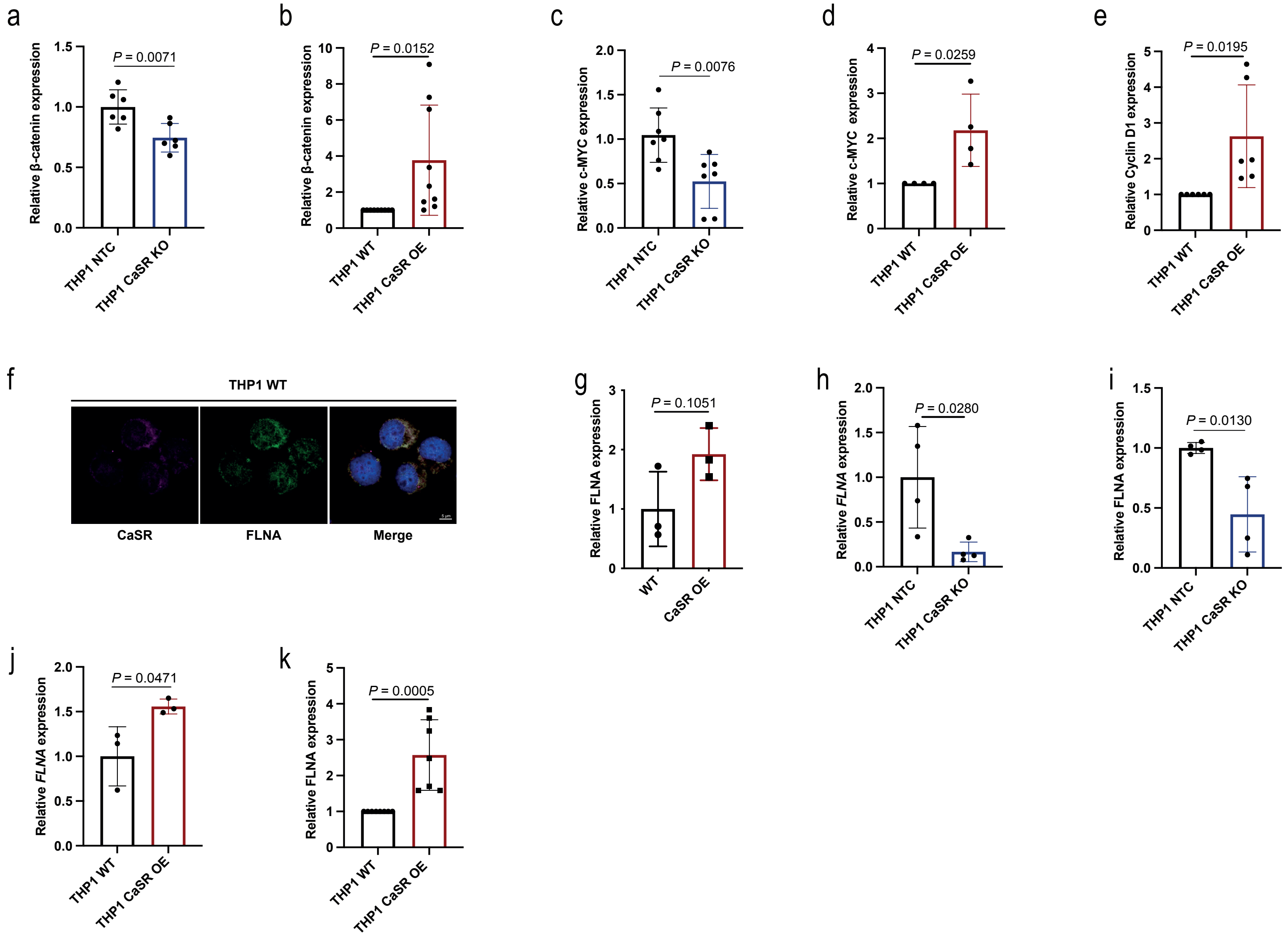

Supplementary Figure 17. a) Quantification of the band intensity of β-catenin in lysates of non-target control (NTC) versus CaSR KO THP1 cells, normalised by vinculin, in the immunoblot shown in Figure 4g (n=6, two-tailed *t*-test). **b**) Quantification of the band intensity of β-catenin in lysates of WT versus CaSR OE THP1 cells, normalised by GAPDH, in the immunoblot shown in Figure 4h (n=9, two-tailed *t*-test). **c**) Quantification of the band intensity of c-MYC in lysates of NTC versus CaSR KO THP1 cells, normalised by vinculin, in the immunoblot shown in Figure 4g (n=8, two-tailed *t*-test). **d**) Quantification of the band intensity of c-MYC in lysates of WT versus CaSR OE THP1 cells, normalised by GAPDH, in the immunoblot shown in Figure 4h (n=4, two-tailed *t*-test). **e**) Quantification of the band intensity of cyclin D1 in lysates of WT versus CaSR OE THP1 cells, normalised by GAPDH, in the immunoblot shown in Figure 4h (n=6, two-tailed *t*-test). **f**) Immunofluorescence (IF) staining for CaSR (magenta) and FLNA (green) in THP1 WT cells. Nuclear staining by DAPI is shown in blue. The images are representative of three independent experiments. The scale bar represents 5 *μ*m. **g**) Quantification of the band intensity of FLNA in lysates of wildtype versus CaSR OE BM cells, normalised by actin, in the immunoblot shown in Figure 5c (n=3, two-tailed *t*-test). **h**) Relative expression of *FLNA* in NTC versus CaSR KO THP1 cells, determined by RT-qPCR (n=4, two-tailed *t*-test). **i**) Quantification of the band intensity of FLNA in lysates of NTC versus CaSR KO THP1 cells, normalised by GAPDH, in the immunoblot shown in Figure 5d (n=4, two-tailed *t*-test). **j**) Relative expression of *FLNA* in WT versus CaSR OE THP1 cells, determined by RT-qPCR (n=4, two-tailed *t*-test). **k**) Quantification of the band intensity of FLNA in lysates of WT versus CaSR OE THP1 cells, normalised by GAPDH, in the immunoblot shown in Figure 5d (n=7, two-tailed *t*-test).

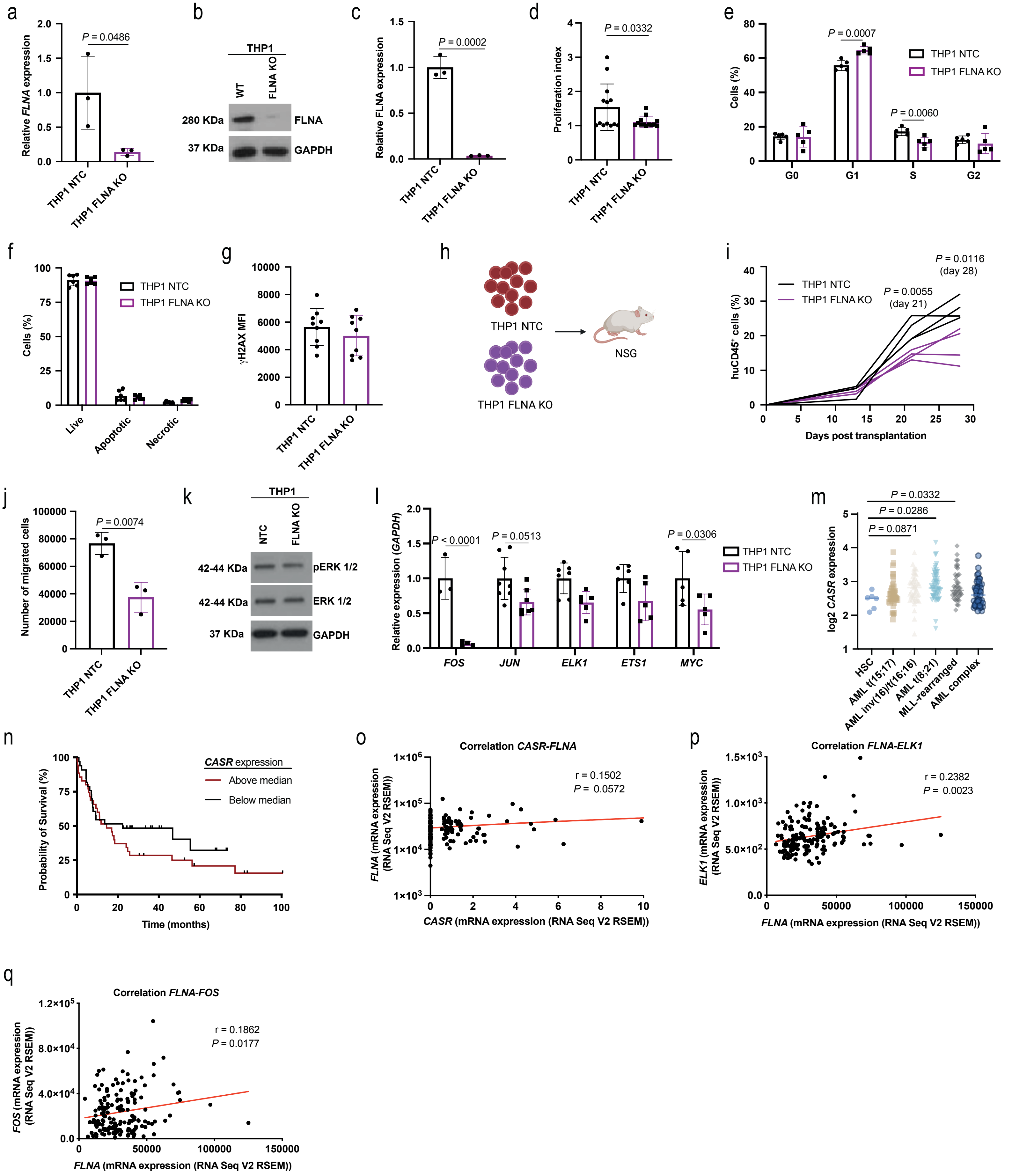

Supplementary Figure 18. a) Relative expression of *FLNA* in NTC (black) versus FLNA KO (purple) THP1 cells, determined by RT-qPCR (n=3, two-tailed *t*-test). **b-c**) Representative immunoblot (**b**) and its quantification (**C**) of FLNA (280 kDa) and GAPDH (37 kDa) expression in lysates of NTC versus FLNA KO THP1 cells (n=3, two-tailed *t*-test). **d**) Proliferation of NTC (black) or FLNA KO (purple) THP1 cells after 72 h of culture, assessed by carboxyfluorescein succinimidyl ester (CFSE) staining (n=13, two-tailed *t*-test). **e**) Cell cycle

status of NTC (black) or FLNA KO (purple) THP1 cells by Ki67 and 4′,6-diamidino- 2-phenylindole (DAPI) staining (n=5, two-way ANOVA, Sidak´s test). **f**) Percentage of live, apoptotic or necrotic NTC (black) or FLNA KO (purple) THP1 cells detected by annexin V and DAPI staining (n=3, two-way ANOVA, Sidak´s test). **g**) Mean fluorescence intensity (MFI) of γH2AX in NTC (black) or FLNA KO (purple) THP1 cells of single cells, as tested by flow cytometry (n=9, two-tailed *t*-test). **h-i**) Transplantation scheme (**H**) and percentage of human $CD45^+$ leukocytes (**I**) in the peripheral blood of NOD SCID interleukin-2 receptor γ knockout (NSG) mice transplanted with $1x10^6$ NTC (black) or FLNA KO (purple) THP1 cells (n=8, two-tailed *t*-test). **j**) Number of NTC versus FLNA KO THP1 cells, which had migrated in a transwell (8 *µ*m pore size) towards C-X-C motif chemokine 12 (CXCL12) within 2 h (n=3, two-tailed *t*-test). **k**) Representative immunoblot of ERK1/2 (42, 44 kDa), phosphoERK1/2 (pERK1/2) and GAPDH expression in lysates of NTC versus FLNA KO THP1 cells. The blot is representative of five independent experiments. **l**) Relative expression of target genes downstream of pERK1/2 in NTC versus FLNA KO THP1 cells, as tested by RT-qPCR (n=3-8, two-way ANOVA, Sidak´s test). Fos proto-oncogene=*FOS*, Jun proto-oncogene=*JUN*, ETS transcription factor ELK1=*ELK1*, ETS proto-oncogene 1=*ETS1*, MYC proto-oncogene=*MYC*. **m**) Log2 expression of *CASR* in HSC from healthy individuals (n= 6) or in unsorted BM cells of patients with AML t(15;17) (n=54), AML inv(16)/t(16;16) (n=47), AML t(8;21) (n=60), MLL-rearranged (AML (t(11q23)/MLL)) (n=43) or AML with complex karyotype (n=46) (one-way ANOVA, Dunnett test), taken from the Bloodspot database. **n**) Kaplan-Meier-style survival of patients with AML with high (n=73) or low (n=100) expression of *CASR*. The curves were generated using the publicly available TCGA dataset (Log-rank test). **o-q**) Results of TCGA data analysis. Correlation of the mRNA levels of *CASR* and *FLNA* (**o**), *FLNA* and *ELK1* (**p**) (n=69) and *FLNA* and *FOS* (**q**) (n=162) in patients with AML using the data from the NEJM 2013 study, accessed via the cBioPortal. Pearson's correlation coefficient (r) and the P-value are shown.

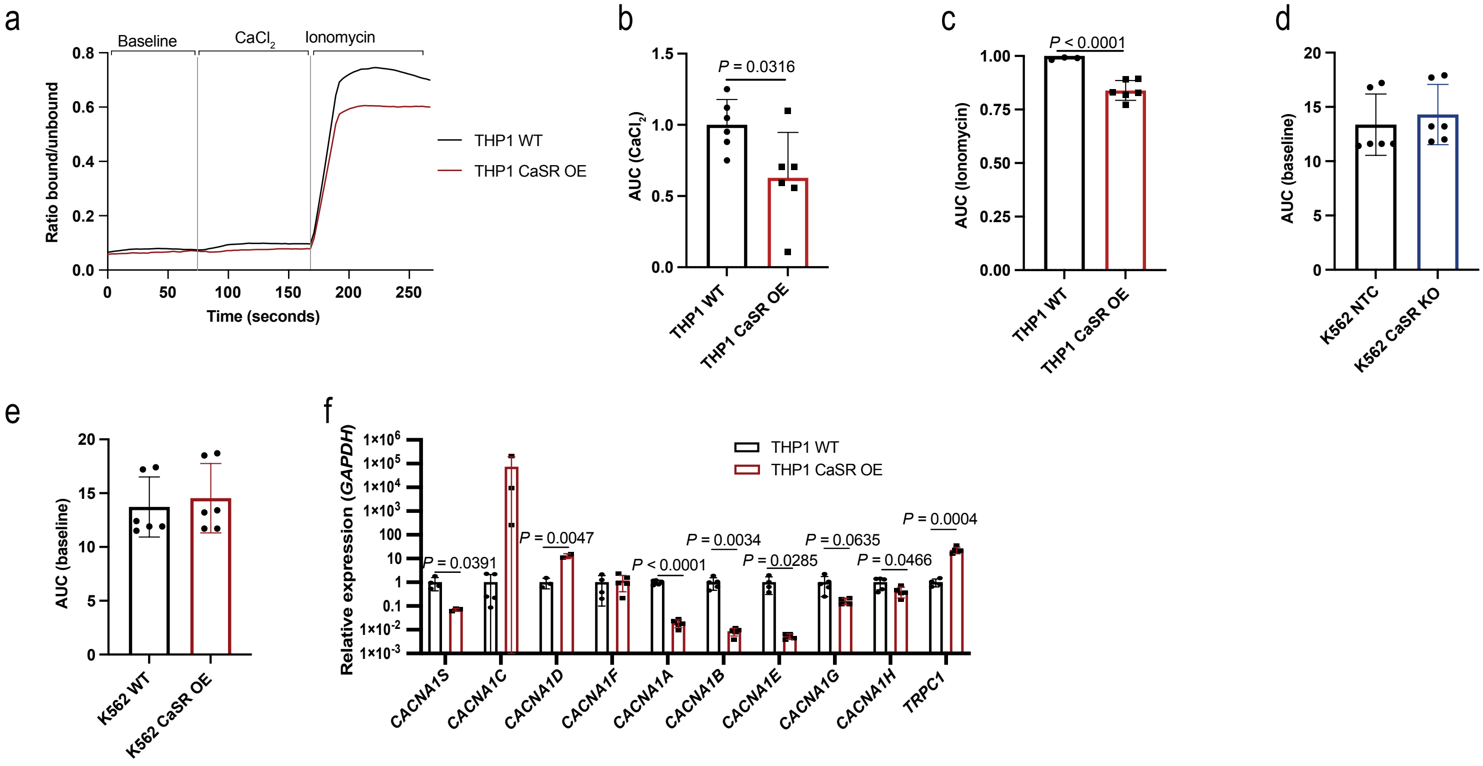

Supplementary Figure 19. a) Calcium flux analysis of THP1 wildtype (WT) (black) and THP1 CaSR -overexpressing (OE) cells (red) using indo-1 dye at baseline, after addition of $CaCl_2$ (5 mM) or ionomycin (1 μg/ml) (n=3). **b-c**) Calcium flux analysis represented by quantification of the area under the curve (AUC) after addition of 5 mM $CaCl_2$ (**b**) and after addition of ionomycin (1 μg/ml) (**c**) of THP1 WT (black) and THP1 CaSR OE cells (red) (n=6, two-tailed *t*-test). **d-e)** Calcium flux analysis represented by quantification of the area under the curve (AUC) at baseline in K562 non-target control (NTC) (black) versus CaSR knockout (KO) cells (blue) (**d**) and K562 WT (black) versus K562 CaSR -overexpressing (OE) cells (red) (**e**) (n=6, two-tailed *t*-test)**. f**) Relative expression of various subunits (S, C, D, F, A, B, E, G, H) of calcium voltage-gated channels (*CACNA1*) and transient receptor potential cation channel subfamily C (*TRPC1*) in WT and CaSR OE THP1 cells by RT-qPCR analysis (n=3-5, two-way ANOVA, Sidak´s test).

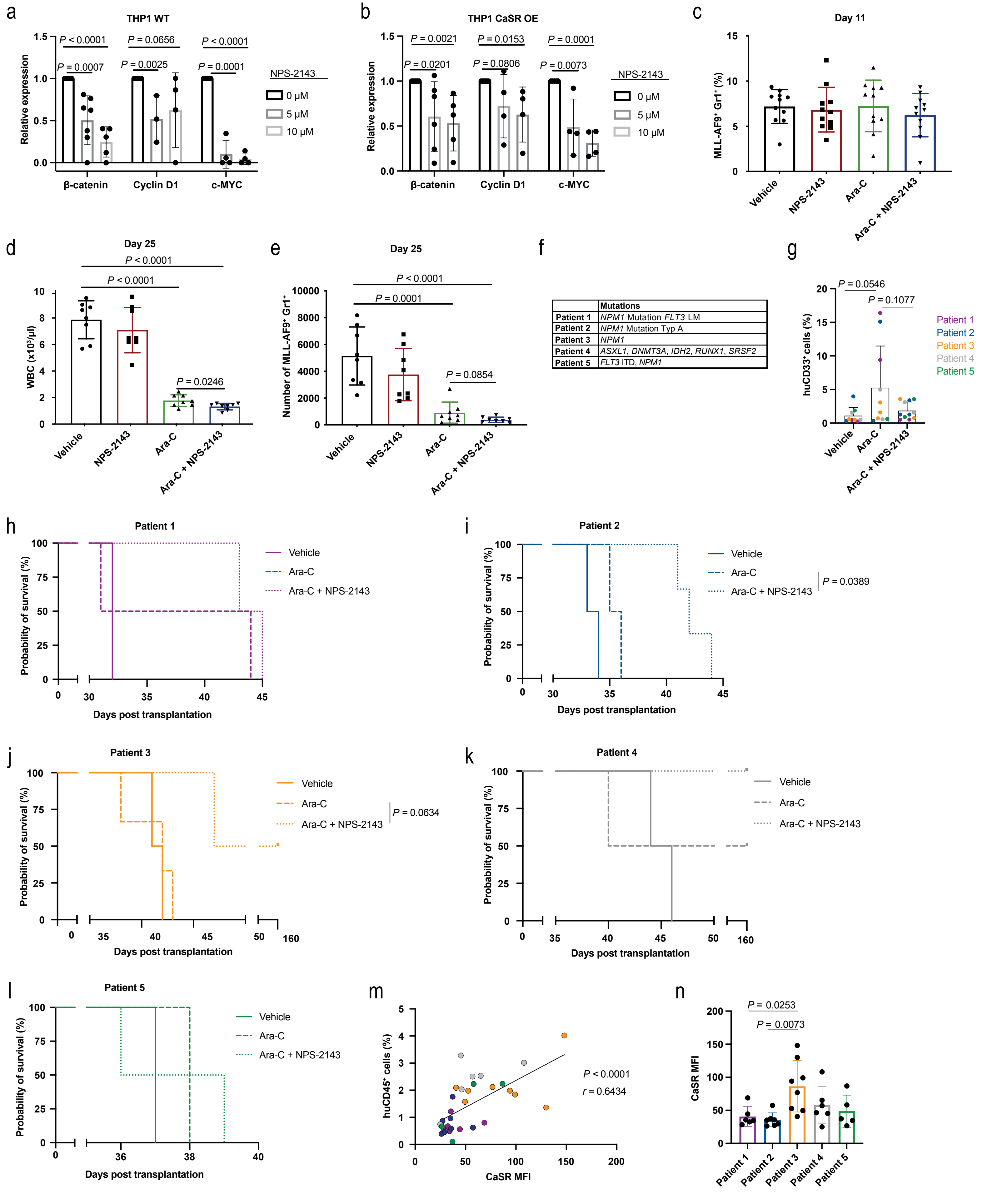

	Mutations
Patient 1	*NPM1* Mutation *FLT3*-LM
Patient 2	*NPM1* Mutation Typ A
Patient 3	*NPM1*
Patient 4	*ASXL1, DNMT3A, IDH2, RUNX1, SRSF2*
Patient 5	*FLT3*-ITD, *NPM1*

Supplementary Figure 20. a-b) Quantification of the band intensity of β-catenin, c-MYC and cyclin D1 in lysates of WT (**a**) versus CaSR OE (**b**) THP1 cells, normalised by GAPDH, in the immunoblots shown in Figure 5b-d (n=3-7, one-way ANOVA, Tukey test). **c**) Percentage of MLL-AF9⁺ (GFP⁺) Gr1⁺ cells of single cells in the peripheral blood of BALB/c mice with MLL-AF9-induced AML prior to treatment,

i.e. on day 11 after transplant (n=8, one-way ANOVA,Tukey test). **d-e**) White blood cell count (WBC) (**d**) and number of MLL-AF9$^+$ (GFP$^+$) Gr1$^+$ cells per 15 μl (**e**) in peripheral blood of BALB/c mice with MLL-AF9-induced AML 25 days after transplantation (n=8, one-way ANOVA, Tukey test). **f**) Genetic alterations of the AML patient samples used in the study. *NPM1*=Nucleophosmin 1, *FLT3*=Fms like tyrosine kinase 3, LM=length mutation, ITD=internal tandem duplication, *ASXL1*=ASXL (additional sex combs-like) transcriptional regulator 1, *DNMT3A*=DNA nucleotide methyltransferase 3A, *IDH1/2*=isocitrate dehydrogenase 1/2, *RUNX1*=Runt-related transcription factor 1, *SRSF2*=serine and arginine rich splicing factor 2. **g**) Percentage of human CD33$^+$ cells in peripheral blood of NOD SCID interleukin-2 receptor γ knockout (NSG) transplanted with 2x10^6 human AML cells from 5 different patients (represented by different colours) and treated with vehicle (n=9), ara-C (n=10) (50 mg/kg, administered on three consecutive days per cycle, for three cycles every two weeks, starting on day 17 after transplantation) or the combination of ara-C and NPS-2143 (n=11) (2 mg/kg, administered from day 17 to day 47 after transplantation as a daily regimen), 30 days post transplantation. Recipients of the same human AML sample are indicated by the same colour (one-way ANOVA,Tukey test). **h-l**) Kaplan-Meier-style survival curves of NSG mice transplanted with human AML cells and treated with vehicle (solid line), ara-C (dashed line) or the combination of ara-C and NPS-2143 (dotted line) as in (**g**). Recipients of the same human AML sample are indicated by the same colour: Patient 1 in purple (**h**) (n=2), patient 2 in blue (**i**) (n=2-3), patient 3 in orange (**j**) (n=2-3), patient 4 in grey (**k**) (n=2) and patient 5 in green (**l**) (n=1-2) (Log-rank test). Only P-values for the comparison between ara-C versus ara-C + NPS-2143 are shown if they are significant or show a trend.The number of transplanted mice per cohort depended on the number of available AML cells. **m**) Correlation of human CD45$^+$ cells and median fluorescence intensity (MFI) of CaSR on peripheral blood leukocytes of NSG mice transplanted with human AML cells as in (**g**) 17 days after transplantation, before the initiation of treatment. Pearson's correlation coefficient (r) and P-value are shown. **n**) Mean fluorescence intensity (MFI) of CaSR on peripheral blood leukocytes of NSG mice transplanted with human AML cells, 17 days after transplantation.

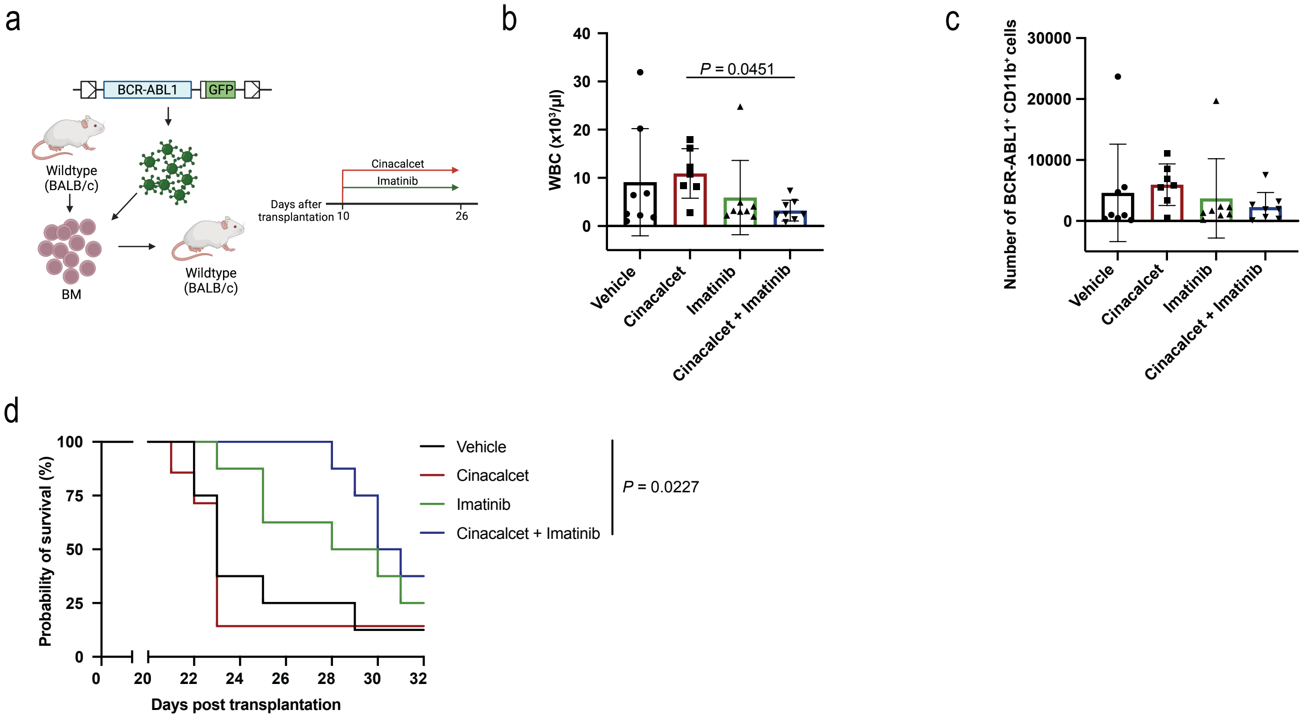

Supplementary Figure 21. a) Treatment scheme for BALB/c mice with BCR-ABL1-induced chronic myeloid leukemia (CML). Mice were randomly assigned to 4 cohorts prior to treatment and treated with vehicle (water), cinacalcet p.o (30 mg/kg administered as a daily regimen), imatinib p.o. (100 mg/kg administered as a daily regimen) or the combination of both cinacalcet and imatinib. Treatment started on day 10 and lasted until day 26 after transplantation. **b-c**) White blood cell count (WBC) (**b**) and number of BCR-ABL1$^+$ (GFP$^+$) CD11b$^+$ cells/15 μl (**c**) in peripheral blood of mice with BCR-ABL1-induced CML and treatment as in (**a**) on day 16 after transplantation (n=7-8, Kruskal-Wallis test, Dunns test). **d**) Kaplan-Meier-style survival curves of mice with CML treated with vehicle (black), cinacalcet (red), imatinib (green) or the combination of both cinacalcet and imatinib (blue) as in (**a**) (n=7-8, Log-rank test).

Supplementary Table 1: Primers used in the generation of CaSR and GCaMP6S overexpression constructs

Primer name	Primer sequence (3' → 5')
hCaSR.XhoI.F	CAGATCTTCCGGATGGCTCGAGT
hCaSR.ApaI.R	ATATATGGGCCCACATCGATTTTCCATGGCAGCTG
hCaSR.seq.1	CTAAGCCTCCGCCTCCTCTTC
hCaSR.seq.2	TGTGCCAGGTGAGTGCCAAG
hCaSR.seq.3	CTTGATCTTTAGCTGCTACTCC
hCaSR.seq.4	CTCACTGAGCTTTGATGAGCT
hCaSR.seq.5	CAGGAGCTGGAGGATGAGAT
hCaSR.Sfi.F	GGCCTCTGAGGCCACCATGGCATTTTATAGCTGCTGCTGGGTCCTCTT
hCaSR.Sfi.R	GGCCTGACAGGCCTTATGAATTCACTACGTTTTCTGTAACAGTGCTGCCT
hCaSR.int.F	CAAGATCTGGCTGGCtAGCGAGGCCT
hCaSR.int.R	AGGCCTCGCTaGCCAGCCAGATCTT
hCaSR.SF1	GTGGGAGCAACTGGCTCA
hCaSR.SF2	TACCTGCTTACCTGGGAGAG
hCaSR.SF3	GGGACCAGGAAAGGGATCAT
hCaSR.SF4	CCTTTGGCATCAGCTTCGT
hCaSR.SF5	GGAAGCTGCCGGAGAACTTC

GCaMP6s.Sfi.F	GGCCTCTGAGGCCACCATGGGTTCTCATCATCATCATCATCATGGT
GCaMP6s.Sfi.R	GGCCTGACAGGCCTCACTTCGCTGTCATCATTTGTACAAACTCT
GCaMP6s.SF1	CCCAACGAGAAGCGCGATCA
GCaMP6s.SF2	GCAGCACGACTTCTTCAAGTC

Supplementary Table 2: Flow cytometry antibodies used in the study.

Antibody	Fluorochrome	Clone	Company	Catalogue number	Dilution
CaSR-Biotin		HL-1499	NOVUS biologicals	#NB-1830	1:100
CaSR	Alexa Fluor 647	5C10	NOVUS biologicals	#NB-120-19347	1:100
CXCR4	PE	2B11	BD Biosciences	#561734	1:100
CD11b	PE	M1/70	Biolegend	#101207	1:100
CD11b (Mac-1)	APC	M1/70	BD Biosciences	#553312	1:100
BP-1 (CD249; Ly-51)	PE	6C3	BD Biosciences	#553735	1:100
Ter119-Biotin			BD Biosciences	#553672	1:100

Ly-6G and Ly-6C (GR1) Biotin		RB6-8C5	BD Biosciences	#553125	1:100
Ly-6G and Ly-6C (GR1)	PE	RB6-8C5	Invitrogen	#12-5931-83	1:100
LY-6G and LY-6C (GR1)	APC-Cy7	RB6-BC5	BD Biosciences	#557661	1:100
CD5-Biotin		53-73	BD Biosciences	#553019	1:100
B220-Biotin		RA3-6B2	BD Biosciences	#553086	1:100
F4/80-Biotin		BM8	eBiosciences	#13-4801-82	1:100
CD117 (c-Kit)	APC	2B8	BD Biosciences	#553356	1:100
CD117 (c-Kit)	PE	2B8	BD Biosciences	#553355	1:100
CD117 (c-Kit)	BV711	2B8	Biolegend	#105835	1:100
Ly6A (Sca-1)	PE-Cy7	D7	Biolegend	#108114	1:100
Ly6A (Sca-1)	PerCP	D7	Biolegend	#108122	1:100
CD34	BV421	RAM34	BD Horizon	#562608	1:100

CD34	APC	RAM34	eBiosciences	#50-0341-82	1:100
CD34	PE	RAM34	BD Biosciences	#551387	1:100
CD16/CD32 (FCγRII)	V450	2.4G2	BD Horizon	#560539	1:100
CD127 (IL7R)	BV711	A7R34	Biolegend	#135035	1:100
Human CD33	PerCP Cyanine5.5	WM53	BioLegend	#303414	1:100
Human CD45	PE	HI30	BD Biosciences	#555483	1:100
Annexin V	APC		BioLegend	#640920	1:100
Ki-67	PE	16A8	BioLegend	#151210	1:100
Ki-67	APC	16A8	BioLegend	#652406	1:100
4′,6-diamidino-2-phenyl-indol-dihydrochlorid (DAPI)			Sigma-Aldrich	#D9542	1:1000
CELLTrace far red			Invitrogen	#C34564	1:1000

p-Histone γH2A.X rabbit mAb (S139)		20E3	Cell Signaling Technology	#9718S	1:100
Goat anti-rabbit IgG (H+L)	Alexa Fluor-647		Invitrogen	#A21244	1:100
INDO-1 AM			Invitrogen	#65-0856-39	1:1000
CELLROX Deep red			Invitrogen	#C10422	250nM

Supplementary Table 3: qPCR primers used in the study.

**Primer name**	**Primer sequence (3' → 5')**	**Species**
**CASR_FW**	GCAACCAGGAGCTGGAGGAT	Mouse/human
**CASR_RV**	CAGGCAGGTGTAGCCGATCA	Mouse/human
**FLNA_FW**	CATCAAGTACGGTGGTGACG	Mouse/human
**FLNA_RV**	ACATCCACCTCTGAGCCATC	Mouse/human
**VDR_FW**	CCAGTTCGTGTGAATGATGG	Mouse/human
**VDR_RV**	AGATTGGAGAAGCTGGACGA	Mouse/human
**HOXA9_FW**	GGTTCTCCTCCAGTTGATAGAGA	Mouse

**HOXA9_RV**	GAGCGAGCATGTAGCCAGTTG	Mouse
**MEIS1_FW**	ACGGCATCCACTCGTTCA	Mouse
**MEIS1_RV**	TGGCTGTCCATCAGGGTT	Mouse
**RUNX1_FW**	GATGGCACTCTGGTCACCG	Mouse
**RUNX1_RV**	GCCGCTCGGAAAAGGACAA	Mouse
**CREB_FW**	AGCAGCTCATGCAACATCATC	Mouse
**CREB_RV**	AGTCCTTACAGGAAGACTGAACT	Mouse
**HOXB4_FW**	CGTGAGCACGGTAAACCCC	Mouse
**HOXB4_RV**	GTGTTGGGCAACTTGTGGTC	Mouse
**ALOX5_FW**	CTACGATGTCACCGTGGATG	Mouse
**ALOX5_RV**	GTGCTGCTTGAGGATGTGAA	Mouse
**EZH2_FW**	GTGACCACAGGATAGGCATCT	Mouse
**EZH2_RV**	CAAGGGATTTCCATTTCTCG	Mouse
**GATA-1_FW**	CCCACCTCTATCAGG GCCTA	Mouse/human
**GATA-1_RV**	GAGGTTGTAGGCGAT CCCAG	Mouse/human
**EVI1_FW**	CTGGCTGCTGCTGATCTTAT	Mouse/human
**EVI1_RV**	AGTTCGCTTACCTTCCGATTT	Mouse/human
**MYC_FW**	CCTGGTGCTCCATGAGGAGAC	Human

**MYC_RV**	CAGACTCTGACCTTTTGCCAGG	Human
**C-JUN_FW**	ATCAAGGCGGAGAGGAAGCG	Human
**C-JUN_RV**	TGAGCATGTTGGCCGTGGAC	Human
**C-FOS_FW**	CTGGCGTTGTGAAGACCAT	Human
**C-FOS_RV**	TCCCTTCGGATTCTCCTTTT	Human
**ETS_FW**	GTGCTGTCAGGCGTTCT	Human
**ETS_RV**	GGGAGAGGAGCAGACGAG	Human
**ELK1_FW**	CGCAAGAACAAGACCAACATG	Human
**ELK1_RV**	TCAGGGTAGGACACAAACTTG	Human
**BCL-XL_FW**	GGAGAACGGCGGCTGGGATA	Human
**BCL-XL_RV**	GGCCACAGTCATGCCCGTCA	Human
**CACNA1-A_FW**	CTGGTAGCCTTTGCCTTCACTG	Mouse/human
**CACNA1-A_RV**	ACACAGCCTTGAGCTTTGGCAG	Mouse/human
**CACNA1-B_FW**	TGGACCTGGAAAGCCAAGCAGA	Human
**CACNA1-B_RV**	GGTCACGATGTAGTGGCAGAAG	Human
**CACNA1-C_FW**	GCAGGAGTACAAGAACTGTGAGC	Mouse/human
**CACNA1-C_RV**	CGAAGTAGGTGGAGTTGACCAC	Mouse/human
**CACNA1-D_FW**	CTTCGACAACGTCCTCTCTGCT	Human

**CACNA1-D_RV**	GCCGATGTTCTCTCCATTCGAG	Human
**CACNA1-E_FW**	AGCGTGAGACAGGCAAAGCCAT	Human
**CACNA1-E_RV**	GGATGCACATCTCAAAGTAGCGC	Human
**CACNA1-F_FW**	CCCTGATCGGCTTTCCTACCTAGATGA	Human
**CACNA1-F_RV**	CAGGCACGTGCAGACAGGTGAA	Human
**CACNA1-G_FW**	TTCACCGCAGTCTTTCTGGCTG	Human
**CACNA1-G_RV**	TGACGGAGATGAGCACCAACAG	Human
**CACNA1-H_FW**	GGAACATCTCCACCAAGGCACA	Human
**CACNA1-H_RV**	TCCATCCTTGGATGACAGCACG	Human
**CACNA1-S_FW**	GACCACTCAGAGCCATCAACAG	Human
**CACNA1-S_RV**	GGTAGTGACCAGCACGATGTTC	Human
**TRPC4_FW**	CCAAACTGCTGGTGGCAATGCT	Human
**TRPC4_RV**	GGAATGATGTTGAAAGGTGGAGG	Human
**CXCL12_FW**	CTCAACACTCCAAACTGTGCCC	Human
**CXCL12_RV**	CTCCAGGTACTCCTGAATCCAC	Human
**GAPDH_FW**	AGGTCGGTGTGAACGGATTTG	Mouse/human
**GAPDH_RV**	GGGGTCGTTGATGGCAACA	Mouse/human

Supplementary Table 4: Primers used in the cloning of sgRNA constructs

Primer name	Primer sequence (3' → 5')
hCaSR 1 s	CACCGCTGTAACCAGCGAAACCCA
hCaSR 1 as	AAACTGGGTTTCGCTGGTTACAGC
hFLNA ex2 s	CACCGCCTGGCGGAGGACGCGCCG
hFLNA ex2 as	AAACCGGCGCGTCCTCCGCCAGGC
NTC s	CACCGTTCCGGGCTAACAAGTCCT
NTC as	AAACAGGACTTGTTAGCCCGGAAC
LKO.1	GACTATCATATGCTTACCGT

Supplementary Table 5: Western Blotting antibodies used in the study.

Antibody		Company	Catalogue number	Dilution
GAPDH	Mouse monoclonal	Santa Cruz Biotechnology	#Sc-32233	1:1000
CaSR	Mouse monoclonal	Santa Cruz Biotechnology	#Sc-47741	1:400
Filamin-1	Mouse monoclonal	Santa Cruz Biotechnology	#Sc-17749	1:500
Filamin A	Rabbit Ab	Cell Signaling Technology	#4762S	1:1000
ERK1/2	Mouse monoclonal	Santa Cruz Biotechnology	#Sc-514302	1:500
p-ERK	Mouse monoclonal	Santa Cruz Biotechnology	#Sc-7383	1:500
Cyclin D1	Rabbit Ab	Cell Signaling Technology	#E3P5S	1:800

β-catenin	Rabbit polyclonal IgG	EMD Millipore Corp, Merck	#2942488	1:1000
c-Myc	Rabbit mAb	Cell Signaling Technology	#E5Q6W	1:800
CXCR4	Rabbit polyclonal	Invitrogen	#PA3-305	1:1000
anti-mouse IgG HRP-linked ab		Cell Signaling Technology	#34	1:5000
anti-rabbit IgG HRP-linked ab		Cell Signaling Technology	#29	1:5000

Supplementary Table 6: Immunofluorescence antibodies used in the study.

**Antibody**		**Company**	**Catalogue number**	**Dilution**
CaSR	Mouse Ab	Abcam	#GR3309582-6	1:50
Filamin-1	Mouse Ab	Santa Cruz Biotechnology	#Sc-17749	1:25
Filamin A	Rabbit Ab	Cell Signaling Technology	#4762S	1:50

p-histone γH2A.X (S139)	Rabbit Ab	Cell Signaling Technology	#9718S	1:100
Alexa fluor 488	Goat anti-mouse IgG (H+L)	Invitrogen	#A11029	1:400
Alexa fluor plus 647	Goat anti-mouse IgG (H+L)	Invitrogen	#A32728	1:400
Alexa fluor 647	Goat anti-rabbit IgG (H+L)	Invitrogen	#A21244	1:400

Supplementary Table 7: Patient samples used in the study.

	Diagnosis	Subtype (FAB)	Initial diagnosis	Treatment	Source
**Patient 1**	AML	M2	Yes	No	BM
**Patient 2**	AML	M4	Yes	No	BM
**Patient 3**	AML		Yes	No	BM
**Patient 4**	AML	M4	Yes	No	BM
**Patient 5**	AML		Yes	No	BM
