# Supplementary material for: Distinct and targetable role of calcium-sensing receptor in leukaemia
Source: Nat Commun. 2023 Oct 6;14:6242. doi: 10.1038/s41467-023-41770-0 (PMC10558580; doi:10.1038/s41467-023-41770-0)
Supplement: Supplementary file 2 — Reporting Summary [file 41467_2023_41770_MOESM2_ESM.pdf]

nature portfolio

Corresponding author(s): Daniela S. Krause

Last updated by author(s): 17/06/2023

Reporting Summary

Nature Portfolio wishes to improve the reproducibility of the work that we publish. This form provides structure for consistency and transparency in reporting. For further information on Nature Portfolio policies, see our Editorial Policies and the Editorial Policy Checklist.

Statistics

For all statistical analyses, confirm that the following items are present in the figure legend, table legend, main text, or Methods section.

n/a	Confirmed	
☐	☒	The exact sample size (n) for each experimental group/condition, given as a discrete number and unit of measurement
☐	☒	A statement on whether measurements were taken from distinct samples or whether the same sample was measured repeatedly
☐	☒	The statistical test(s) used AND whether they are one- or two-sided<br>*Only common tests should be described solely by name; describe more complex techniques in the Methods section.*
☐	☒	A description of all covariates tested
☐	☒	A description of any assumptions or corrections, such as tests of normality and adjustment for multiple comparisons
☐	☒	A full description of the statistical parameters including central tendency (e.g. means) or other basic estimates (e.g. regression coefficient) AND variation (e.g. standard deviation) or associated estimates of uncertainty (e.g. confidence intervals)
☐	☒	For null hypothesis testing, the test statistic (e.g. F, t, r) with confidence intervals, effect sizes, degrees of freedom and P value noted<br>*Give P values as exact values whenever suitable.*
☒	☐	For Bayesian analysis, information on the choice of priors and Markov chain Monte Carlo settings
☒	☐	For hierarchical and complex designs, identification of the appropriate level for tests and full reporting of outcomes
☐	☒	Estimates of effect sizes (e.g. Cohen's d, Pearson's r), indicating how they were calculated

Our web collection on statistics for biologists contains articles on many of the points above.

Software and code

Policy information about availability of computer code

Data collection	For flow cytometry we used FlowJo software 10.5.3.
Data analysis	We used GraphPad Prism vs 9 (GraphPad Software, Inc., San Diego, CA) to perform the statistical analysis.<br>FlowJo Software version 10.5.3<br>FACSDiva 9.0.<br>Image J 1.53t

For manuscripts utilizing custom algorithms or software that are central to the research but not yet described in published literature, software must be made available to editors and reviewers. We strongly encourage code deposition in a community repository (e.g. GitHub). See the Nature Portfolio guidelines for submitting code & software for further information.

Data

Policy information about availability of data

All manuscripts must include a data availability statement. This statement should provide the following information, where applicable:

- Accession codes, unique identifiers, or web links for publicly available datasets
- A description of any restrictions on data availability
- For clinical datasets or third party data, please ensure that the statement adheres to our policy

Source data are provided with this paper. RNA sequencing data are available at Gene Expression Omnibus (GEO) under accession numbers GSE208239. All mass

spectrometry proteomics data have been deposited in the ProteomeXchange Consortium 63 via the PRIDE partner repository 64 with the dataset identifier PXD035937.
Data on expression of genes in human AML samples were obtained from the publicly available dataset TCGA, accessed through the cBioPortal portal (https://www.cbioportal.org).
Supplementary information is available for this paper.
Correspondence and requests for materials should be addressed to the corresponding author.
Reprints and permissions information is available at www.nature.com/reprints.

Human research participants

Policy information about studies involving human research participants and Sex and Gender in Research.

Reporting on sex and gender	Both sexes were used. We did not notice any sex-dependent differences.
Population characteristics	The patients were between the ages of 57-76.
Recruitment	N/A
Ethics oversight	The use of BM samples from male and female patients with AML for xenotransplantation experiments was approved by the medical ethics committee II of the Medical Faculty of the University of Heidelberg (Approval number 2013 509N MA).<br>The supernatants of bone marrow aspirates from patients with different haematological malignancies were provided by the biobank of the University Center for Tumoral Diseases in Frankfurt am Main. These experiments were approved by the Ethics Committee of the University Clinic of the Goethe University Frankfurt (Approval number 274/18 and SHN 5 2020). Informed consent was obtained.

Note that full information on the approval of the study protocol must also be provided in the manuscript.

Field-specific reporting

Please select the one below that is the best fit for your research. If you are not sure, read the appropriate sections before making your selection.

- [x] Life sciences
- [ ] Behavioural & social sciences
- [ ] Ecological, evolutionary & environmental sciences

For a reference copy of the document with all sections, see nature.com/documents/nr-reporting-summary-flat.pdf

Life sciences study design

All studies must disclose on these points even when the disclosure is negative.

Sample size	For experimental design, we perform power analysis, typically specifying P = 0.05, two-tailed testing, and power (= 1-β) of 80% (ie, given a particular difference in groups, we want an 80% chance of getting statistically significant results), using commercially available software packages (Statistical Solutions nQuery Advisor; http://www.statsol.ie/nquery/nquery.htm). Although these conditions vary, our control cohorts are usually designed to have fairly tight incidence curves, allowing us to be adequately powered with experimental recipient cohorts of average size n =10.
Data exclusions	No data were excluded from the analysis.
Replication	All in vitro experiments were performed using at least 3 biological replicates. All experiments have been repeated independently with similar results. All other experiments were repeated 3-5 times. All attempts at replication were successful.
Randomization	In mouse experiments, animals were randomly assigned to experimental groups. For in vitro studies, samples were randomly allocated to experimental groups.
Blinding	The investigators were blinded to group allocation during data collection and analysis.

Reporting for specific materials, systems and methods

We require information from authors about some types of materials, experimental systems and methods used in many studies. Here, indicate whether each material, system or method listed is relevant to your study. If you are not sure if a list item applies to your research, read the appropriate section before selecting a response.

Materials & experimental systems

n/a	Involved in the study
	☒ Antibodies
	☒ Eukaryotic cell lines
☒	Palaeontology and archaeology
	☒ Animals and other organisms
☒	Clinical data
☒	Dual use research of concern

Methods

n/a	Involved in the study
☒	ChIP-seq
	☒ Flow cytometry
☒	MRI-based neuroimaging

Antibodies

Antibodies used

Biotin mouse monoclonal CaSR (HL-1499) NOVUS biologicals #NB-18309 (1:100)
AF647 mouse monoclonal CaSR (HL-1499) NOVUS biologicals #NB-120-19347(1:100)
PE Rat monoclonal anti-mouse CXCR4/CD184 (2B11) BD Pharmingen #61734 (1:100)
PE Rat monoclonal anti-mouse CD11b (M1/70) BioLegend #101207 (1:100)
APC Rat monoclonal anti-mouse CD11b (M1/70) BD Pharmingen #553312 (1:100)
PE Rat monoclonal anti-mouse Ly-51 (BP1) (6C3) BioLegend #108308 (1:100)
Biotin Rat monoclonal anti-mouse Ter119 BD Pharmingen #553672 (1:100)
Biotin Rat monoclonal anti-mouse CD5 (53-73) BD Pharmingen #553019 (1:100)
Biotin Rat monoclonal F4/80 (BM8) eBioscience #13-4801-82 (1:100)
Biotin Rat monoclonal anti-mouse CD45R/B220 (RA3-6B2) BD Pharmingen #553086 (1:100)
Biotin Rat monoclonal Ly-6G and Ly-6c (Gr1) (RB6-8C5) BD Pharmingen #553125 (1:100)
APC-Cy7 Rat monoclonal Ly-6G and Ly-6c (Gr1) (RB6-8C5) BD Pharmingen #557661 (1:100)
PE Rat monoclonal Ly-6G and Ly-6c (Gr1) (RB6-8C5) Invitrogen #12-5931-83 (1:100)
APC Rat monoclonal anti-mouse c-Kit (2B8) BD Pharmingen #553356 (1:100)
PE Rat monoclonal anti-mouse c-Kit (2B8) BD Pharmingen #553355 (1:100)
BV711 Rat monoclonal anti-mouse c-Kit (2B8) Biolegend #105835 (1:100)
PE-Cy7 Rat monoclonal anti-mouse Sca-1 (D7) BioLegend #108114 (1:100)
PerCP Rat monoclonal anti-mouse Sca-1 (D7) BioLegend #108122 (1:100)
PE Rat monoclonal anti-mouse CD34 (RAM34) BD Pharmingen #551387 (1:100)
APC Rat monoclonal anti-mouse CD34 (RAM34) eBioscience #50-0341-82 (1:100)
BV421 Rat monoclonal anti-mouse CD34 (RAM34) BD Pharmingen #562608 (1:100)
V450 Rat monoclonal anti-mouse CD16/32 (FCγRIII/ FCγRII) (2.4G2) BD Pharmingen #560539 (1:100)
BV711 Rat monoclonal anti-mouse CD127 (IL7Rα) (A7R34) BioLegend #135035 (1:100)
FITC Rat monoclonal anti-mouse Ter119 BD Pharmingen #561032 (1:100)
PB B220 Rat monoclonal anti-mouse CD45R/B220 (RA3-6B2) BD Pharmingen #558108 (1:100)
APC-Cy7 Streptavidin BioLegend #405208 (1:100)
PerCP Cyanine5.5 Human CD33 (WM53) BioLegend #03414 (1:100)
PE Mouse monoclonal anti-human CD45 (HI30) BD Pharmingen #555483(1:100)
PE Rat monoclonal anti-mouse Ki-67 (16A8) BioLegend #652404 (1:100)
APC Rat monoclonal anti-mouse Ki-67 (16A8) BioLegend #652406 (1:100)
Rabbit monoclonal anti-mouse anti-GAPDH (6C5) Santa Cruz #sc-32233 (WB 1:1000)
Mouse monoclonal anti-mouse/human anti-CaSR (6D4) Santa Cruz #sc-47741 (WB 1:400)
Mouse monoclonal anti-mouse/human anti-Filamin-1 (E-3) Santa Cruz #sc-17749 (WB 1:500, IF 1:25)
Rabbit polyclonal anti-mouse/human anti-Filamin A Cell Signaling #4762 (WB 1:1000, IF 1:50)
Mouse monoclonal anti-mouse/human anti-ERK1/2 (C-9) Santa Cruz #sc-514302 (WB 1:500)
Mouse monoclonal anti-mouse/human anti-p-ERK (Thr202/Tyr204)(E-4) Santa Cruz #sc-7383 (WB 1:500)
Rabbit monoclonal anti-mouse/human anti-cyclin D1 (E3P5S) Cell Signaling #55506 (WB 1:800)
Rabbit polyclonal anti-mouse/human anti-β-catenin (06-734) Millipore #06-734 (WB 1:1000)
Rabbit monoclonal anti-mouse/human anti-c-Myc (E5Q6W) Cell Signaling #18583 (WB 1:800)
Rabbit polyclonal anti-mouse/human anti-CXCR4 (CD184) Invitrogen #PA3-305 (WB 1:1000)
HRP-linked anti-rabbit IgG Cell Signaling #7074 (WB 1:5000)
HRP-linked anti-mouse IgG Cell Signaling #7076 (WB 1:5000)
Alexa Fluor 488 donkey polyclonal anti-rabbit Invitrogen #A-21206 (IF 1:400)
Alexa Fluor 647 goat polyclonal anti-rabbit Invitrogen #A-21244 (IF 1:400)
Alexa fluor plus 647 goat polyclonal anti-rabbit Invitrogen #A32728 (IF 1:400)
Mouse monoclonal anti-mouse/human anti-CaSR (5C10) Abcam #Ab19347 (IF 1:50)
phospho-Histone γH2A.X rabbit mAb (S139) (20E3) Cell Signaling #9718 (IF 1:100)
Mouse monoclonal anti-mouse/human Vinculin (VLN01) Invitrogen #MA5-11690 (WB 1:1000)
Mouse monoclonal anti-mouse/human Actin Sigma-Aldrich #A4700-100UL (IF 1:1000)

Validation

The antibodies were validated for their species and application, either as stated in the data sheet of the antibody by the respective manufacturers, or by us, as shown in the manuscript or in pilot experiments.

Eukaryotic cell lines

Policy information about cell lines and Sex and Gender in Research

Cell line source(s)	All cell lines were obtained from DSMZ (German Collection of Microorganisms and Cell Cultures GmbH) or ATCC (American Type Culture Collection).<br>The cell lines used in this study were THP1 (ACC16) , K562 (ACC10), Nalm-6 (ACC128), Kasumi (ACC220), 293T (ACC635), NIH/3T3 (ACC59), BA/F3 (ACC300), HS-5 (CRL-3611).
Authentication	After purchase from DSMZ or ATCC the cells were not reauthenticated.
Mycoplasma contamination	Cells were regularly tested for mycoplasma contamination and were only used for experiments, if free of mycoplasma contamination.
Commonly misidentified lines (See ICLAC register)	No commonly misidentified lines were used.

Animals and other research organisms

Policy information about studies involving animals; ARRIVE guidelines recommended for reporting animal research, and Sex and Gender in Research

Laboratory animals	BALB/c, Mx1-cre (BL/6), CaSR flox/flox (BL/6), Col1-caPPR (FVB), NOD SCID IL2Rγ KO (NSG), Rag-2-/- IL-2R gamma-/- CD47-/- mice were used at an age of 8-14 weeks.<br>Generation of haematopoietic-specific inducible CaSR knockout: CaSR flox/flox mice were crossed with MX1-cre+/- mice to generate CaSR flox/flox MX1-Cre+/- mice in C57BL6 genetic background.<br>The mice were housed in individually ventilated cages at an ambient temperature between 20-26 degrees Celsius. A 14 hour light/10 hour dark cycle was used.
Wild animals	The study did not involve wild animals.
Reporting on sex	Animals of different sexes were randomly assigned to experimental groups, while ensuring that an even distribution of gender was found amongst all cohorts.
Field-collected samples	The study did not involve field-collected samples.
Ethics oversight	All animal studies were performed in compliance with the guiding principles of the 'Guide for the Care and Use of Laboratory Animals' and approved by the local government (Regierungspräsidium Darmstadt, Germany), protocol number

Note that full information on the approval of the study protocol must also be provided in the manuscript.

Flow Cytometry

Plots

Confirm that:

- [x] The axis labels state the marker and fluorochrome used (e.g. CD4-FITC).
- [x] The axis scales are clearly visible. Include numbers along axes only for bottom left plot of group (a 'group' is an analysis of identical markers).
- [x] All plots are contour plots with outliers or pseudocolor plots.
- [x] A numerical value for number of cells or percentage (with statistics) is provided.

Methodology

Sample preparation	Cells cultured in suspension were harvested and centrifuged at 1200rpm for 5 minutes and washed with cell staining buffer to remove residual growth factors that may be present in the culture medium.<br><br>Adherent cell lines were treated with trypsin and further incubated in cell culture medium. Cells were centrifuged at 1200rpm for 5 minutes and washed with cell staining buffer by centrifugation.<br><br>The pellet of either suspension or adherent cells was resuspended in cell staining buffer and the concentration adjusted to 1 x 106 cells/mL in cell staining buffer.
Instrument	BD Fortessa (BD Biosciences), Heidelberg, Germany.
Software	FlowJo_v10.5.3 (Java Version: 1.8.0_151-b12).
Cell population abundance	The proportion of each cell type/protein expression was assessed by flow cytometry, in which target cells were labeled with

	fluorescent antibodies and analyzed using a flow cytometer. Sufficient number of cells were used in each experiment.
Gating strategy	Forward versus side scatter (FSC vs SSC) gating was used to identify cells of interest based on size and granularity (complexity).<br>Next, the forward scatter height (FSC-H) vs. forward scatter area (FSC-A) density or side scatter height (SSC-H) vs side scatter area (SSC-A) plot was used to exclude doublets.<br>Then, unstained and single control samples were used to further identify distinct cells that express a particular marker in a specific population of cells.

☒ Tick this box to confirm that a figure exemplifying the gating strategy is provided in the Supplementary Information.
